# COX17 acetylation via MOF–KANSL complex promotes mitochondrial integrity and function

**DOI:** 10.1038/s42255-023-00904-w

**Published:** 2023-10-09

**Authors:** Sukanya Guhathakurta, Niyazi Umut Erdogdu, Juliane J. Hoffmann, Iga Grzadzielewska, Alexander Schendzielorz, Janine Seyfferth, Christoph U. Mårtensson, Mauro Corrado, Adam Karoutas, Bettina Warscheid, Nikolaus Pfanner, Thomas Becker, Asifa Akhtar

**Affiliations:** 1 Max Planck Institute of Immunobiology and Epigenetics, Freiburg, Germany; 2 Faculty of Biology, University of Freiburg, Freiburg, Germany; 3 Institute of Biochemistry and Molecular Biology, Faculty of Medicine, University of Bonn, Bonn, Germany; 4 Institute of Biology II, Faculty of Biology, University of Freiburg, Freiburg, Germany; 5 Institute of Biochemistry and Molecular Biology, Faculty of Medicine, University of Freiburg, Freiburg, Germany; 6 Cologne Excellence Cluster on Cellular Stress Responses in Aging-Associated Diseases, University of Cologne, Cologne, Germany; 7 Center for Molecular Medicine Cologne (CMMC), University of Cologne, Cologne, Germany; 8 Institute for Genetics, University of Cologne, Cologne, Germany; 9 Signaling Research Centers BIOSS and CIBSS, University of Freiburg, Freiburg, Germany; 10 Theodor Boveri-Institute, University of Würzburg, Würzburg, Germany

**Keywords:** Epigenetics, Acetylation, Acetyltransferases, Metabolism, Mitochondria

## Abstract

Reversible acetylation of mitochondrial proteins is a regulatory mechanism central to adaptive metabolic responses. Yet, how such functionally relevant protein acetylation is achieved remains unexplored. Here we reveal an unprecedented role of the MYST family lysine acetyltransferase MOF in energy metabolism via mitochondrial protein acetylation. Loss of MOF–KANSL complex members leads to mitochondrial defects including fragmentation, reduced cristae density and impaired mitochondrial electron transport chain complex IV integrity in primary mouse embryonic fibroblasts. We demonstrate COX17, a complex IV assembly factor, as a bona fide acetylation target of MOF. Loss of COX17 or expression of its non-acetylatable mutant phenocopies the mitochondrial defects observed upon MOF depletion. The acetylation-mimetic COX17 rescues these defects and maintains complex IV activity even in the absence of MOF, suggesting an activatory role of mitochondrial electron transport chain protein acetylation. Fibroblasts from patients with MOF syndrome who have intellectual disability also revealed respiratory defects that could be restored by alternative oxidase, acetylation-mimetic COX17 or mitochondrially targeted MOF. Overall, our findings highlight the critical role of MOF–KANSL complex in mitochondrial physiology and provide new insights into MOF syndrome.

## Abstract (web summary)

Guhathakurta et al. describe the acetyltransferase activity of MOF in the mitochondria. MOF can acetylate COX17, thus contributing to the assembly and function of respiratory complex IV. These findings provide better understanding of how mitochondrial function is fine-tuned by acetylation.

## Main

Cellular metabolism is tightly regulated by the collaborative and synchronized interactions between mitochondria and the nucleus. While many genes encoding mitochondrial enzymes reside in the nuclear genome, mitochondria are the main production site for key metabolites that not only fulfil energy demands for nuclear processes but also serve as building blocks required for the formation of genetic and epigenetic landscapes. This inter-organellar anterograde and retrograde communication is aided by proteins that shuttle between these two compartments. In response to specific signals, metabolic enzymes such as the tricarboxylic acid cycle component pyruvate dehydrogenase complex translocate from the mitochondrial matrix to the nucleus to provide acetyl-CoA for histone acetylation^1^. Among many other epigenetic regulators, histone deacetylase 3 (HDAC3), a predominantly nuclear protein, was shown to translocate to the mitochondria upon NLRP3 inflammasome activation and deacetylate mitochondrial trifunctional enzyme subunit (HADHA) restraining its enzyme activity^2^.

Protein acetylation has emerged as one of the common regulatory mechanisms in both nuclear and mitochondrial compartments^3,4^. Acetylation of lysine residues on histone tails is generally correlated with activation of gene expression, whereas that on the majority of the mitochondrial metabolic enzymes leads to their inactivation^5^. In contrast to reversible histone acetylation with well-established writers and erasers, mitochondrial protein acetylation is suggested to be non-enzymatic and majorly regulated by deacetylation that is under the control of the NAD^+^-dependent deacetylase SIRT3 (refs. ^6–9^). However, a comprehensive picture of the functional consequences of specific protein acetylation in mitochondria and mitochondrial lysine acetyltransferases with defined substrate repertoires are still missing.

We recently found that the lysine acetyltransferase (KAT) MYST1/KAT8/MOF dually localizes to the nucleus and mitochondria of human cancer cells^10^. MOF is involved in two independent axes for its regulation of cellular metabolism. In mitochondria of cancer cells, MOF and its KANSL complex partner KANSL1 were shown to be required for transcription of the mitochondrial genome-encoded oxidative phosphorylation (OXPHOS) system subunits under metabolic stress^10^. In the nucleus, MOF is the primary enzyme responsible for the acetylation of histone H4 lysine 16 (H4K16)^11^. Loss of MOF–KANSL complex members in embryonic neurons results in detrimental alterations to the neural metabolic environment and low dosage of MOF leads to impaired glucose metabolism in adults^12,13^. Furthermore, MOF was shown to activate fatty acid oxidation in ground state embryonic stem cells, which particularly rely on it for their mitochondrial respiration. In this case, deletion of *Mof* or inhibition of fatty acid oxidation leads to cellular quiescence reminiscent of diapause during embryonic development^14^. In the above cases of metabolic disturbance, the defect could be explained by loss of H4K16 acetylation and transcription of pathway-specific genes^12–14^. Heterozygous de novo point mutations in *MOF* and *KANSL1* result in human developmental anomalies, including intellectual disability, that are remarkably similar to the clinical manifestations of mitochondrial dysfunction^15–20^. However, the underlying molecular mechanism of how the enzymatic activity of the MOF–KANSL complex governs mitochondrial fitness and its relevance in MOF-associated disease pathology remain elusive^21^. In this Article, we elucidate that acetylation of mitochondrial proteins via MOF is instrumental in maintaining mitochondrial integrity and function. We identify COX17 as an evolutionary conserved acetylation target of MOF and show that COX17 acetylation via MOF promotes mitochondrial respiration using primary mouse and human cells.

## Results

### MOF–KANSL complex maintains mitochondrial structure and bioenergetics

**Fig. 1:**
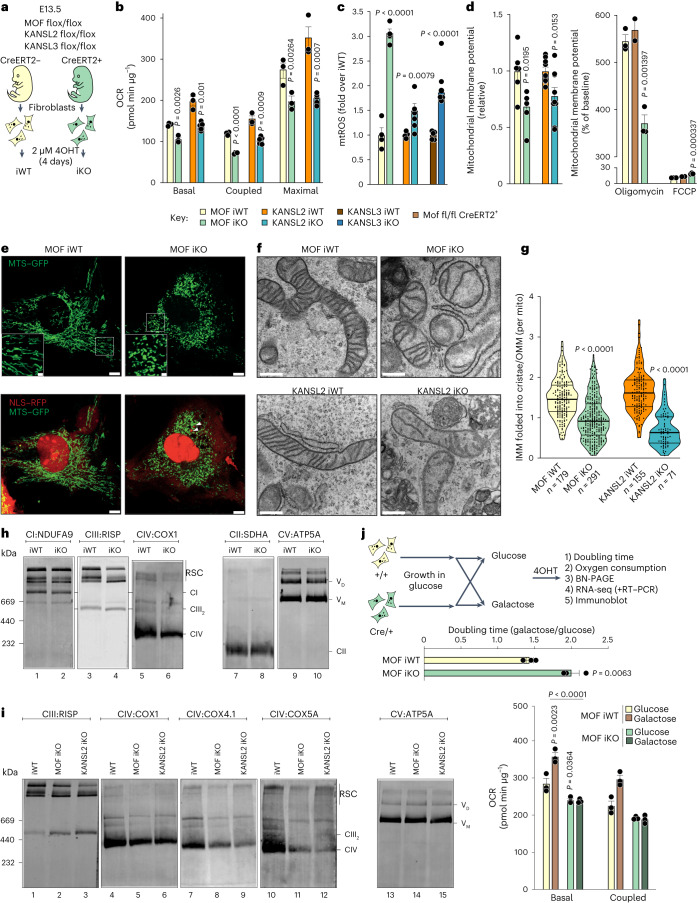
The MOF–KANSL complex maintains mitochondrial structure and function. **a**, Experimental setup for depletion of MOF–KANSL complex members in primary MEFs. iWT, control; iKO, knockout. **b**, OCR in control, *Mof* and *Kansl2* iKO MEFs under indicated states of mitochondrial respiration (mean ± s.e.m., *n* = 3–5 embryos, two-tailed Student’s *t*-test). **c**, Flow cytometric analysis of mtROS by mitoSOX dye in control, *Mof*, *Kansl2* and *Kansl3* iKO MEFs (mean ± s.e.m., *n* = 3–5 embryos, normalized to the mean of the corresponding iWT control, two-tailed Student’s *t*-test). **d**, Flow cytometric analysis of mitochondrial membrane potential by TMRM dye in control, *Mof* and *Kansl2* iKO MEFs (left) and in control and *Mof* iKO MEFs treated with 1 µM oligomycin or 15 µM FCCP (right) (mean ± s.e.m., *n* = 3–6 embryos, normalized to the mean of the corresponding iWT control, two-tailed Student’s *t*-test). **e**, Fluorescence microscopy images of control and *Mof* iKO MEFs expressing mitochondrial GFP (MTS–GFP) and nuclear RFP (NLS–RFP). White arrowheads: micronuclei, validating MOF depletion in the corresponding cell. Scale bars, 10 µm (insets 1 µm). Quantification of *n* ≥ 100 cells is shown in Extended Data Fig. 2a. **f**,**g**, High-magnification electron micrograph of mitochondria (**f**) and quantification of length of IMM folded into cristae, normalized to the outer mitochondrial membrane (OMM) perimeter of individual mitochondria (**g**) from control, *Mof* and *Kansl2* iKO MEFs (*n* = 3 embryos). Scale bars, 500 nm. Violin plots show all data points with lines at the median and first and third quartiles (*n* represents the number of mitochondria analysed and is stated in the panel) analysed using two-tailed Student’s *t*-test. **h**,**i**, Immunoblot analysis of BN-PAGE-separated mtETC complexes of mitochondria isolated from control and *Mof* iKO MEFs for assemblies containing complex I (CI) to complex V (CV) (**h**) and from control, *Mof* and *Kansl2* iKO MEFs for assemblies containing complex IV (CIV) (**i**). Representative protein for each complex is indicated on the panel. Complex V served as a loading control. **j**, Schematic representation for galactose conditioning and adaptation (top), doubling time (middle) and OCR (bottom) of control and *Mof* iKO MEFs (mean ± s.e.m., *n* = 3 embryos, two-tailed Student’s *t*-test for doubling time and one-way analysis of variance followed by Tukey multiple comparisons test for OCR). Source data

**Extended Data Fig. 1:**
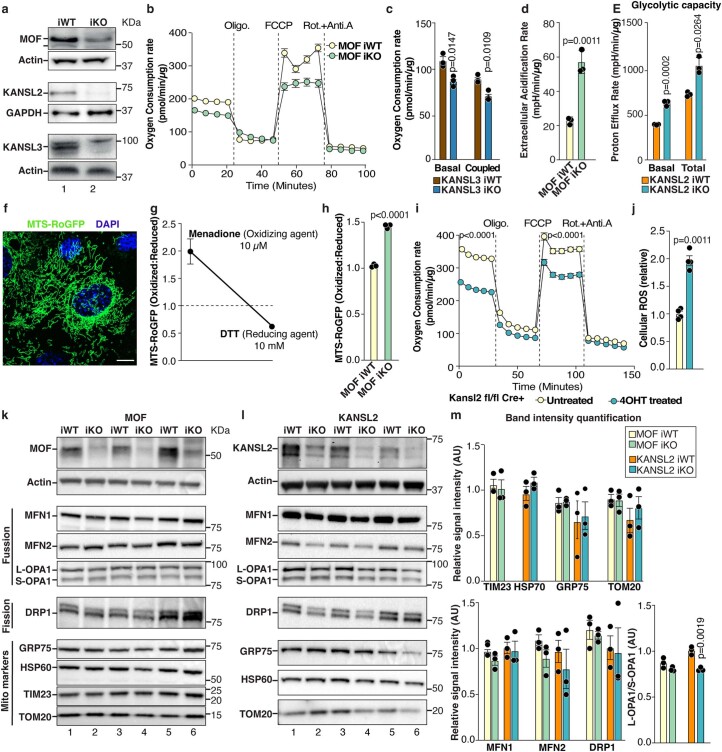
Phenotypic characterization of mitochondrial function, structure and membrane lipid composition. **a**, Verification of KO efficiency using cell extracts of control, *Mof*, *Kansl2* and *Kansl3* iKO MEFs. Actin and GAPDH served as loading controls. **b**, OCR in control and *Mof* iKO MEFs (mean ± s.e.m., n = 3 embryos, see Fig. 1B for statistical test). **c**, OCR in control and *Kansl3* iKO MEFs under indicated states of mitochondrial respiration (mean ± s.e.m., n = 3 embryos, two-tailed Student’s t-test). **d, e**, Extracellular acidification rate (ECAR) in control and *Mof* iKO MEFs induced by glycolysis **(d)** and proton efflux rate (PER) in control and *Kansl2* iKO MEFs induced by basal glycolysis and total glyclotic capacity **(e)** (mean ± s.e.m., n = 3 embryos, two-tailed Student’s t-test). **f**, Live imaging of MEFs expressing MTS-roGFP. **g**, Standardization of ratiometric analysis of MTS-RoGFP in WT MEFs using menadione as an oxidizing agent and DTT as a reducing agent (mean ± s.d., n = 2 embryos). DTT, Dithiothretol. **h**, Quantification of mitochondrial ROS in control and *Mof* iKO MEFs using MTS-roGFP (mean ± s.e.m., n = 3 embryos, two-tailed Student’s t-test). **i**, OCR in *Kansl2*^fl/fl^ *Cre-ERT2*, untreated (control) or 4-OHT-treated (*Kansl2* iKO) MEFs (two-tailed Student’s t-test, statistical analysis was performed on the first time point of basal respiration and on the first time point of respiration following FCCP injection). **j**, Flow cytometry of DHE in *Kansl2*^fl/fl^ *Cre-ERT2*, untreated (control) or 4-OHT treated (*Kansl2* iKO) MEFs (mean ± s.e.m., n = 4 embryos, two-tailed Student’s t-test). **k-m**, Immunoblot on whole cell lysates of control and *Mof* **(k)** or *Kansl2* **(l)** iKO MEFs (n = 3 independent biological replicates). Actin was used as a loading control. GRP75 and HSP60 (mitochondrial matrix), TIM23 (IMM) and TOM20 (OMM) were used as markers and normalized against actin for quantification of mitochondrial mass **(m, upper)**. Components of the mitochondrial fission (DRP1) and fusion (MFN1, MFN2, OPA1) machineries were detected and quantified by normalization against TIM23 for *Mof* iWT and *Mof* iKO, and HSP60 for *Kansl2* iWT and *Kansl2* iKO. OPA1 was quantified as a ratio between its long and short isoform (mean ± s.e.m., n = 3 embryos). Source data

To characterize the function of the MOF acetyltransferase in maintaining mitochondrial homeostasis, we employed primary *Cre-ERT2*^*+*^ mouse embryonic fibroblasts (MEFs) isolated from E13.5 embryos as a model system. Treatment with 4-hydroxy-tamoxifen (4-OHT) for 4 days induced conditional knockout (iKO) of homozygously floxed *Mof* or KANSL complex members, *Kansl2* and *Kansl3* (ref. ^22^). Floxed but *Cre-ERT2*^−^ MEFs obtained from littermates (iWT) served as a control to rule out any side effects of 4-OHT treatment (Fig. 1a and Extended Data Fig. 1a). To evaluate whether the MOF–KANSL complex regulates OXPHOS in aerobically respiring primary cells, we performed in vivo cellular respirometry assays to track mitochondrial energy metabolism in MEFs. Loss of MOF, KANSL2 or KANSL3 led to altered basal, ATP-coupled and maximal respiration, confirming that these proteins play an important role in mitochondrial respiration in primary fibroblasts (Fig. 1b and Extended Data Fig. 1b,c). *Mof* and *Kansl2* iKO MEFs showed concomitant increase in glycolysis as demonstrated by enhanced extracellular acidification rate (ECAR) or proton efflux rate, respectively, indicating a possible compensatory mechanism to maintain cellular energy requirements in these cells (Extended Data Fig. 1d,e).

We observed elevated levels of mitochondrial reactive oxygen species (mtROS) upon loss of MOF, KANSL2 and KANSL3, pointing towards global mitochondrial dysfunction in association with OXPHOS deficiency (Fig. 1c). We verified the mitochondrial ROS accumulation upon MOF depletion using an orthogonal approach where we expressed mitochondrially targeted redox-sensitive green fluorescent protein (roGFP) in MEFs before induction of the knockout (KO)^23,24^. We validated the redox sensor activity of roGFP by treating MEFs with dithiothreitol, a reducing reagent, and menadione, which induces ROS production, thereby leading to oxidation of the mitochondria (Extended Data Fig. 1f–h). We observed similar impaired OXPHOS and increased ROS production upon KANSL2 depletion, using untreated *Kansl2 fl/fl Cre-ERT2* MEFs as the control, thereby ruling out the leaky activity of CRE–ERT2 recombinase (Extended Data Fig. 1i,j). MEFs depleted of MOF and KANSL2 also showed reduced mitochondrial membrane potential per individual cell when stained with tetramethylrhodamine methyl ester (TMRM) (Fig. 1d, left). We also determined the membrane potential of *Mof fl/fl Cre-ERT2* (wild type), 4-OHT-treated *Mof fl/fl* (iWT) and *Mof fl/fl Cre-ERT2* (iKO) MEFs in the presence of oligomycin and carbonyl cyanide-*p*-trifluoro-methoxyphenylhydrazone (FCCP) and could conclude that individual mitochondria per cell show reduced mitochondrial potential differences upon MOF depletion (Fig. 1d, right). The protein levels of mitochondrial markers TIM23, HSP60, GRP70 and TOM20 were unchanged in *Mof* and *Kansl2* iKO MEFs as compared with respective iWT controls (Extended Data Fig. 1k–m).

**Extended Data Fig. 2:**
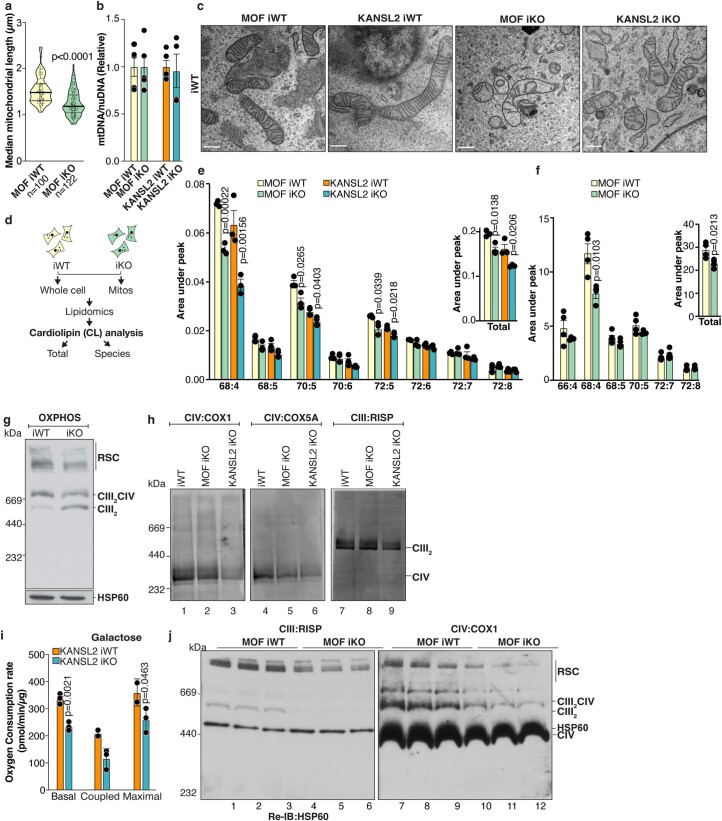
Analyses of mitochondrial proteins and protein complexes. **a**, Quantification of mitochondrial branch length from immunostaining of control and *Mof* iKO MEFs as shown in Fig. 1e using the MiNA FIJI macro (violin plots show all data points with lines at the median and 1st and 3rd quartiles, n=number of mitochondria analysed in 3 embryos, two-tailed Student’s t-test). **b**, mtDNA content measured in control, *Mof* and *Kansl2* iKO MEFs (mean ± s.e.m., normalised to the mean of the corresponding iWT control, n = 4-5 embryos). **c**, Electron micrographs of mitochondria from control, *Mof* and *Kansl2* iKO MEFs. Scale bars: 500 nm. **d**, Schematic outline for the lipidomics experiments. **e, f**, Total (insets) and individual cardiolipin species were quantified using quantile normalization on whole cell **(e)** and enriched mitochondrial extracts **(f)** (mean ± s.e.m., n = 3 embryos, two-tailed Student’s t-test with multiple comparisons). **g**, Mitochondria from control and *Mof* iKO MEF cells were lysed with digitonin and analysed by blue native electrophoresis. Respiratory chain complexes were detected by immunoblotting using the total OXPHOS Rodent WB Antibody cocktail. **h**, Mitochondria from control, *Mof iKO* and *Kansl2* iKO MEF cells were lysed with dodecylmaltoside and analysed by blue native electrophoresis by immunodetection with antisera against complex IV (COX1, COX5A) and complex III (Rieske iron sulfur protein, RISP) of the respiratory chain. **i**, OCR in control and *Kanls2* iKO MEFs adapted in galactose media conditions under different states of mitochondrial respiration (mean ± s.e.m., n = 3 embryos, two-tailed Student’s t-test). **j**, Mitochondria from control and *Mof* iKO MEF cells were lysed with digitonin and analysed by blue native electrophoresis and immunoblotting with the indicated antisera. HSP60 served as a loading control. Source data

Mitochondrial morphology and energy metabolism are suggested to have an interdependent relationship^25^. Therefore, we sought to determine the functional impact of the MOF–KANSL complex on the structural organization of the cellular mitochondrial network. While mitochondria in iWT cells appeared as long interconnected filaments, loss of MOF led to a more fragmented morphological state with reduced branching in accordance with a shorter median mitochondrial length (Extended Data Fig. 2a and Supplementary Movies 1 and 2). Mitochondrial DNA (mtDNA) content (Extended Data Fig. 2b) and abundance of the key proteins responsible for mitochondrial fission (DRP1) and fusion (OPA1, MFN1 and MFN2), showed no substantial difference between the iWT and the iKO MEFs (Extended Data Fig. 1k–m). These findings suggest that the mitochondria lose their structural integrity upon loss of MOF–KANSL complex and it is not an indirect consequence of changes in mtDNA copy number and key molecular effectors of mitochondrial dynamics. Notably, mitochondrial fragmentation in *Mof* iKO cells was accompanied by reduced cristae density, as evident by transmission electron microscopy (TEM) analysis (Fig. 1f,g and Extended Data Fig. 2c). We performed cellular and mitochondrial lipidomics upon *Mof* and *Kansl2* iKO to determine the membrane content of cardiolipins (CL), which are a key factor associated with structural changes of the mitochondrial cristae (Extended Data Fig. 2d–f and Supplementary Table 1)^26^. We hypothesize the mild reduction of some of the CL species observed in *Mof* iKO is a consequence of impaired OXPHOS^27^ and accumulated mitochondrial ROS^28^.

The mitochondrial cristae house the mitochondrial electron transport chain (mtETC) complexes. Therefore, we next focused on the mtETC complexes and their supramolecular assemblies known as respiratory supercomplexes (RSCs). Respirasomes, consisting of CI, CIII and CIV, reside on the cristae of the inner mitochondrial membrane (IMM) and are associated with cristae shape and density^29^. Using digitonin-solubilized mitochondria for blue native polyacrylamide gel electrophoresis (BN-PAGE) analysis, we found that loss of MOF and KANSL2 strongly diminishes RSC levels, with the strongest effects on complex IV (also known as cytochrome c oxidase), while the levels of complexes comprising CII and CV remained mainly unaffected (Fig. 1h,i and Extended Data Fig. 2g). Additionally, we lysed mitochondria isolated from control, *Mof* and *Kansl2* iKO MEFs with dodecylmaltoside to dissociate the supercomplexes and detected reduced levels of complex IV upon MOF and KANSL2 depletion (Extended Data Fig. 2h). Respirasomes are vital to enhance the efficiency of electron transfer required during metabolic or nutrient stress^30–34^. For example, cells deprived of glucose or grown in galactose activate their mitochondrial respiration to cope with the energy demand and maintain survival and growth^32,35^. To investigate the functional relevance of the respirasome destabilization upon depletion of MOF, we replaced glucose with galactose as the carbon source in the cellular growth medium (Fig. 1j, top). A minimum of 4 days of conditioning was sufficient to switch media dependency of the MEFs from glucose to galactose, as verified by lengthening of the doubling time (Fig. 1j, middle). Conditioning the MEFs in the galactose medium failed to ramp up mitochondrial respiration upon MOF and KANSL2 depletion (Fig. 1j, bottom and Extended Data Fig. 2i), indicating the inability to form and utilize the respirasomes in the absence of the MOF–KANSL complex. Additionally, we observed severe respirasome destabilization in *Mof* iKO MEFs grown in galactose media conditions (Extended Data Fig. 2j). We conclude that the MOF–KANSL complex is a key player in the regulation of mitochondrial structure, mtETC assembly and function in primary MEFs.

### Mitochondrial defects upon MOF loss are independent of its role in transcriptional regulation

**Extended Data Fig. 3:**
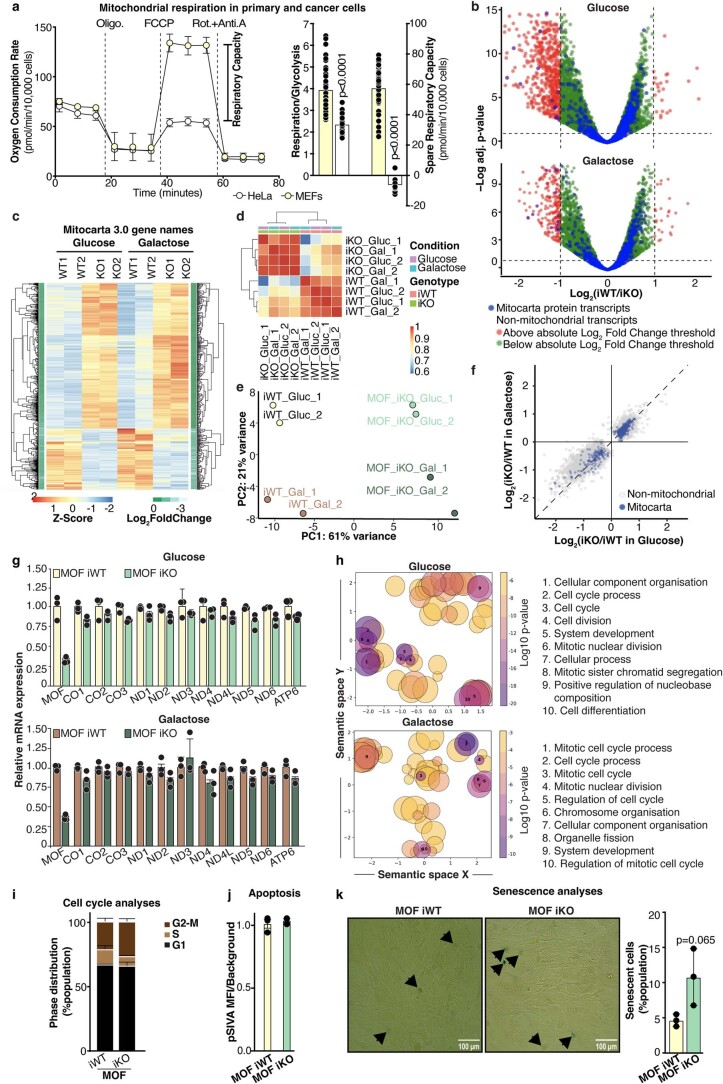
Discrete effects of MOF depletion on transcriptional regulation and mitochondrial health. **a**, OCR **(left panel)**, ratio of OCR to ECAR **(right panel, left axis)**, and spare respiratory capacity **(right panel, right axis)** in HeLa and primary MEFs (mean ± s.e.m., n = 45 independent samples, two-tailed Student’s t-test). **b**, RNA-seq analysis of control and *Mof* iKO MEFs adapted to glucose **(left)** or galactose **(right)** growth conditions (n = 2 embryos). Volcano plots show all detected transcripts, with horizontal dashed line indicating the Padj<0.05 significance cutoff (calculated by Wald Tests) and vertical dashed line indicating the Log_2_ FC cutoff analysed by DESeq2. **c**, Heatmap shows Z-score expression of all detected transcripts belonging to mouse Mitocarta 3.0 genes. **d, e** Heatmap (**d**) and PCA plot (**e**) showing sample-to-sample distances of RNA-seq samples categorized according to their genotype and medium condition. **f**, Scatter plot of transcripts from (**b**) depicts correlation of transcriptional changes (Log_2_ FC) upon *Mof* iKO in glucose and galactose growth conditions, with MitoCarta 3.0 protein transcripts highlighted in blue. **g**, Relative expression of OXPHOS transcripts encoded by mtDNA in control and *Mof* iKO MEFs adapted to glucose **(upper)** or galactose **(lower)** growth conditions (mean ± s.e.m., normalised to ribosomal *Rlpl0* and shown as fold change over iWT, n = 3 embryos). **h**, Bubble plots show summarised biological processes affected in *Mof* iKO MEFs adapted in glucose **(upper)** or galactose **(lower)** growth conditions. Size of bubbles depicts the number of terms belonging to the summary term whereas the colour shows their Log_10_ p-value. The position of the term signifies their relation in semantic space. **i, j**, Flow cytometry of control and *Mof* iKO MEFs for distribution of cells in the indicated cell cycle phase by staining with propidium iodide **(i)** and analysis of apoptosis by quantification of exposed annexin V using pSIVA dye **(j)** (mean ± s.e.m., normalized to the mean of the iWT, n = 3 embryos, two-tailed Student’s t-test with multiple comparison and Holm-Sidak method). **K**, Representative images **(left)** and corresponding quantification **(right)** of senescence-associated b-galactosidase activity in control and *Mof* iKO MEFs. Senescent cells are indicated with black arrows (mean ± s.e.m., n = 3 embryos, two-tailed Student’s t-test). Source data

MOF and its KANSL complex partner, KANSL1, were previously shown to be required for transcriptional control of the mitochondrial genome-encoded OXPHOS system subunits in HeLa cells grown in galactose media conditions^10^. As opposed to HeLa cells, which exhibit Warburg effect (anaerobic respiration), MEFs are primary cells and exhibit enhanced dependency on mitochondrial respiration, making these two cell types discrete in terms of their metabolic status. While MEFs harbour healthy mitochondria as they rely more on OXPHOS, HeLa cells have poor mitochondrial quality and rely less on OXPHOS (Extended Data Fig. 3a).

Given the role of the MOF–KANSL complex in transcription regulation^10,36^, we performed a total RNA sequencing (RNA-seq) experiment to investigate transcriptomic changes that could be underlying the mitochondrial dysfunction. To this end, we induced *Mof* iKO using 4-OHT in primary MEFs that were adapted either to glucose or galactose growth media. Overall, 646 downregulated and 28 upregulated genes (adjusted *P* value <0.05 and absolute log_2_ fold change >1) were identified under glucose growth conditions (Extended Data Fig. 3b and Supplementary Table 3). Twenty-one of the downregulated genes code for mitochondrial proteins (Extended Data Fig. 3b and Supplementary Table 2). The majority of transcripts that encode mouse MitoCarta 3.0 proteins were slightly upregulated upon loss of MOF. We have detected around 90 transcripts, common to both glucose and galactose conditions, which showed a mild downregulation (−1 < log_2_ fold change < 0) (Extended Data Fig. 3c and Supplementary Table 4). Intersample correlation as shown by heat map and principal component analysis (PCA) plot indicated that KO of *Mof* has similar effects on cells irrespective of whether they are grown in glucose and galactose media (Extended Data Fig. 3d,e). In general, the transcriptional downregulation in the galactose medium (down: 332 genes, up: 31 genes with adjusted *P* value <0.05 and absolute log_2_ fold change >1) was less pronounced than in the glucose medium (Extended Data Fig. 3b and Supplementary Table 5). Furthermore, contrary to cancer cells, there were no genes encoding mitochondrial proteins that showed differential dysregulation upon loss of MOF in a growth media-dependent manner in primary cells (Extended Data Fig. 3f)^10^. We performed quantitative reverse transcription polymerase chain reaction (qRT–PCR) for the OXPHOS messenger RNAs encoded by the mtDNA to validate the RNA-seq results and could not observe any major changes in the levels of these transcripts between *Mof* iWT and iKO MEFs in neither glucose nor galactose medium (Extended Data Fig. 3g).

Gene Ontology term analysis of dysregulated genes indicated a potential role of MOF in regulating the cell cycle, which has been also previously described (Extended Data Fig. 3h)^37^. To rule out the changes in cell cycle progression, apoptosis and cellular senescence that could possibly explain the dynamic changes of mitochondrial network, we performed propidium iodide, annexin V staining and β-galactosidase activity measurement, respectively^38–40^. We observed no major changes in cell cycle progression, apoptosis rate or in cellular senescence in our cells upon induction of *Mof* iKO for 4 days (Extended Data Fig. 3i–k).

**Fig. 2:**
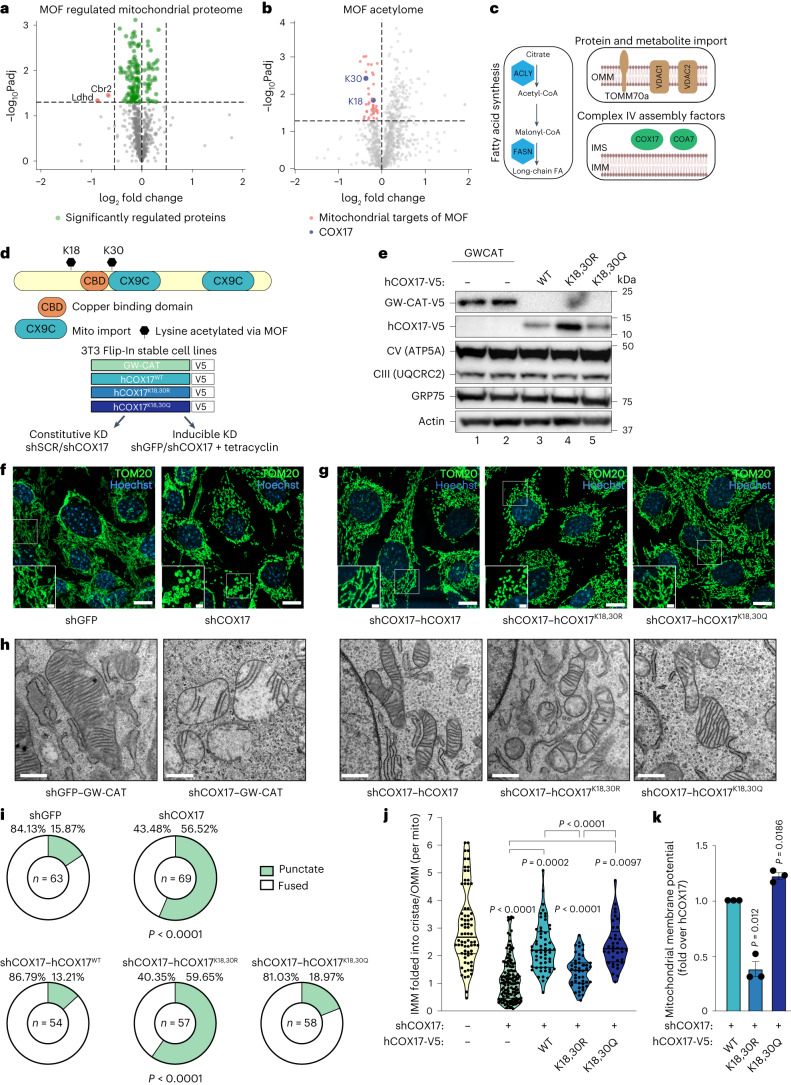
COX17 acetylation maintains mitochondrial morphology and cristae ultrastructure. **a**,**b**, SILAC proteomics on isolated mitochondria (**a**) and acetyl proteomics representing distribution of acetyl (lysine) sites on mitochondrial proteins from ref. ^22^ (**b**) following *Mof* iKO in MEFs (*n* = 6 independent biological replicates). Volcano plot shows all detected mouse Mitocarta 3.0 proteins (**a**) and acetylation sites (**b**). Horizontal dashed line indicates the *P* < 0.05 significance cut-off (two-sided Student’s *t*-test), and vertical dashed line indicates the log_2_ fold change cut-off, wherever applicable. The significantly affected COX17 acetylation sites are highlighted in blue. **c**, Scheme of mitochondrial acetylation targets of MOF that are acetylated at multiple lysine residues. **d**, Top: mouse COX17 lysine sites acetylated via MOF. Bottom: Flp-In-3T3 fibroblast cell lines expressing wild type, acetylation mimic and non-acetylated version of human COX17 (hCOX17) with simultaneous KD of endogenous mouse *Cox17* by shRNA. A cell line expressing chloramphenicol acetyltransferase (GW-CAT) was used as a control. All transgenes are C-terminal V5-tagged and stably expressed from the single FRT locus. **e**, Immunoblot of cell lines in **d** using anti-V5 antibody and mtETC complexes using the Total OXPHOS Rodent WB Antibody cocktail. GRP75 and β-actin were used as loading controls. **f**,**g**, Immunofluorescence microscopy images of tetracycline-induced control (shGFP) and *Cox17* KD (shCOX17#1) 3T3 fibroblasts (**f**) and indicated hCOX17 variant cell lines with constitutive KD of endogenous *Cox17* (**g**) stained for TOM20. Scale bars, 20 µm (insets 2 µm). **h**, Electron micrographs of mitochondria from control and indicated hCOX17 variant 3T3 fibroblasts with constitutive KD of endogenous *Cox17*. Scale bars, 500 nm. **i**, Distribution of cells in **f** and **g** with fused (white) or punctate (green) mitochondria. *n*, number of cells analysed (part of the whole, chi-square tests). **j**, Cristae density in COX17 variants in **h** analysed as in Fig. 1f,g. Violin plots show all data points with lines at the median and first and third quartiles (one-way analysis of variance followed by Tukey multiple comparisons test). **k**, Flow cytometry of TMRM in cells expressing the indicated hCOX17 variants (*n* = 3 independent experiments, mean ± s.e.m., normalized to the mean of the indicated control, two-tailed one-sample *t*-test and Wilcoxon test). Source data

**Extended Data Fig. 4:**
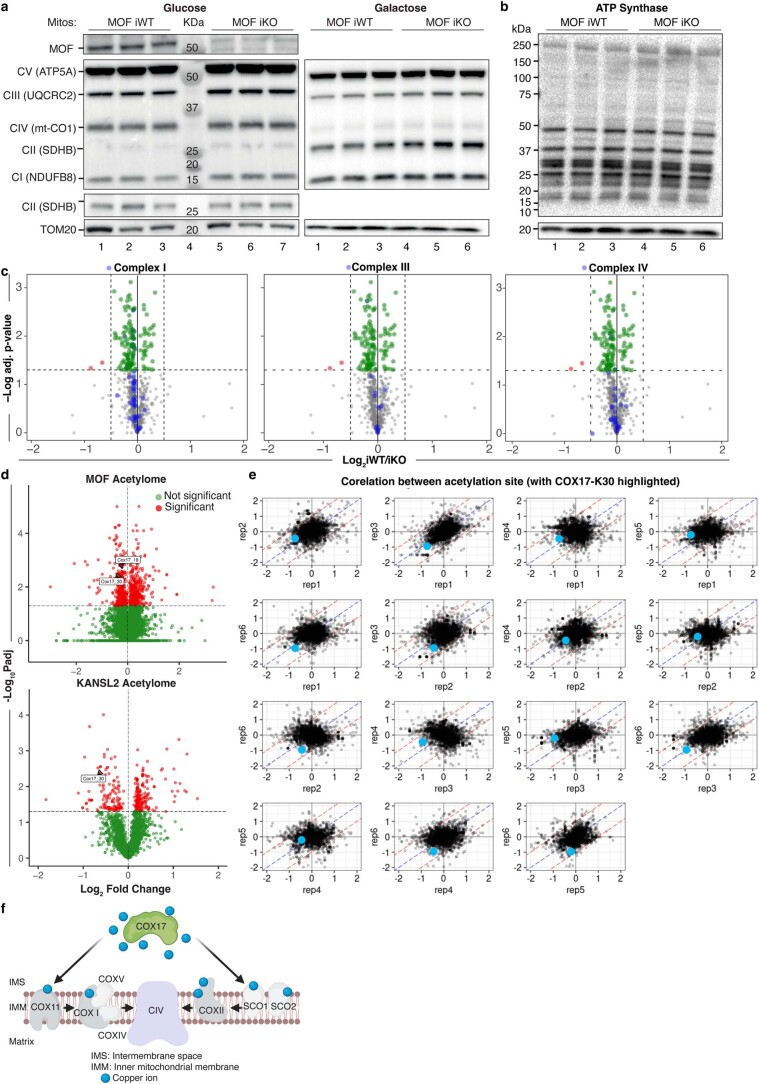
Proteomics and acetyl-proteomics upon MOF and KANSL2 depletion. **a**, Immunoblot analysis of components of mtETC using the Total OXPHOS Rodent WB Antibody cocktail in mitochondrial lysates of control and *Mof* iKO MEFs adapted to glucose **(left)** or galactose **(right)** growth conditions in 3 independent embryos. TOM20 served as a loading control. **b**, Immunoblot of ATP-Synthase subunits using Rodent WB Antibody cocktail in mitochondrial lysates of control and *Mof* iKO MEFs in 3 independent embryos. TOM20 served as a loading control. **c**, Mitochondrial proteomics upon MOF iKO. Volcano plots show all detected mouse Mitocarta 3.0 proteins with horizontal dashed line indicating the P < 0.05 significance cutoff (calculated by Wald Tests) and vertical dashed line, wherever applicable, indicating the log2 fold change cutoff. Components of mtETC complexes I, II and IV are highlighted in blue. Annotation for complex I, III and IV proteins is adapted from mouse Mitocarta 3.0. **d**, Reanalysis of the distribution of acetyl (lysine) sites following *Kansl2* iKO in MEFs from Karoutas *et al*. (2019) (n = 6 independent biological replicates). Volcano plot shows all detected acetylation sites on mouse Mitocarta 3.0 proteins. Horizontal dashed line indicates the P < 0.05 significance cutoff (one-sided student t-test). The significantly affected COX17 acetylation sites are highlighted in blue. **e**, Correlation between replicates for acetyl-proteomics upon KANSL2 depletion, highlighting (in blue) COX17 in the third quadrant of all the plots. **f**, Scheme for the placement of COX17 as a copper chaperone in the functional assembly of mitochondrial complex IV.

We further performed quantitative stable isotope labelling by amino acids in cell culture (SILAC)-based mitochondrial proteome analysis to investigate protein abundances upon MOF depletion (Supplementary Table 6). We could rule out a global proteome change that could possibly explain the mitochondrial phenotype in *Mof* iKO MEFs (Fig. 2a). Moreover, the only two proteins, LDHD and CBR2, with a reduced abundance (adjusted *P* value <0.05 and log_2_ fold change <−0.5; *n* = 6 independent biological replicates) upon loss of MOF relative to iWT, are not directly related to the observed defects in the mtETC^41^. Additionally, western blot analysis verified that the levels of representative proteins of the OXPHOS machinery remained unaffected upon *Mof* iKO in glucose or galactose conditions (Extended Data Fig. 4a,b). Moreover, individual member proteins of complex I, III and IV of the mtETC also showed overall no significant change in their abundance (Extended Data Fig. 4c). Considering the lack of major transcriptional and translational defects which could explain mitochondrial dysfunction elicited by *Mof* iKO, we hypothesized that altered post-translational acetylation patterns of mitochondrial proteins upon loss of MOF may drive the observed phenotype.

### Loss of COX17 acetylation disrupts mitochondrial morphology and ultrastructure

Undertaking a MOF- and KANSL2-specific global acetylome analysis, we had previously reported multiple non-histone targets that reside in different subcellular compartments. A closer look at this published dataset revealed several mitochondrial proteins that showed reduced acetylation at multiple lysine residues upon loss of MOF (Fig. 2b, Extended Data Fig. 4d and Supplementary Table 7)^22^. These proteins majorly belong to pathways such as protein and metabolite import, mtETC complex IV assembly and long-chain fatty acid synthesis (Fig. 2c). We observed the most severe effect on complex IV assembly upon MOF or KANSL2 loss (Fig. 1h,i). Therefore, COX17 was of particular interest among the mitochondrial acetylation targets of MOF because of its role in the functional assembly of cytochrome c oxidase and also in the formation of RSCs^42,43^. Additionally, COX17 was also identified as a mitochondrial target of KANSL2 (Extended Data Fig. 4e) and has not been so far detected as a target of other promiscuous acetyltransferases such as CBP/p300 (ref. ^44^). Moreover, MOF and KANSL3 were shown to localize in the mitochondrial intermembrane space (IMS) and matrix, making them possible to directly acetylate COX17 in the IMS^10^. COX17 is a soluble IMS metal chaperone that initiates a copper relay that is crucial for copper delivery to COX1 and COX2 modules, which form a functional complex IV (Extended Data Fig. 4f)^45,46^.

**Extended Data Fig. 5:**
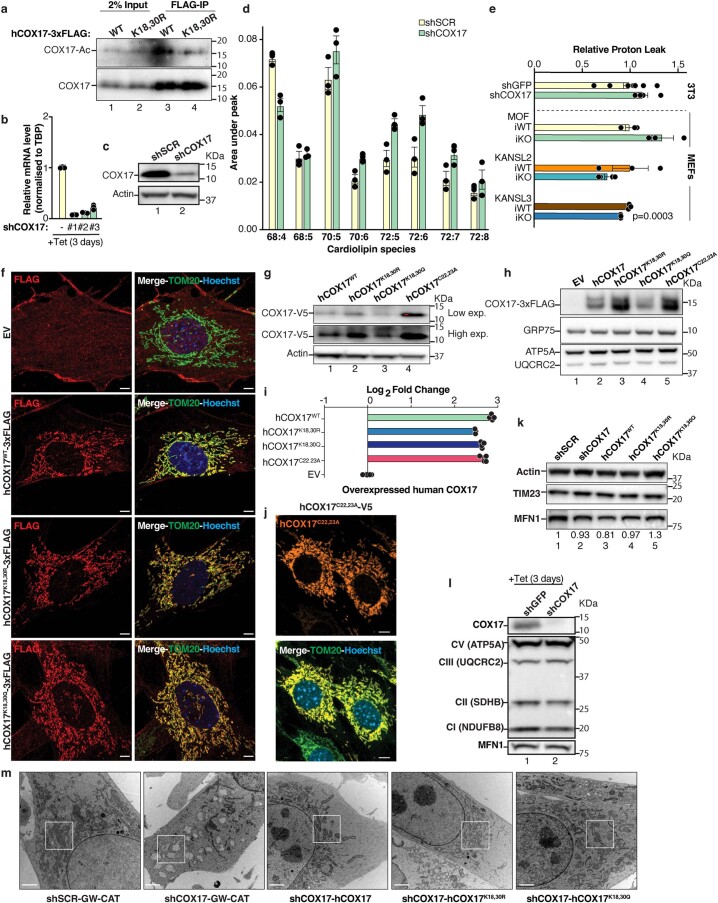
Characterisation of COX17 variants. **a**, Immunoblot analysis of acetylation level of immunoprecipitated wildtype COX17 or its non-acetylatable mimic (COX17-K18,30 R) overexpressed in MEFs. **b**, Relative expression of *Cox17* by qRT-PCR analysis for verification of tetracyclin (Tet) induced knockdown for 3 days using 3 independent inducible shRNAs (#1, #2, #3) against *Cox17* in MEFs. Data indicate mean ± s.e.m. and is normalised to *Tbp* and shSCR control, n = 3 embryos. **c**, Immunoblot of cell lysates for verification of efficiency of constitutive *Cox17* knockdown (KD) in MEFs. shSCR served as a control. Actin served as a loading control. **d**, Analysis of individual cardiolipin species in control and COX17 KD MEFs as quantified using quantile normalization. Data indicate mean ± s.e.m. n = 3 embryos. **e**, Relative proton leak in indicated controls, COX17 KD 3T3 fibroblasts, *Mof*, *Kansl2* and *Kansl3* iKO MEFs. Data indicate mean ± s.e.m. and is normalized to the mean of the corresponding iWT, n = 3-7 embryos analysed using two-tailed Student’s t-test. **f**, Immunofluorescence microscopy images of hCOX17 variants in MEFs using antibodies against FLAG (red), mitochondrial TOM20 (green) and nuclear Hoechst (blue). Scale bars: 10 µm. **g, h**, Immunoblot analysis for ectopic expression of human COX17 variants in 3T3 fibroblasts using anti-V5 antibody **(g)** and in MEFs using anti-FLAG antibody **(h)**. Actin, GRP75, ATP5A and UQCRC2 were used as a loading control. **i**, Log_2_ Fold Change of mRNA expression of ectopic human COX17 variants as analysed by qRT-PCR. All COX17 variants are C-terminal FLAG-tagged and expressed stably in MEFs using lentivirus. Data indicate mean ± s.e.m. normalised to *Hsp90*, n = 3-4 independent experiments. **j**, Immunofluorescence microscopy images of hCOX17 variant in 3T3 fibroblasts using antibodies against COX17 (red), mitochondrial TOM20 (green) and nuclear Hoechst (blue). Scale bars: 10 µm. **k**, Immunoblot analysis of cell lysate of indicated hCOX17 variant 3T3 fibroblast line for mitochondrial content using antibody against TIM23 and for fusion protein MFN1. MFN1 band intensities, normalized to TIM23, are indicated in the panel. **l**, Immunoblot analysis of components of mtETC using the Total OXPHOS Rodent WB Antibody cocktail in mitochondrial lysates upon COX17 knockdown induction using shCOX17#1 for 3 days in MEFs. MFN1 served as a loading control. **m**, Low magnification electron micrograph of a single cell from the indicated hCOX17 variant cell lines, depleted of endogenous *Cox17*. White boxes indicate the region of interest as shown in Fig. 2j. Scale bars: 2 μm. Source data

We identified two sites of COX17, K18 and K30 (adjusted *P* value <0.01), as acetylation targets of the MOF–KANSL complex (Fig. 2b and Extended Data Fig. 4d,e). While COX17–K18 maps on its disordered N-terminal region, COX17–K30 is located within the first of the twin-CX9C motif and exposed at the surface of the protein (Fig. 2d). We overexpressed the non-acetylated mimetic of COX17, COX17–K18,30R, verified that it leads to reduced overall acetylation of COX17 (Extended Data Fig. 5a) and hence hypothesized that a combined reduced acetylation of these two strategically located lysine residues might have a functional consequence in the energy metabolism in MOF-depleted cells. To study the influence of acetylation of these two COX17 lysine residues combined on its function, we generated mouse Flp-In 3T3 fibroblast cell lines that stably express single copies of human hCOX17 derivatives, wild type (hCOX17^WT^), acetylation mimicking (hCOX17^K18,30Q^) or non-acetylated (hCOX17^K18,30R^) variants. We simultaneously depleted endogenous *Cox17* using constitutive or inducible short hairpin RNA (shRNA) to enable observation of full impact of the COX17 acetylation mutants on mitochondrial structure and function (Fig. 2d and Extended Data Fig. 5b,c). CL species are only mildly regulated upon COX17 knockdown (KD) (Extended Data Fig. 5d and Supplementary Table 1), similar to observations in MOF and KANSL2 KO cells (Extended Data Fig. 1n). Additionally, proton leak calculated from Seahorse in vivo cellular respirometry assays upon COX17 KD and MOF–KANSL complex KO showed no global significant difference, ruling out the alternative hypothesis of proton leak generated by altered CL content destabilizing the formation of the respiratory complexes (Extended Data Fig. 5e).

**Fig. 3:**
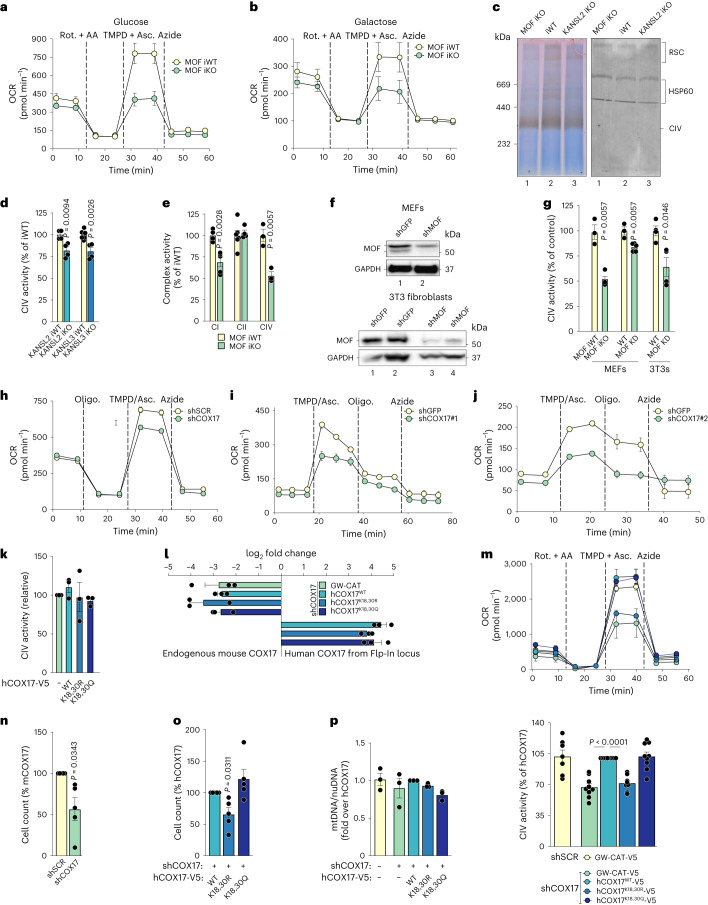
Loss of the MOF–KANSL complex and COX17 acetylation impair mitochondrial cytochrome c oxidase activity. **a**,**b**, OCR in permeabilized control and *Mof* iKO MEFs, adapted to glucose (**a**) or galactose (**b**) growth conditions (mean ± s.e.m., *n* = 3–5 embryos; for statistical test, see **g**). Rot, rotenone; AA, antimycin A; Asc, ascorbate. **c,** In-gel activity staining for non-denaturing native PAGE-separated cytochrome c oxidase containing complexes of mitochondria isolated from control, *Mof* and *Kansl2* iKO MEFs. HSP60 immunodetection served as a loading control. **d**, Complex IV (CIV) activity in control, *Kansl2* and *Kansl3* iKO MEFs (*n* = 4–6 embryos, mean ± s.e.m., normalized to the mean of the corresponding iWT control, two-tailed Student’s *t*-test). **e**, Analyses of complex I, complex II and complex IV activity in control and *Mof* iKO MEFs (*n* = 3–6 embryos, mean ± s.e.m., normalized to the mean of the corresponding iWT control, two-tailed Student’s *t*-test). **f**, Verification of MOF KD or KO efficiency in MEFs and 3T3 fibroblasts. β-Actin and GAPDH served as loading controls. **g**, Complex IV activity in the indicated cell type under control and MOF depletion conditions (*n* = 3–5 embryos for MEFs and *n* = 4–5 independent experiments for 3T3s, mean ± s.e.m., normalized to the mean of the corresponding WT or iWT control, two-tailed Student’s *t*-test. **h**–**j**, OCR with sequential indicated treatments in control and COX17 KD MEFs using constitutive shRNA (**h**), inducible shRNA #1 (**i**) and inducible shRNA #2 (**j**) (mean ± s.e.m). Oligo, oligomycin. **k**, Complex IV activity in the indicated hCOX17 variant 3T3 fibroblasts without depletion of endogenous COX17 (mean ± s.e.m., normalized to GW-CAT for each experimental replicate, *n* = 3 independent experiments, two-tailed one-sample *t*-test and Wilcoxon test. **l**, qRT–PCR for endogenous mouse *Cox17* (log_2_ fold change (FC) over shSCR–GW-CAT control) and ectopically expressed human COX17 variants (log_2_ FC over shSCR–GW-CAT) in 3T3 fibroblast lines described in Fig. 2d (mean ± s.e.m., normalized to *Tbp*, *n* = 3 independent experiments). **m**, OCR (top) and complex IV activity (bottom) in control and indicated hCOX17 variant fibroblasts (mean ± s.e.m., *n* = 6–9 independent experiments, normalized to hCOX17^WT^ for corresponding experimental replicate, two-tailed one-sample *t*-test and Wilcoxon test). **n**,**o**, Growth analysis of control and COX17 KD (**n**) and indicated hCOX17 variant 3T3 fibroblasts (**o**). Cell count is plotted as percentage of control or hCOX17^WT^ line (mean ± s.e.m., normalized to the indicated control for corresponding experimental replicate, *n* = 5 independent experiments, two-tailed one-sample *t*-test and Wilcoxon test). **p**, mtDNA content measured in the indicated 3T3 fibroblast line using primers against mtDNA *mt-Co1*, normalized to nuclear DNA using primers against *Ndufv1* (mean ± s.e.m., *n* = 3 independent experiments, normalized to the indicated control for corresponding experimental replicate). Source data

We also expressed the corresponding hCOX17 variants in primary MEFs using lentivirus transduction (Extended Data Fig. 5f). All the variants of human COX17 localized to the mitochondria; however, hCOX17^K18,30R^ protein levels were higher relative to hCOX17^WT^ and hCOX17^K18,30Q^ in 3T3 fibroblasts as well as MEFs, despite comparable steady-state RNA levels (Figs. 2e and 3l, and Extended Data Fig. 5g–i). We also stably expressed human COX17 that is unable to deliver copper to the downstream proteins, hCOX17^C22,23A^, thereby serving as a negative control^47^. Intriguingly similar to hCOX17^K18,30R^, while still localizing to the mitochondria, hCOX17^C22,23A curiosly^ accumulated in its protein levels, which had not been demonstrated in earlier studies (Extended Data Fig. 5g–j)^47,48^. Taken together, these data rule out destabilization as an alternative hypothesis behind loss of function of the non-acetylated form of COX17 and additionally indicate a mechanism of aberrant accumulation that operates post-mitochondrial import, possibly due to slower turnover of inactive forms of the COX17. However, representative components from complex I, II, III and V, as well as fusion protein MFN1, remained unchanged in COX17 KD MEFs and in the hCOX17 variant 3T3 fibroblasts (Fig. 2e and Extended Data Fig. 5k,l).

We show that KD of *Cox17* led to a more fragmented mitochondrial morphology, which is similar to depletion of the MOF–KANSL complex (Figs. 1e and 2f). Mitochondrial fragmentation inflicted by Cox17 KD could be rescued by hCOX17^WT^ and hCOX17^K18,30Q^, but not by hCOX17^K18,30R^ (Fig. 2g,i). COX17 is a newly identified direct interaction partner of the MICOS complex in yeast that is crucial for the establishment and maintenance of IMM architecture, but the effect of COX17 loss on cristae ultrastructure has not been studied so far^49^. TEM analysis of mitochondria from the stable cell lines showed that loss of COX17 results in reduced cristae density that could be rescued by hCOX17^WT^ and hCOX17^K18,30Q^ but not by the non-acetylated hCOX17^K18,30R^ (Fig. 2h,j and Extended Data Fig. 5m). In accordance with these results, hCOX17^K18,30R^ exhibited reduced membrane potential as compared with hCOX17^WT^ or hCOX17^K18,30Q^, further emphasizing mitochondrial dysfunction upon loss of COX17 acetylation (Fig. 2k). In summary, loss of MOF–KANSL complex members strikingly phenocopies the loss of COX17 acetylation in multiple aspects of mitochondrial morphology and cristae ultrastructure.

### MOF–KANSL complex and acetylated COX17 govern complex IV integrity

**Extended Data Fig. 6:**
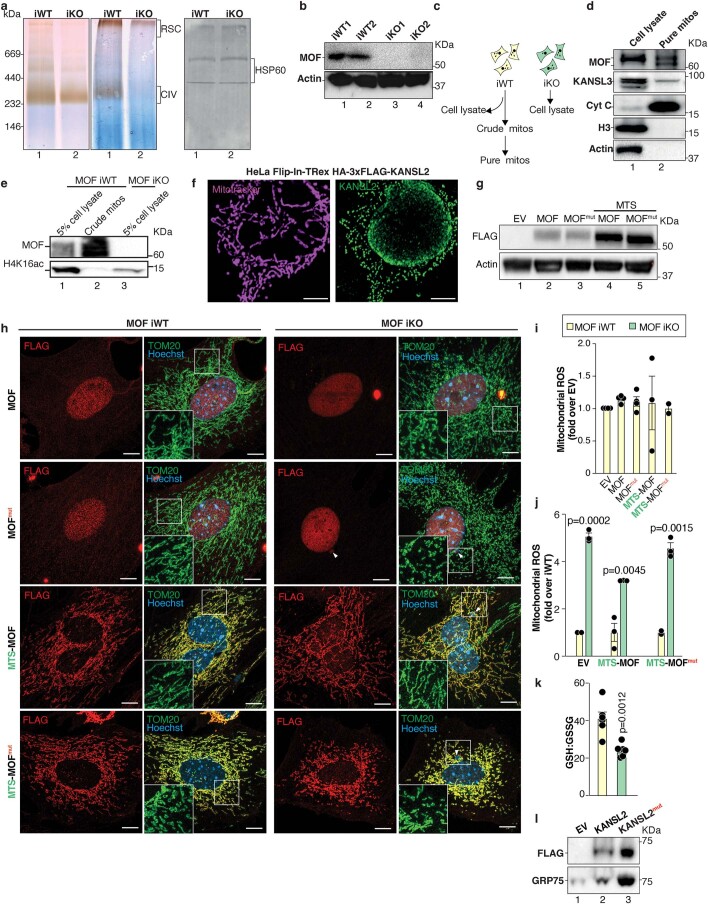
Characterisation of MOF variants. **a**, Mitochondria from control (iWT) and *Mof* iKO MEF cells were lysed with digitonin. Protein complexes were subjected to blue native electrophoresis and in-gel activity stain for complex IV. HSP60 immunodetection served as a loading control. **b**, Immunoblot of cell extracts of control and *Mof* iKO MEFs to verify the corresponding knockout (KO) efficiency by 2 days of 4-OHT induction. Actin and GAPDH served as loading controls. **c**, Experimental scheme for subcellular fractionation in MEFs. **d**, Immunoblot analysis of cell lysate as input and Percoll gradient purified mitochondria for MOF and KANSL3 detection. Quality of fractionation was verified using mitochondrial marker (cytochrome c), nuclear marker (H3) and cytosolic marker (actin). **e**, Immunoblot for detection of enrichment of MOF in the mitochondria. Cell lysate served as the input for crude mitochondrial fraction. Quality of enrichment was verified using H4K16ac as a nuclear marker. Signal specificity of MOF was verified using *Mof* iKO cell lysate. **f**, Structured Illumination immunofluorescence of C-terminal HA-3xFLAG-tagged KANSL2 expressing from FRT locus in HeLa Flp-In T-Rex cells stained with Mitotracker and FLAG antibody. Scale bars: 5 µm. **g, h**, Immunoblot **(g)** and immunofluorescence of FLAG-tagged MOF for expression of indicated MOF variants in MEFs. Beta-actin served as a loading control. Insets of mitochondria indicate selected regions of interest. White arrowheads indicate micronuclei. Scale bars: 10 µm. **i**, Flow cytometry of mtSOX dye in control MEFs expressing the indicated MOF variants (mean ± s.e.m., normalized to the empty vector (EV) control for each experimental replicate, n = 2-6 embryos). **j**, Flow cytometry of mtSOX dye in control and *Mof* iKO MEFs expressing indicated MOF variants (mean ± s.e.m., normalized to the corresponding iWT control for each experimental replicate, n = 2-3 embryos, two-tailed Student’s t-test). **k**, Luminiscent analysis of reduced (GSH) and oxidized (GSSG) glutathione as a measure of anti-oxidant metabolism in control and *Mof* iKO MEFs. Data is represented as a ratio of GSH:GSSH (mean ± s.e.m. n = 5-6 embryos, two-tailed Student’s t-test). **l**, anti-FLAG antibody immunoblot of cell lysates of indicated KANSL2 variants in MEFs. GRP75 served as a loading control. Source data

Since COX17 plays a fundamental role in the functional assembly of complex IV in yeast and in mammals, we analysed complex IV activity upon loss of the MOF–KANSL complex and COX17 using a modified Seahorse assay^42,50^. The oxygen consumption rate (OCR) was measured in permeabilized cells using tetamethylphenylenediamine (TMPD) as an artificial substrate for complex IV upon inhibition of complex I and complex III using rotenone and antimycin A (Fig. 3a). Loss of MOF led to reduced complex IV activity in both glucose and galactose culture media conditions (Fig. 3a,b). In addition, activity of complex IV monomer and complex IV-containing supercomplexes was reduced upon loss of MOF and KANSL2 using the qualitative in-gel activity staining (Fig. 3c and Extended Data Fig. 6a). The regulation of complex IV by MOF as a part of the MOF–KANSL complex was further confirmed by observing complex IV dysfunction in *Kansl2* and *Kansl3* iKO MEFs (Fig. 3d). Since respirasomes are known to be composed of complex I, III and IV and are typically devoid of complex II, we performed activity assays for the different complexes on permeabilized cells^50,51^. While the activity of complex IV and the integrated activity of complex I + III + IV were reduced, the integrated activity of complex II + III + IV remained unaffected, suggesting that reduced respirasome formation by mtETC complexes upon loss of MOF diminishes their activity (Fig. 3e). The phenotype of complex IV defect was conserved across multiple fibroblast cell lines, under different KD and KO strategies (Fig. 3f,g). We verified impaired complex IV activity upon constitutive as well as inducible KD of *Cox17* (Fig. 3h–j). In the presence of endogenous COX17, neither wild type nor COX17 mutants led to any significant change in complex IV activity, thereby eliminating dominant-negative phenotype or a chronic effect resulting from constitutive expression of the COX17 variants (Fig. 3k). KD of *Cox17* in 3T3 fibroblasts severely reduced complex IV activity, which could be rescued by expression of hCOX17^WT^ and hCOX17^K18,30Q^, but not by hCOX17^K18,30R^ (Fig. 3l,m). Similar to lack of respiratory growth in COX17 null yeast strains, loss of endogenous COX17 in 3T3 fibroblasts led to reduced cell proliferation (Fig. 3n)^49^. Expression of hCOX17^K18,30R^, but not hCOX17^WT^ and hCOX17^K18,30Q^, caused cell growth defect, pointing towards the functionality of the COX17 acetylation sites (Fig. 3o). The mtDNA content, however, remained unchanged in all the COX17 variants (Fig. 3p).

### COX17 acetylation via MOF promotes its function

**Fig. 4:**
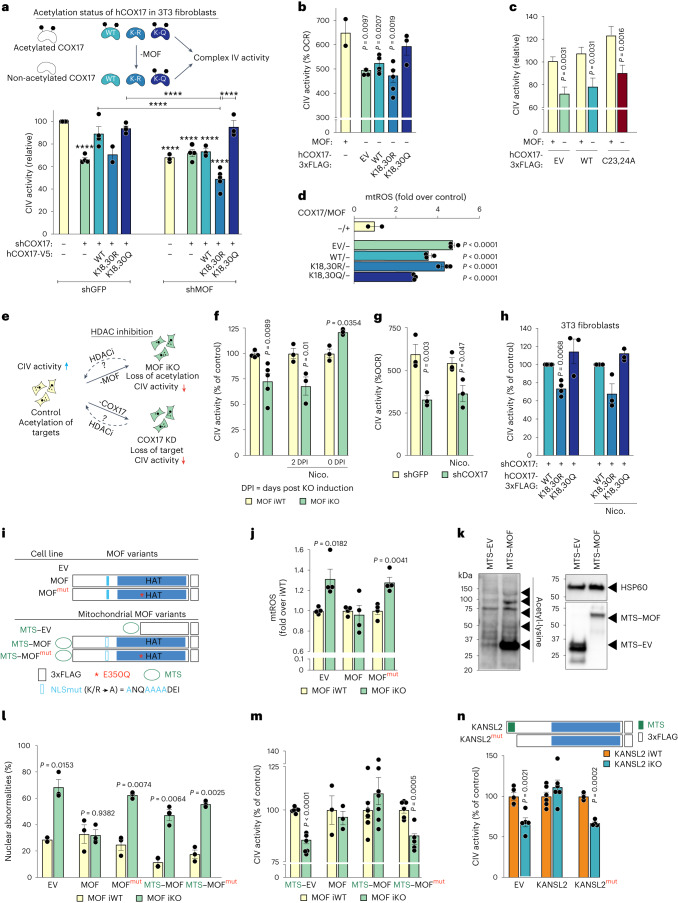
MOF-mediated COX17 acetylation is required for mitochondrial cytochrome c oxidase activity. **a**, Scheme (top) and complex IV (CIV) activity of indicated *Cox17* variant 3T3 fibroblast line upon shRNA-mediated KD of endogenous MOF (bottom); shGFP served as control (mean ± s.e.m., normalized to the indicated control for corresponding experimental replicate, *n* = 2–5 independent experiments; two-way analysis of variance (ANOVA) followed by Šidák’s multiple comparisons test. ****P value <0.0001 are indicated; for exact *P* values, see Supplementary Table 8). **b**,**c**, Complex IV activity in control and *Mof* iKO MEFs transduced with 3xFLAG-tagged lentiviral hCOX17 constructs (mean ± s.e.m., *n* = 2–5 embryos, one-way ANOVA followed by Tukey multiple comparisons test). **d**, Flow cytometry of mtSOX using experimental setup as in **b** (mean ± s.e.m., normalized to the mean of the indicated control, *n* = 2–3 embryos, one-way ANOVA followed by Tukey multiple comparisons test). **e**–**g**, Scheme (**e**) and complex IV activity in control and *Mof* iKO MEFs with or without 10 mM nicotinamide treatment, either in parallel with or 2 days after 4OHT induction **(f**) and in control and *Cox17* KD MEFs with or without 10 mM nicotinamide treatment for 4 days (**g**) (mean ± s.e.m. normalized to the mean of the corresponding iWT control, *n* = 3–5 embryos, two-tailed Student’s *t*-test). **h**, Complex IV activity in indicated hCOX17 variant 3T3 fibroblasts depleted of endogenous *Cox17* with or without 10 mM nicotinamide treatment for 4 days (mean ± s.e.m., normalized to the indicated control for corresponding experimental replicate, *n* = 3 independent experiments, two-tailed one-sample *t*-test and Wilcoxon test). **i**,**j**, Scheme (**i**) and flow cytometry of mtSOX dye in control and *Mof* iKO MEFs expressing the indicated MOF variants (**j**) (mean ± s.e.m., normalized to the mean of the corresponding iWT control, *n* = 4 embryos, two-tailed Student’s *t*-test). **k**, Immunoblot of mitochondrial lysate in MEFs for acetylated proteins MEFs expressing MTS–GFP or MTS–MOF, using pan-acetyl lysine antibody. HSP60 served as a loading control. **l**,**m**, Quantification of nuclear abnormalities (micronuclei and nuclear blebs) (*n* = 2–3 embryos) (**l**) and complex IV activity (*n* = 3–7 embryos) (**m**) in control and *Mof* iKO MEFs expressing the indicated MOF variants (mean ± s.e.m., normalized to the mean of the corresponding iWT control, two-tailed Student’s *t*-test). **n**, Scheme and complex IV activity in control and *Kansl2* iKO MEFs expressing lentivirally transduced 3xFLAG-tagged KANSL2 or its MTS-deleted mutant (KANSL2^mut^) (mean ± s.e.m., normalized to the mean of the corresponding iWT control, *n* = 3–6 embryos, two-tailed Student’s *t*-test). Source data

To further establish COX17 as a direct target of MOF and to investigate that they act in concert in their regulation of cytochrome c oxidase activity, we expressed human COX17 variants in 3T3 fibroblasts depleted of both MOF and endogenous COX17 (Fig. 4a). While a single KD of either MOF or CO17 led to reduced complex IV activity, a double KD did not lead to any further downregulation of complex IV activity, placing MOF upstream of COX17 and its acetylation. While hCOX17^WT^ and hCOX17^K18,30Q^ could rescue complex IV activity upon depletion of endogenous COX17 in the control cells, only hCOX17^K18,30Q^, but neither hCOX17^WT^ nor hCOX17^K18,30R^, could restore the original level of complex IV activity in *Mof* KD cells (Fig. 4a). These results indicate that presence of MOF is critical for acetylation of COX17 and therefore complex IV activity. Interestingly, the non-acetylated variant always resulted in the least complex IV activity while the acetylation-mimicking variant exhibited the highest complex IV activity, independent of *Mof* expression. Similar to our observation in 3T3 fibroblasts, exogenous expression of hCOX17^K18,30Q^ could rescue the complex IV phenotype in primary *Mof* iKO MEFs, whereas hCOX17^WT^ and hCOX17^K18,30R^ failed to do so (Fig. 4b). Additionally, overexpression of inactive hCOX17^C22,23A^ in *Mof* iKO MEFs also could not rescue complex IV activity (Fig. 4c). Interestingly, hCOX17^K18,30R^ failed to abrogate mtROS accumulation triggered by MOF depletion and hCOX17^K18,30Q^ could only partially lower the level of accumulated mtROS (Fig. 4d). This indicates that, while cytochrome c oxidase function is largely dependent on COX17 acetylation, additional mitochondrial targets of MOF may be involved in controlling other mitochondrial processes.

Next, we asked whether protein acetylation mediated by the MOF–KANSL complex is essential for complex IV activity. To this end, we inhibited the NAD^+^-dependent sirtuins using nicotinamide (Fig. 4e). Treating MEFs with nicotinamide and 4-hydroxytamoxifen (4OHT) simultaneously (to achieve a state where the acetylation mark deposited by MOF is maintained), and not by treating with nicotinamide 2 days after 4OHT induction, we could rescue the impaired complex IV activity inflicted upon by MOF deletion (Fig. 4f and Extended Data Fig. 6b). However, nicotinamide treatment could not rescue complex IV deficiency in the absence of COX17 (Fig. 4g). Intriguingly, nicotinamide treatment also failed to rescue the complex IV phenotype in the 3T3 fibroblasts stably expressing the non-acetylated hCOX17^K18,30R^ (Fig. 4h). In summary, these findings suggest that acetylation of COX17 mediated by MOF is essential for the regulation of complex IV activity.

### Mitochondrial MOF–KANSL complex is sufficient and necessary for CIV activity

Since MOF and a subset of KANSL complex members, KANSL1 and KANSL3, were shown to dually localize to nucleus and mitochondria in HeLa cells, we validated the finding in primary MEFs by cellular fractionation (Extended Data Fig. 6c–e). KANSL2, another member of the KANSL complex contains an N-terminal mitochondrial pre-sequence indispensable for its import in isolated yeast mitochondria^10^. We also confirmed its mitochondrial localization using cell lines stably expressing C-terminally tagged KANSL2 via structured illumination microscopy (Extended Data Fig. 6f).

Nuclear abnormalities and mitochondrial dysfunction could both be observed simultaneously upon MOF depletion; therefore, we sought to disentangle the contribution of nuclear and mitochondrial pools of MOF in regulating mitochondrial homeostasis. To this end, we performed complementation experiments in which *Mof* iWT and iKO MEFs were transduced with lentiviral constructs encoding for variants of MOF. We generated lentiviral particles containing empty vector (EV), wild-type MOF (MOF) or catalytic inactive (E350Q) MOF (MOF^mut^). Additionally, by fusing an N-terminal mitochondrial targeting sequence (MTS) and simultaneously disrupting its nuclear localization signal (NLS), we targeted MOF (MTS–MOF) and its catalytic inactive mutant (MTS–MOF^mut^) to the mitochondria (Fig. 4i). We confirmed the expression levels and cellular localization of the complementation constructs by western blot and immunofluorescence (Extended Data Fig. 6g,h). Complementation with wild-type MOF, but not with its catalytic inactive version, could rescue the elevated mtROS levels upon *Mof* iKO, validating the functionality of our exogenous MOF variants (Fig. 4j). However, mitochondrial MOF could only partially reduce the elevated mtROS levels upon *Mof* iKO, suggesting that, although not all, certain features of the observed mitochondrial phenotype are caused by lack of activity of the mitochondrial pool of MOF (Extended Data Fig. 6i,j). Additionally, we observed that loss of MOF leads to increase in the GSH:GSSG ratio, as a measure of antioxidant metabolism, that potentially also contributes to increased mitochondrial ROS production upon MOF depletion (Extended Data Fig. 6k).

A well-characterized nuclear target of MOF is lamin A/C and loss of lamin A/C acetylation at K311 results in nuclear abnormalities^22^. We observed rescue of these nuclear abnormalities with MOF but not by its catalytic inactive version, MOF^mut^, as was also shown previously^22^. However, complementation with MTS–MOF failed to rescue the nuclear abnormality phenotype in *Mof* iKO MEFs, ensuring the substrate specificity of mitochondrially targeted MOF construct (Fig. 4k). Additionally, we observed increased mitochondrial protein acetylation in MEFs overexpressing MTS–MOF, thereby verifying the catalytic activity of the mitochondrial pool of MOF (Fig. 4l). Intriguingly, complementation with both MOF and MTS–MOF in *Mof* iKO cells could restore the complex IV activity to iWT levels, while the catalytic inactive MTS–MOF^mut^ was unable to do so, suggesting that the enzymatic activity of MOF in the mitochondria is sufficient to maintain complex IV activity (Fig. 4m).

KANSL2 was previously shown to harbour a canonical N-terminal mitochondrial targeting signal, genetic deletion of which disrupted its import into isolated yeast mitochondria^10^. Following the above proof of principle, we performed complementation experiments in which *Kansl2* iWT and iKO MEFs were transduced with lentiviral constructs encoding full length KANSL2 or its MTS-deleted mutant (Fig. 4n). We confirmed the expression levels of the complementation constructs by western blot (Extended Data Fig. 6l). Complementation with full-length KANSL2, but not with its MTS-deleted mutant, could rescue the CIV activity deficit upon *Kansl2* iKO, validating the functionality of the mitochondrial pool of KANSL2 (Fig. 4n).

### Individuals with de novo mutations in *MOF* exhibit mitochondrial defects

**Fig. 5:**
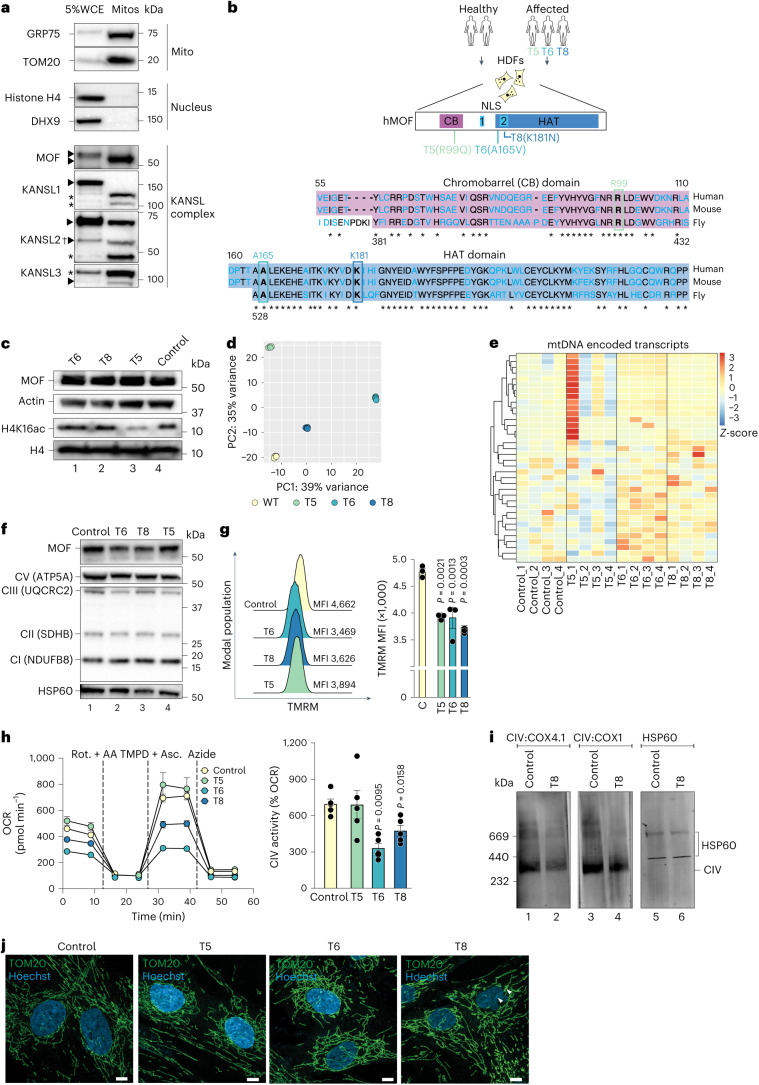
Human *MOF* haploinsufficiency manifests mitochondrial dysfunction. **a**, Immunoblot analysis of 5% cell lysate as input and enriched mitochondria for the detection of MOF–KANSL complex members in HDFs. Quality of fractionation was verified using mitochondrial and nuclear markers. Triangles denote specific protein band, and a cross is added to indicate isoform or post-translationally modified or cleaved version of the protein. Asterisks mark unknown bands enriched predominantly in mitochondrial purifications^10^. **b**, Scheme of major domains of the coding region of MOF, illustrating the position of de novo single amino acid substitution mutations harboured by the indicated patients with intellectual disability, T5, T6 and T8 (ref. ^15^). **c**, Immunoblot analysis of MOF and bulk H4K16 acetylation in HDFs derived from individuals that are either healthy or harbouring de novo mutations in *MOF*. Actin and total histone H4 served as loading controls. **d**, PCA of RNA-seq analysis showing clustering of the indicated HDF samples. **e**, Heat map of normalized counts for all detected mtDNA encoded transcripts in control, T5, T6 and T8 HDFs. **f**, Immunoblot analysis of components of mtETC using the Total OXPHOS Rodent WB Antibody cocktail in cell lysates of control, T6, T8 and T5 HDFs. HSP60 served as a loading control. **g**, Modal population (left) and quantification of median fluorescence intensity (MFI) (right) of flow cytometric analysis of mitochondrial membrane potential by TMRM dye in control, T5, T6 and T8 HDFs (mean ± s.e.m., *n* = 3 independent experiments one-way analysis of variance (ANOVA) followed by Dunnet’s multiple comparison). **h**, OCR (left) and complex IV activity (right) in control, T5, T6 and T8 HDFs (mean ± s.e.m., *n* = 5 independent experiments, one-way ANOVA followed by Dunnet’s multiple comparison). **i**, Immunoblot analysis of BN-PAGE-separated mtETC complexes of mitochondria isolated from control and T8 HDFs for assemblies containing complex IV. HSP60 served as a loading control. **j**, Immunofluorescence microscope images of control, T5, T6 and T8 HDFs with antibodies against mitochondrial TOM20 (green) and nuclear Hoechst (blue). Scale bar, 10 µm. White arrowheads indicate nuclear abnormalities. Source data

Patients harbouring heterozygous de novo single nucleotide mutations in the coding sequence of *MOF* have recently been identified through exome sequencing projects carried out around the world. They are commonly diagnosed with global developmental delay, intellectual disability, epilepsy and other developmental anomalies^15^. While all the mutant forms of MOF resulted in diseased phenotype in individuals, corresponding recombinant versions exhibited a high degree of heterogeneity in vitro in the most anticipated effect on H4K16 acetylation. Therefore, to better understand the pathophysiology underlying the clinical manifestations of MOF syndrome, we performed functional assays with primary human dermal fibroblasts (HDFs) derived from healthy individuals and patients with MOF syndrome. We validated the presence of MOF–KANSL complex members in mitochondria enriched from HDFs (Fig. 5a).

Next, we leveraged HDFs, harbouring driving mutations in MOF in its chromobarrel (T5) or its catalytic (histone acetyltransferase or HAT) domains (T6 and T8) (Fig. 5b, top). We found the mutated residues of MOF to be conserved across species (Fig. 5b, bottom). HDFs from all the individuals had similar protein levels of MOF; however, while all three patients, T5, T6 and T8, exhibited global developmental delay, loss of bulk H4K16 acetylation levels was observed only in HDFs derived from patient T5 (Fig. 5c). Additionally, patient T8 showed anomalies in the cardiac muscle, a tissue that requires high mitochondrial metabolism. Together, this indicated that non-histone acetylation by MOF could serve as a potential mechanism by which the disease phenotypes in the patients with MOF syndrome are manifested.

**Extended Data Fig. 7:**
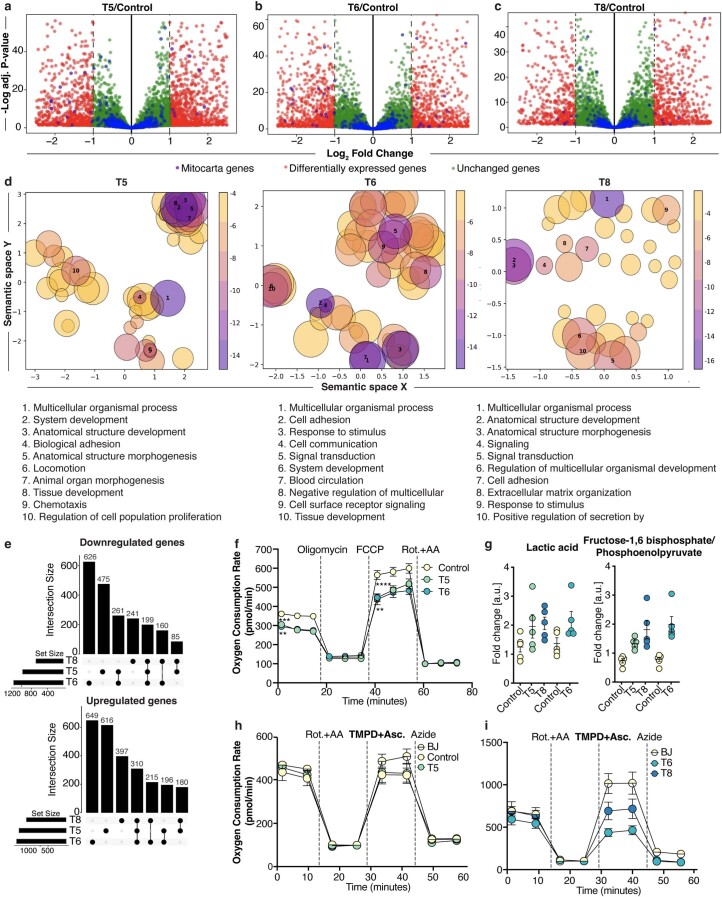
Transcriptional effects of *MOF* heterozygous *de novo* mutations. **a-c**, RNA-seq analysis of HDFs from MOF syndrome patients, T5 **(a)**, T6 **(b)** and T8 **(c)** (n = 4 technical replicates). Volcano plots show all detected transcripts, with vertical dashed lines indicating the Log_2_ FC cutoff (calculated by Wald Tests). All detected transcripts belonging to human Mitocarta 3.0 protein transcripts are highlighted in blue. **d**, Bubble plots show summarized biological processes affected by transcriptional downregulation in T5, T6 and T8 HDFs. Size of bubbles depicts the number of terms belonging to the summary term whereas the color shows their Log_10_ p-value. The position of the term signifies their relation in semantic space. The 10 most affected processes are listed below each plot. **e**, Upset plots show overlap of differentially downregulated **(upper)** and upregulated **(lower)** amongst T5, T6 and T8 HDFs. Number of overlapping genes are indicated in the panel. **f**, Oxygen Consumption Rate (OCR) in control, T5 and T6 HDFs measured by sequential treatment with indicated drugs (mean ± s.e.m., n = 9-38 independent samples, two-tailed Student’s t-test was performed on the first time point of basal respiration and on the first time point of respiration following FCCP injection and was calculated using. ****p-value < 0.0001, ***p-value < 0.001, **p-value < 0.01. **g**, Metabolite analysis in control, T5, T6 and T8. Data is normalized to total protein content (mean fold change ± s.e.m., n = 5 independent experiments, one-way ANOVA followed by Dunnet’s multiple comparison). **h, i**, OCR with sequential indicated treatments. BJ, control and T5 fibroblasts shows similar levels of complex IV activity **(h)**. T6 and T8 HDFs show lower complex IV activity as compared to the control BJ cells **(i)**. BJ fibroblasts from ATCC were used as an independent control to complement complex IV activity of the HDFs (mean ± s.e.m., n = 3-9 independent samples). Source data

We first performed RNA-seq analysis on the HDFs to gain insights into the transcriptional changes brought about by mutations in *MOF*. Each HDF line was distinct without any major clustering on the PCA plot, pointing towards diverse genetic backgrounds and/or possible distinct impacts of the mutations on MOF (Fig. 5d). Fibroblasts from individuals T5, T6 and T8 each exhibited a large number of genes differentially regulated when compared with the control (Extended Data Fig. 7a–c). On comparing among the HDF groups, we found 199 genes and 310 genes to be commonly downregulated and upregulated respectively (Extended Data Fig. 7e). Analysis of the biological processes, expressed as Gene Ontology term summaries, associated with the downregulated transcripts indicated dysregulated developmental processes and signal transduction, but no major mitochondrial pathways to be affected upon *MOF* mutation (Extended Data Fig. 7d). Moreover, there were no major changes in the transcripts encoded by the mitochondrial genome as shown by the heat map summarizing their relative counts among the control and the patient cells (Fig. 5e). The protein levels of the mtETC components also remained largely unchanged (Fig. 5f).

The patient HDFs showed reduced membrane potential compared with the healthy subject (Fig. 5g). Similar to the phenotype observed in primary MEFs, the patient HDFs also exhibited impaired mitochondrial respiration and accumulation of glycolytic intermediates (Extended Data Fig. 7f,g). Interestingly, while HDFs from the two individuals harbouring mutations in the HAT domain, T6 and T8, showed a severe defect in the activity of complex IV, HDFs from the individual harbouring mutation in the chromobarrel domain, T5, showed similar levels of complex IV activity relative to the control (Fig. 5h and Extended Data Fig. 7h,i). This indicates that the enzymatic activity, but not the chromatin-binding function of MOF, is indispensable for promoting mitochondrial cytochrome c oxidase activity. We also observed lower assembled complex IV levels in T8 patients with MOF syndrome as compared with the control as measured in blue native gel electrophoresis using antibodies against COX1 and COX4.1 (Fig. 5i). The mitochondrial morphology, however, showed no obvious differences (Fig. 5j). We reasoned that the effects of heterozygous mutations might not be fully penetrant in all the aspects of mitochondrial biology and that, in the scenario of chronic reduced activity of MOF, cells might have developed an as-yet-unknown mechanism of adaptation.

**Fig. 6:**
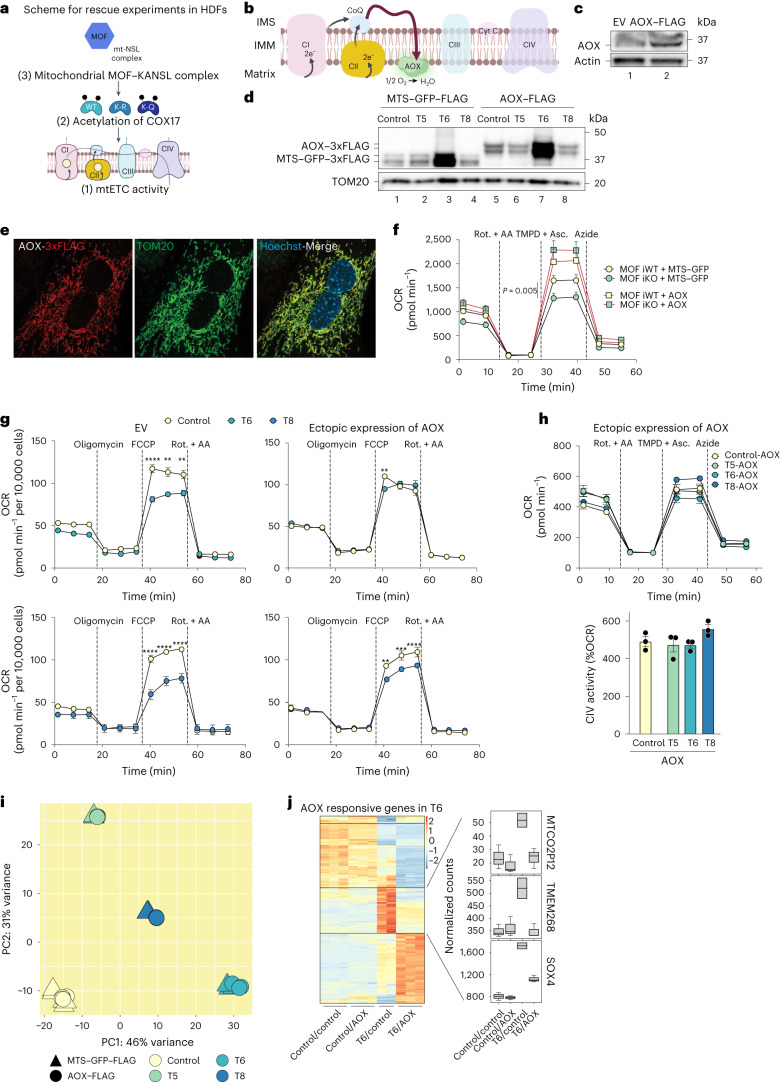
Ectopic expression of alternate oxidase restores mitochondrial dysfunction in MOF-deficient mouse and MOF syndrome patient cells. **a**, Scheme for rescue experiments on dermal fibroblast. **b**, Placement of AOX from *C. intestinalis* in mammalian mtETC function. Scheme was adapted from BioRender. **c**, Immunoblot analysis for ectopic expression of AOX in MEFs. β-Actin served as a loading control. **d**, Immunoblot analysis for expression of MTS–GFP or AOX in the indicated HDFs. TOM20 served as a loading control. **e**, Representative immunofluorescence microscopy image for ectopic expression of AOX in MEFs using anti-FLAG antibody. TOM20 (green) and Hoechst (blue) served as mitochondria and nuclear markers for immunofluorescence. Scale bars, 10 µm. All transgenes are C-terminally 3xFLAG-tagged and expressed via a lentiviral vector. **f**, OCR in MEFs, expressing AOX in control and *Mof* iKO MEFs (*n* = 3 embryos). Black lines correspond to GFP (control)-expressing cells, and red lines correspond to AOX-expressing cells (mean ± s.e.m., *n* = 7–11 independent samples, two-tailed Student’s *t*-test; *P* value refers to the first timepoint after TMPD injection). **g**, OCR in indicated HDFs, expressing control or AOX transgene (mean ± s.e.m., *n* = 3–8 independent samples, two-tailed Student’s *t*-test is indicated for timepoint after TMPD injection in the panel; ****P value <0.0001, ***P value <0.001, **P value <0.01). **h**, OCR (top) and complex IV activity (bottom) in the indicated HDFs upon AOX expression (mean ± s.e.m., *n* = 3 independent experiments). **i**, PCA of RNA-seq analysis showing clustering of the indicated HDFs transduced with control or AOX. **j**, Heat map showing clustered DESeq-normalized counts from GFP–FLAG (control) or AOX–FLAG transduced control and patient T6 HDF lines for RNA transcripts that are differentially upregulated or downregulated in T6 lines upon AOX expression (*n* = 2–3 independent experiments with median (centre) line, first (top) and third (bottom) quartiles and the error bars with maximum to minimum range). Source data

We undertook various approaches to extend our findings from mouse and cellular models to patient-derived primary cells (Fig. 6a). Alternative oxidase (AOX) from *Ciona intestinalis* transports electrons directly from ubiquinol to oxygen thereby bypassing the cytochrome segment of the mtETC comprising complex III and complex IV (Fig. 6b)^52^. We stably expressed MTS–GFP or AOX in iWT and *Mof* iKO MEFs (Fig. 6c,e). Remarkably, AOX restored the impaired respiration upon loss of MOF thereby highlighting the cytochrome segment as a direct target of MOF (Fig. 6f). We then expressed AOX in patient HDFs (Fig. 6d). AOX was able to fully or partially rescue the mitochondrial respiration defect in T6 and T8 HDFs, respectively (Fig. 6g). Furthermore, AOX could compensate for the impaired complex IV activity that was caused by the *MOF* mutations (Fig. 6h). Taken together, these data indicate that the cytochrome segment is a conserved target of MOF in mice and humans and suggest that the pathological de novo mutations of *MOF* disrupt its function not only in nuclear transcription regulation, but also in the maintenance of mitochondrial energy metabolism.

We also performed RNA-seq experiments on HDFs derived from the patients which were transduced with lentiviral particles containing either control (MTS–GFP) or AOX transgenes. The PCA plot of the RNA-seq experiment (Fig. 6i) showed that the patient fibroblasts transduced with control or AOX from each individual still cluster together, indicating that AOX had no major contribution to the transcriptional defects inflicted by *MOF* mutations. However, intriguingly, analysis of this dataset (Fig. 6j) shows that AOX expression leads to more dramatic transcriptional changes in the T6 patient compared with T5 or T8. By comparing the normalized RNA-seq counts of control and T6 samples transduced with either MTS–GFP or AOX, we found 109 transcripts whose expression was either partially or fully restored by exogenous expression of AOX. It was really interesting to see that 93 out of these 109 transcripts were initially upregulated in T6 patients compared with the healthy individuals, indicating indeed a possible secondary effect of *MOF* mutation through mitochondrial defects.

**Fig. 7:**
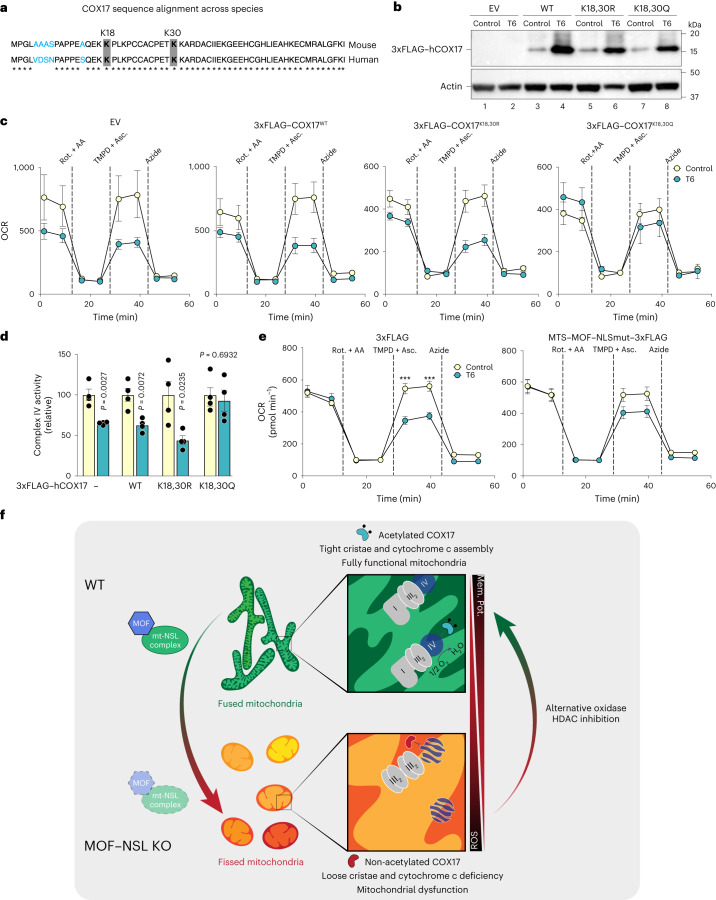
Implication of COX17 acetylation via MOF is conserved in human *MOF* haploinsufficiency. **a**, Scheme sequence conserved of COX17 among species. **b**, Immunoblot analysis for expression of COX17 variants in HDFs from patient T6. Actin served as a loading control. **c**,**d**, Representative OCR profiles (**c**) and complex IV activity (**d**) in control and T6 HDFs expressing the indicated COX17 variant (mean ± s.e.m., *n* = 4 independent experiments, two-tailed Student’s *t*-test). **e**, OCR in control and T6 HDFs, expressing mitochondrial MOF (mean ± s.e.m., *n* = 10–13 independent samples, two-tailed Student’s *t*-test and is indicated for timepoint after TMPD injection in the panel. ***P value <0.001). **f**, A schematic model for the role of MOF–KANSL complex in maintenance of mitochondrial structure and function. MOF–KANSL complex governs structural and functional integrity of mitochondria by acetylation of specific mitochondrial proteins. Loss of MOF-mediated COX17 acetylation leads to cytochrome c oxidase deficiency, mitochondrial fragmentation and reduced cristae density. Impaired mtETC complex IV activity arising from imbalance in acetylation dynamics in the absence of MOF was restored by HDAC inhibition, constitutive expression of acetylation-mimicking COX17 variant or AOX. Source data

Next, we verified the sequence conservation of COX17 from mouse to human and asked if the impaired complex IV activity in the patient HDFs was a result of reduced COX17 acetylation (Fig. 7a). To this end, we overexpressed wild-type COX17 and its acetylated and non-acetylated mimics in control and T6 HDFs (Fig. 7b). We observed that exogenous expression of COX17^WT^ and COX17^K18,30R^ could not rescue the complex IV phenotype in T6 HDFs. On the other hand, exogenous expression of COX17^K18,30Q^, which functions as a constitutively acetylated mimic independent of the acetyltransferase activity of MOF, was able to restore complex IV activity in T6 HDFs (Fig. 7c,d). Finally, we expressed MTS–MOF in the T6 HDFs and observed that we could rescue the impaired complex IV activity (Fig. 7e). In summary, COX17 is an acetylation target of the mitochondrial pool of MOF in humans and could potentially contribute to the clinical manifestation of mutations in *MOF*.

## Discussion

In this study, we have mechanistically characterized the mitochondrial dysfunction manifested by deregulation of MOF–KANSL complex members in primary mouse cells and by mutations in the coding region of *MOF* in human patient-derived cells (Fig. 7f). To our surprise, the transcriptional alterations upon *Mof* KO had severe implications on a range of cellular processes, but could not simply explain the striking destabilization of the mtETC. We identified a unique set of mitochondrial proteins that undergo a change in their acetylation status upon loss of MOF, without any significant fluctuation in their steady-state RNA or protein levels. Our study reveals that MOF, as a classical epigenetic regulator, has an impact on cellular physiology via protein acetylation in compartments outside of the nucleus.

The functional consequences of acetylation have so far been studied for only about one-tenth of all acetylated mitochondrial proteins^5^. Acetylation of the majority of metabolic enzymes renders them inactive^5,53^. The few exceptions to this discovered so far include aconitase (ACO1) and aldehyde hydrogenase 2 (ALDH2), where acetylation of certain lysine residues stimulates the enzymes^54,55^. Very little is known about how acetylation of mitochondrial proteins alters their biochemical properties. Here we demonstrate that acetylation of COX17 could promote assembly of complex IV, while loss of its acetylation impaired it, exhibiting an unprecedented gain of function via acetylation of an mtETC protein. COX17 initiates functional assembly of complex IV by transferring copper ions to other assembly factors, for example SCO1, whose pathogenic mutation leads to cytochrome c oxidase (COX) deficiencies^56,57^. COX17 also interacts with the MICOS complex in yeast and humans and promotes mitochondrial cristae integrity^49,58^. Nevertheless, the precise sequence of events upon loss of COX17 acetylation leading to mitochondrial dysfunction remains to be studied.

Beyond COX17, we have also detected further mitochondrial acetylation targets of MOF. Some of these acetylation sites had been previously reported and characterized, confirming the specificity of our acetylome. For example, acetylation of FASN restrained lipogenesis required for tumour growth by controlling its stability^59^. Loss of acetylation at VDAC2-K74 was shown to be correlated with reduced sperm motility in mice and humans^60^. It would be very interesting to also study the functional relevance of these acetylation sites directly in the context of *MOF* deficiency. Given the tissue-specific nuclear roles of MOF–KANSL complex, it would be of utmost interest to generate MOF acetylomes in different mouse tissues, which would also enable us to investigate the substrate specificity of mitochondrial MOF in greater detail^12,17,61,62^.

Non-enzymatic protein acetylation is aided by high concentration of acetyl-CoA and alkaline pH of the mitochondrial matrix but cannot explain acetylation of all mitochondrial substrates^7,21^. Especially for IMS proteins such as COX17, a non-enzymatic acetylation might be unfavourable since the pH of mitochondrial IMS is around 7.0 and acetyl-CoA concentration is not as high as in the matrix^63^. GCN5L1, a homologue of the nuclear acetyltransferase GCN5, enriches in the mitochondria, and its levels are directly correlated to the global acetylation levels of mitochondrial proteins^64^. However, the fact that GCN5L1 lacks a catalytic domain raises questions about its enzymatic activity in the mitochondria. Here we could show that the mitochondrial pool of MOF, but not the corresponding catalytic inactive mutant, was sufficient to rescue complex IV defects triggered by loss of MOF, strongly indicating non-spurious acetylation that is mediated by the enzymatic activity of MOF. Mitochondrial MOF has been shown to be distributed over the mitochondrial subcompartments, and therefore precise site of acetylation of individual targets would be important to understand the dynamics of the MOF–KANSL complex. Eclipsed distribution of MOF in the nucleus and the mitochondria paves the way for in-depth study of its shuttling between the two organelles^65^.

Interestingly, we observed hyperacetylation of several mitochondrial proteins upon *Mof* as well as *Kansl2* iKO. While the hypoacetylated residues localized in different subcompartments of the mitochondria, the majority of hyperacetylated residues are found on proteins that localize to the matrix or on mitochondrial inner membrane proteins with acetylation sites facing towards the matrix. The major metabolic phenotype upon loss of MOF, that is, OXPHOS deficiency, has been previously shown to cause an accumulation of excess acetyl-CoA in mitochondria^66^. This effect can enhance the non-enzymatic acetylation of matrix and inner membrane proteins as a secondary effect^5^.

Even though here we focused on its lysine acetyltransferase activity, recent studies have suggested additional catalytic functions for MOF in the context of short-chain acylation of lysine residues. MOF not only catalyses histone crotonylation but also propionylation of multiple nuclear and non-nuclear proteins^67,68^. Given the fact that the levels of short-chain acyl groups strongly fluctuate depending on the nutrition and cellular metabolic fitness, local high levels of such intermediates might compete with acetyl-CoA as co-factors and dictate their incorporation into different protein targets via MOF. It is intriguing to speculate the functional relevance of these modifications on mitochondrial proteins thereby expanding the regulatory repertoire of mitochondrial function.

The mechanism underlying the respiratory defects in MOF-depleted primary MEFs is attributed to mitochondrial protein hypoacetylation, which is already prominent under glucose culture conditions. On the contrary, while respiration defects persisted in cancerous HeLa cells under non-aerobic as well as aerobic metabolic conditions, the effects in mtDNA transcription upon MOF–KANSL complex depletion was observed only in aerobically respiring cells. Multiple key differences exist in the mitochondrial physiology and metabolism between primary cells such as MEFs and cancerous cells such as HeLa: MEFs are oxidative in their nature with high mitochondrial respiratory capacity, whereas HeLa cells are rather glycolytic with relatively much lower mitochondrial respiratory capacity. mtDNA dynamics and mitochondrial stress response is also very variable between these cell types: the mtDNA of HeLa cells can not only withstand relatively high levels of oxidative damage before degradation, but also recovers faster from the assault. On the contrary, a much lower dose of oxidative damage is sufficient to degrade the mtDNA of MEFs^69^. These observations therefore call for further analyses regarding the precise mechanism of galactose adaptation in cells containing healthy mitochondria compared to cells which exhibit poor mitochondrial function. Moreover, it would be important to determine if direct acetylation of OXPHOS proteins and binding of MOF to the mtDNA are mutually exclusive or rather depend on cell type and growth conditions.

Pathophysiology associated with *MOF* and *KANSL1* haploinsufficiency displays developmental and neurological impairments, which are also hallmarks of inherited mitochondrial diseases, including those of encephalomyopathy and Leigh syndrome associated with cytochrome c oxidase (COX) deficiency^15,20,70^. We extended our findings in mouse and cellular models to human patient-derived cells, to better understand the possibility of a treatment regimen in these patients targeting the mitochondrial contribution of MOF. Our study strongly supports the idea that changes in transcription alone are not sufficient to explain the complexity of disease pathology of *MOF* mutations, which is specially underscored by distinct phenotypes in MOF syndrome patients. Multi-omics characterization of different tissue samples from patients with MOF syndrome would be invaluable towards a better understanding of the crosstalk between epigenetics and metabolism and for the development of subsequent therapeutic strategies. On the verge of discovering clinically relevant biology of epigenetic factors involved in diverse context-specific functions, further studies are required to untangle transcriptional versus post-transcriptional regulation that will be probably coupled with histone versus non-histone post-translational modifications. Taken together, multi-faceted roles of MOF-associated complexes across the nucleus and mitochondria highlight just one example of the complex biology hidden behind the epigenetic regulators and paves the way for new avenues to explore inter-organellar communication in healthy and diseased states.

## Methods

### Cell culture

Cre-ERT2 T/+ *Mof*^fl/fl^, *Kansl1*^fl/fl^, *Kansl2*^fl/fl^ and *Kansl3*^fl/fl^ MEFs were generated by tryptic digestion of E13.5 embryos from pregnant 7–12-week-old females mated with corresponding CAGG Cre-ERT2 T/+ males from pure C57BL/6J background mice. Primary cells and cell lines were maintained in Dulbecco’s modified Eagle medium (DMEM, Gibco #31966-021); supplemented with 10% heat-inactivated foetal calf serum (FCS), 100 U ml^−1^ penicillin and 100 μg ml^−1^ streptomycin (Gibco #15140-122); and grown in a humidified incubator at 37 °C and 5% CO_2_. Cell culture medium was replenished every alternate day, and cells were split at a maximum confluency of 90% using 0.25% trypsin–ethylenediaminetetraacetic acid (EDTA) (Gibco #25200056) or frozen in 90% FCS and 10% dimethyl sulfoxide (Sigma #8418) for storage. All the cell lines used in this study were tested regularly for mycoplasma contamination. To induce gene KO in MEFs, 0.75 million cells per 10 cm culture dish or 2 million cells per 15 cm culture dish were seeded and were cultured in the presence of 2 μM 4-OHT (Sigma #SML1666) for 4 days. Cells were used for experiments up to a maximum of sixth passage number.

All experiments involving animals were performed according to the German animal care and ethics legislation. The protocols applied have been evaluated and approved by the local government authorities, the Committee on Research Animal Care and the Regierungspräsidium Freiburg. This project was performed according to the anzeigepflichtiges Versuchsvorhaben (notifiable experimental project) with the relevant licence ‘Akh-iTo-2’ (Toetung ohne Vorbehandlung) and approved by the Max Planck Institute of Immunobiology and Epigenetics, welfare officer Dr Stefanie Kunz.

Flp-In 3T3 host fibroblasts were obtained from Thermo Fisher (#R76107) and HeLa Flp-In TRex host cell line was a kind gift from Prof Stephen Taylor, University of Manchester. The Fip-In cells were maintained in 100 μg ml^−1^ Zeocin (Invitrogen #R25001). COX17 and KANSL2 were amplified from complementary DNA prepared from human cells and cloned into pEF5/FRT/V5-DEST and pcDNA5/FRT/TO respective for generating C-terminal V5-tagged COX17 and N-terminal HA-3xFLAG KANSL2 stable cell lines, according to the manufacturer’s instructions. Cells were selected for the insert and maintained in growth medium containing 200 μg ml^−1^ hygromycin (Gibco #10687010).

HEK293T cells were obtained from Thermo Fisher and used for lentiviral production. HDFs derived from individuals who are either healthy or have been identified to harbour heterozygous de novo mutations in the coding sequence of *MOF* (patients described in ref. ^15^) were a kind gift from Dr Philippe M. Campeau, Paediatric Department, CHU Sainte-Justine Hospital, University of Montreal, Quebec, Canada. T5 HDFs were a kind gift from Dr J. M. van Hagen, Amsterdam University Medical Center. T6 HDFs were a kind gift from Dr P. B. Agrawal, Boston Children’s Hospital. T8 HDFs were a kind gift from Dr H. Kingston, St. Mary’s Hospital, Manchester, UK. BJ human foreskin fibroblasts were obtained from ATCC (CRL-2522) and used as an independent control for experiments with HDFs. All HDFs were tested for human immunodeficiency virus (HIV)-1/HIV-2, hepatitis B virus, hepatitis C virus and severe acute respiratory syndrome coronavirus 2 before use.

For galactose conditioning and adaptation experiments, cells were grown in media containing DMEM base (Gibco #11966025) supplemented with 25 mM galactose (Sigma #G5388),1 mM sodium pyruvate (Gibco #11360-070), 10% heat-inactivated FCS, 100 U ml^−1^ penicillin and 100 μg ml^−1^ streptomycin. For cell growth analysis, equal number of cells from each sample to be compared was seeded and live cell numbers were counted using 0.4% trypan blue solution (Gibco #15250061) after 48 h. Cell numbers were normalized to the control in the respective experiment. For HDAC inhibition, 2 million cells per 15 cm culture dish were seeded and medium was supplemented with 10 mM nicotinamide.

### Lentivirus production and transduction

Lentiviral particles were produced in HEK293T by co-transfection of 10 μg lentiviral construct (backbone Addgene #22661), 3.5 μg of pMD2.G envelope plasmid and 7 μg of psPAX2 packaging plasmid using 125 mM calcium chloride diluted in BBS buffer (25 mM BES, 140 mM NaCl, 0.75 mM Na_2_HPO_4_). Supernatant containing the lentiviral particles was collected 72 h after transfection, filtered through 0.45 μm polyethersulfone membrane syringe filters (Sarstedt #83.1826) and concentrated using Amicon Ultra-15 filters with 100 kDa (#UFC9100) molecular weight cut-off. For transduction of MEFs and 3T3 fibroblasts, 0.2 million cells were plated on each well of a six-well plate and sufficient virus was added to the medium in a way as to achieve equal expression of each construct among samples (as validated by western blotting). After overnight incubation, the virus-containing medium was replaced with fresh medium containing appropriate selection antibiotic (10 μg ml^−1^ blasticidin or 1 μg ml^−1^ puromycin). All lentiviral constructs are registered with the Regierungspräsidium Tübingen.

Pre-designed and validated shRNA-pLKO.1 (control or against target proteins) constructs for constitutive KD were purchased from Sigma (MISSION series), and lentiviral particles were produced using appropriate envelope and packaging plasmids. shRNA (against GFP or target proteins) constructs for inducible KD were generated using CS-RfA-ETBsd (Riken #RDB07917), and lentiviral particles were produced using packaging and envelope plasmids, pCAG-HIVgp (Riken #RDB04394) and pCMV-VSV-G (Riken #RDB04392), respectively. Transduced and 20 μg ml^−1^ blasticidin-selected cells were induced for KD using 1 μg ml^−1^ doxycycline (Gibco #A1113903) for 4 days before performing the experiments. Sequences of the shRNAs could be found in Supplementary Table 9.

### Confocal microscopy

Cells grown up to a maximum of 80% confluency on coverslips or eight-well glass bottom Ibidi chambers (#80827) were washed once with warm phosphate-buffered saline (PBS; Life #10010023) and fixed with pre-warmed 1% formaldehyde (Thermo #28906) diluted in growth medium for 10 min at 37 °C. They were then washed twice with PBS, permeabilized with 0.2% Triton X-100 (Sigma #T8787) in PBS for 15 min at room temperature and blocked with 5% FCS and 0.1% Tween-20 (diluted in PBS) for 1 h at room temperature. Cells were incubated with primary antibody (diluted in the blocking solution) overnight at 4 °C with gentle shaking. The next day, the cells were washed thrice with blocking solution, 15 min each, with gentle shaking and stained with appropriate fluorophore-conjugated Alexa secondary antibodies (from Molecular Probes, diluted in blocking solution) for 2 h at room temperature in the dark. Cells were washed thrice with a blocking solution, 15 min each. Then 20 μM Hoechst 33342 (Thermo #62249, stock 20 mM) or 4′,6-diamidino-2-phenylindole (Molecular Probes #D1306) diluted in PBS, was used to stain the nuclei for 10 min. Cells were finally washed once with PBS before mounting in FluoroGel (#GTX2814). Confocal images were acquired using an inverted confocal laser scanning microscope Zeiss LSM 780 or Zeiss LSM 880 equipped with an Airyscan2 detector. Samples for high-resolution structured illumination microscopy were prepared in a similar manner as described above. Images were acquired using Elyra PS1 microscope, with combined structure illumination module and Zeiss LSM780 confocal scanning unit, with Plan-apochromat 100× oil 1.46 numerical aperture objective and an Andor iXon DU 885 EM-CCD camera. High-resolution structured illumination microscopy and confocal image stacks of the same field of view were acquired sequentially with the same voxel size setting. For tracking mitochondria over time using live cell imaging, MEFs were seeded on each well of eight-well glass-bottom Ibidi chambers and transduced with BacMam 2.0 reagents, MTS–GFP and NLS–red fluorescent protein (RFP) (Cell Light series from Molecular Probes). Acquisition was started at least 16 h after transduction. Laser power was set to a maximum of 3% to minimize photobleaching and phototoxicity. Time lapse imaging was performed using Zeiss Observer Z1 inverted spinning disk microscope, equipped with CS-X1 scan head (Yokogawa) and an EM-CCD camera, using standard filter sets. The laser power was set to a maximum of 3% to minimize photobleaching and phototoxicity. Images were acquired at different focal planes with appropriate *Z*-stack settings to cover the width of the cell. Images were processed, wherever required, on Zen2 Blue Software (version 3.1 and 3.2) or Zen Black Software (version 2012, Service pack 5) by Zeiss and analysed using FIJI. Mitochondrial branch length was measured using the FIJI (FIJI Is Just ImageJ version 1.0) macro, Mitochondrial Network Analysis (MiNA); the source code is available on GitHub.

### TEM

A total of 550,000 cells were seeded per well of 24-well plates the evening before sample preparation. Next morning, cells were washed once with warm PBS and fixed in 2.5% glutaraldehyde in 0.1 M sodium cacodylate buffer pH 7.4 for 1 h at 4 °C. Cells were then washed once with sodium cacodylate. Samples were post-fixed with 1% osmium tetroxide and 1% potassium ferrocyanide in 0.1 M sodium cacodylate buffer pH 7.4 for 1 h at 4 °C. After three water washes, samples were dehydrated in a graded ethanol series and embedded in an epoxy resin (Sigma Aldrich). Ultrathin sections of 60–70 nm were obtained with an Ultratome Leica Ultracut EM UC7 ultramicrotome, counterstained with uranyl acetate and lead citrate and viewed with a Tecnai G2 (FEI) transmission electron microscope operating at 100 kV. Images were captured with a Veleta (Olympus Soft Imaging System) digital camera. Images were analysed using FIJI.

### Cellular assays

#### Senescence assay

Early-passage MEFs were seeded onto six-well plates, and the KO was induced with 4-OHT as previously described. On day 5 of the KO, the medium was removed and the senescence assay was performed according to the manufacturer’s instructions (Millipore, KAA002). The images were taken using an Olympus CKX53 microscope equipped with an EP50 camera.

#### GSH:GSSG assay

A total of 10,000 cells per well were plated onto white flat-bottom tissue culture-treated plates 16 h before the assay. The assay was performed according to the manufacturer’s instructions, and the detection was performed using the Victor Nivo Plate Reader.

### Isolation of mitochondria

All the steps for isolation of intact mitochondria were performed on ice using pre-chilled buffers and equipment. Dishes with 90–100% confluent cells were washed once with PBS and then collected in PBS by scraping gently. Cellular pellet was collected by spinning, washed once with PBS and then resuspended in 5× pellet volume of mitochondrial isolation buffer (MIB: 220 mM mannitol, 70 mM sucrose, 1 mM EDTA and 20 mM HEPES–KOH pH 7.4), supplemented with 2 mg ml^−1^ BSA and protease inhibitor mix (Roche). Cells were swollen for 15 min with gentle rotation and dounced 30 times in type-B glass homogenizer. Unbroken cells and nuclei were pelleted at 1,000*g* for 10 min. The supernatant was collected for high-speed centrifugation, and the pellet was resuspended in 2× pellet volume of MIB for a second round of dounce homogenization. The pooled supernatant was pelleted at 13,000*g* for 20 min to obtain crude mitochondria. The crude mitochondrial pellet was washed twice with MIB, resuspended in appropriate volume of MIB and measured for protein concentration using Quick Start Bradford reagent (Bio-Rad). For verification of mitochondrial localization of proteins, crude mitochondria were further purified using a 15–50% discontinuous Percoll (GE Healthcare) gradient in MIB. Percoll was added to the crude mitochondria to a final concentration of 15% concentration and was overlaid on top of the gradient. The gradient was centrifuged at 30,700*g* for 15 min, and purified intact mitochondria were obtained from the 22–50% gradient interface. The extracted fraction was washed twice in MIB to remove excess Percoll and finally pelleted at 13,000*g* for 20 min at 4 °C. Crude and pure mitochondria were used immediately after isolation or stored at −80 °C.

### Immunoblotting

Cellular pellet was resuspended in sodium dodecyl sulfate (SDS) loading dye (Roti-Load, Carl Roth) at a concentration of 1 million cells per 100 μl. Samples were boiled at 95 °C for 10 min, sonicated using Branson Sonifier 250 (40% duty cycle, 1.5 output, 20 pulses) and boiled again for 5 min. Mitochondrial samples were heated at 50 °C for 10 min. SDS–PAGE was performed on 4–12% NuPAGE pre-cast Bis-Tris gels (Invitrogen #NP0321PK2) and run in MOPS buffer (Novex #NP0001). For detection of proteins with molecular weight higher than 30 kDa, proteins were transferred to a 0.45 μm polyvinylidene fluoride membrane, otherwise a 0.22 μm membrane was used. Following the wet transfer, membranes were blocked with 5% (w/v) skimmed milk in 0.3% (v/v) Tween–PBS for 1 h at room temperature and incubated with relevant primary antibodies. After washing thrice for 5 min each with 0.3% Tween–PBS, membranes were incubated with horseradish peroxidase-conjugated mouse or rabbit IgG for 2 h at room temperature. The membrane was washed thrice for 5 min each with 0.3% Tween–PBS and finally developed using Lumi-Light western blotting substrate (Roche #12015196001) on Chemidoc (Bio-Rad).

### BN-PAGE and in-gel activity staining

BN-PAGE was performed according to published protocols^71^. Briefly, 60 µg of crude mitochondria from each sample was solubilized in 60 µl of 1% (w/v) digitonin or dodecyl-maltose, diluted in digitonin buffer (20 mM Tris–HCl pH 7.4, 0.1 mM EDTA, 50 mM NaCl and 10% (v/v) glycerol) and incubated on ice for 15–30 min. A total of 100 µg of crude mitochondria was used for mitochondria derived from HDFs. Samples were then pelleted at 13,000*g* for 10 min, and the supernatant was mixed with 6.6 µl of 10× BN-PAGE loading dye (5% (w/v) Coomassie blue G, 500 mM *e*-amino-*n*-caproic acid and 100 mM Bris-Tris pH 7.0). Samples were loaded on pre-chilled native 4–13% acrylamide continuous gels. Gels were run at 600 V until the protein samples entered the gel completely. The Coomassie R-250 containing buffer (50 mM tricine, 15 mM Bis-Tris pH 7.0 and 0.02% (w/v) Coomassie G) was then replaced with cathode buffer without the dye, and the gels were allowed to run at 300 V till an optimum separation was observed. After the electrophoresis, the gel was incubated in SDS buffer for 10 min, and the proteins were transferred to a 0.45 µm polyvinylidene fluoride membrane. After the semi-dry transfer, the membrane was stained briefly with Coomassie (0.2% (w/v) Coomassie R-250, 40% (v/v) ethanol and 10% (v/v) acetic acid) to allow visualization of the ladder and then destained thoroughly using destaining solution (40% (v/v) ethanol, 10% (v/v) acetic acid) and finally in methanol. Membranes were blocked with 5% milk diluted in TBS (20 mM Tris–HCl pH 7,5 and 125 mM NaCl) supplemented with 0.1 % (v/v) Tween-20 and subsequently incubated with primary antibodies in the blocking solution for 2 h. Primary antibodies were washed thrice and then incubated with secondary antibodies for 2 h. For complex IV in-gel staining, gels were incubated for 10 min in freshly prepared 50 mM phosphate buffer pH 7.4, containing 15 mg 3,3′-diaminobenzidine and 25 mg cytochrome c. After development of colour, indicative of 3,3′-diaminobenzidine polymerization, gels were scanned for image acquisition^72^.

### RNA extraction, qRT–PCR and RNA-seq

RNA was extracted from cells using TRIzol reagent (Invitrogen) and Direct-Zol RNA Miniprep kit (Zymo) according to the manufacturer’s instructions. RNA concentration was measured using Qubit 2.0, and reverse transcription was performed using random hexamers according to Promega Goscript kit protocol. qRT–PCR was performed with FastStart SYBR mix (Roche). Total RNA of high quality was used for library preparation using TruSeq Total RNA RiboZero Plus kit. Libraries were sequenced in paired-end mode with 100 bp reads to a depth of 30 million per sample. Total RNA-seq was processed using default parameters of SnakePipes v.2.5.1 non-coding RNA-seq pipeline with -trim option^73^, subread package (v.2.0.0). Downstream analysis and visualization was performed using featureCounts (v.2.0.0), GOFigure and R packages DESeq2 (v.1.34.0), ggplot2 (v.3.3.5) and GOstats (v.2.60.0)^74–77^. Sequences of the primers used for RT–PCR can be found in Supplementary Table 9.

### mtDNA content

DNA from cells was extracted using all prep DNA/RNA kit (Qiagen #80204) according to the kit protocol. DNA was treated with RNase (Thermo #EN0531), and quantitative PCR was performed using primers for mitochondrial D-loop and mitochondrial genes. mtDNA content was normalized with nuclear DNA content. Sequences of the primers used for the experiment can be found in Supplementary Table 9.

### Seahorse bioenergetic profiling

A total of 10,000 MEFs or 15,000 3T3 fibroblasts were seeded per well of the Seahorse XFe96 96-well assay microplate in the morning and experiments were performed following manufacturers’ recommendations. For cellular respiration Mito Stress Test, medium was replaced in the evening with 180 μl Seahorse medium (Seahorse XF base medium supplemented with 10 mM glucose, 1 mM sodium pyruvate, 2 mM glutamine (Gibco #35050-061) and 5% FCS, pH 7.4) and incubated at 37 °C in a CO_2_-free incubator for 1 h before the assay to avoid natural buffering of the medium. OCR and ECAR were measured over time using sequential injection of oligomycin (1 μM), FCCP (5 μM) and rotenone (1 μM) and antimycin A (1 μM). For glycosis, proton efflux rate was measured over time using sequential injection of rotenone (1 μM) and antimycin A A (1 μM) and 50 mM 2-deoxyglucose. For mtETC complex, activities were performed according to ref. ^50^. Briefly, medium was replaced with an assay buffer and loaded immediately for the assay. To determine complex IV activity, OCR was measured over time using sequential injection of rotenone (1 μM) and antimycin A (1 μM); Plasma Membrane Permeabilzer (from Agilent #102504-100), TMPD (0.5 mM) and ascorbate (2 mM); and potassium azide (20 mM). OCR was baselined to the measurement before TMPD injection to calculate oxygen consumption due to complex IV activity. Pyruvate was used as the substrate for complex I and succinate was used as the substrate for complex II. All chemicals and drugs were purchased from Sigma. Hoechst was added to achieve a final well concentration of 8 μM with the last injection. Data were acquired using Wave Controller Software (2.6.3) and normalized to total protein content measured by Pierce BCA protein assay kit (Thermo #23225) or cell number by automated 4′,6-diamidino-2-phenylindole-stained nuclei counting using BioTek Instruments. For analyses where more than one Seahorse run had to be merged, data are represented as relative value, to compensate for inter-plate variability.

### Fluorescence-activated cell sorting

A total of 0.2 million cells were seeded per well of six-well plates the evening before the experiment. Next day, cells were washed once with PBS and incubated with medium containing the indicated dye (TMRM, MitoSOX, propidium iodide and pSIVA, dissolved in dimethyl sulfoxide) for 30–60 min at 37 °C. Cells were then collected, resuspended in PBS containing 2% FCS and filtered through 0.45 µM nylon mesh and subsequently fluorescence intensity was measured in BD Fortessa cell sorter, using FACS-Diva software. Data were analysed using FlowJo (v.10.7.1) and normalized to the fluorescence intensity of unstained cells. An example gating strategy is shown in Supplementary Fig. 1.

### Cloning

DNA amplification from plasmids and cDNA was performed using Fusion High Fidelity polymerase (Thermo #F530). Restriction digestions (NEB enzymes), phosphorylation (PNK kinase) and dephosphorylation (antarctic phosphatase) were performed wherever required. Ligation was performed using T4 ligase (Roche #04898117001). NEB stable or DH10b was used for transformation of plasmids. Bacteria were grown under appropriate antibiotic selection. Plasmids were sequenced at the in-house sequencing facility. All kits used were purchased from Zymo Research. Oligos harbouring the indicated point mutations were designed using Agilent primer quest tool and reactions were performed using QuikChange Lightning SDM kit (Agilent #210515), according to the kit protocol. Sequences of the primers used for site-directed mutagenesis could be found in Supplementary Table 9.

### Lipidomics

Standard lipidomics analysis was performed as a service provided by Lipotype. Briefly, lipids from frozen cell or mitochondrial pellets were extracted using chloroform and methanol. Lipid class-specific internal standards were spiked-in before extraction for normalization. Mass spectra of lipids were acquired using a hybrid quadrupole/Orbitrap mass spectrometer with an automated nano-flow electrospray ion source in both positive and negative ion mode. Data were analysed using Lipotype proprietary software after data quality verification. Quantile normalization of lipidomic data was carried out in R using the R package ‘preprocessCore’ (Bolstad, 2022 10.18129/B9.bioc.preprocessCore).

### Mitochondrial proteomics

SILAC was performed as previously described^78^. Cells were cultured in DMEM-SILAC medium with 1% final concentration of MEM (non-essential amino acids solution; Thermo Fisher) lacking arginine and lysine, supplemented with l-arginine-^13^C_6_^15^N_4_ Arg 10 and l-lysine-^13^C_6_^15^N_2_ Lys 8. Control cells were grown in the same medium supplemented with unlabelled amino acids. Labelling was started from an unlabelled culture with less than 1% of the final cell number. Cells were allowed to cycle at least five cell divisions before further expansion. Dishes with 90–100% confluent cells were washed once with PBS and then collected in PBS by scraping gently. Cellular pellets were collected by spinning, washed once with PBS and snap frozen in liquid nitrogen. Pellets were thawed on ice and resuspended in pre-chilled MIB (250 mM sucrose, 1 mM EDTA–NaOH pH 8 and 10 mM MOPS–KOH pH 7.2), supplemented with 1 mM phenylmethylsulfonyl fluoride, 10 mM nicotinamide and 10 µM Trichostatin A. Cells were broken by douncing 30 times using a Teflon pestle. Unbroken cells and nuclei were pelleted at 1,000*g* for 5 min. The supernatant was collected and remaining nuclei were pelleted at 1,000*g* for 10 min. The supernatant was saved for high-speed centrifugation, and the pellet was resuspended in MIB for a second round of douncing. The pooled supernatant from the two rounds of douncing was pelleted at 13,000*g* for 15 min to obtain crude mitochondria. The crude mitochondrial pellet was washed twice with MIB, resuspended in appropriate volume of MIB and its protein concentration was measured. Pellets were resuspended in 8 M urea, 50 mM ammonium bicarbonate and the protein concentration was determined once more using Bradford assay. From each WT and MOF KO sample, an aliquot containing 10 µg protein was diluted to 1 mg ml^−1^ in urea buffer, mixed in a 1:1 ratio and proteins were reduced using 5 mM tris(2-carboxyethyl)phosphine for 30 min at 37 °C and free cysteines alkylated using 50 mM iodoacetamide for 30 min in the dark. The urea concentration was diluted to 1.6 M using 50 mM ammonium bicarbonate, and trypsin (Promega) was added 1:50 (w/w) for digestion overnight at 37 °C. Tryptic digests were acidified using trichloroacetic acid to a final concentration of 1 %, centrifuged for 10 min at 2,000*g* and room temperature and subsequently fractionated using StageTips^79^. For this, four layers of C18 material were punched from extraction disc and conditioned once with 100% methanol, once with 80% (v/v) acetonitrile (CAN), 0.5 % (v/v) acetic acid (AA) and twice with 0.5% (v/v) AA. Peptides were loaded, washed twice with 0.5% (v/v) AA and sequentially eluted with 0%, 2.7%, 4.5%, 7.2%, 10.8%, 14.4%, 18% and 64.8% acetonitrile (v/v) in 10 mM NH_4_OH. Fractions were dried in vacuo and desalted using StageTips, and dried peptides were stored at −80 °C until liquid chromatography–mass spectrometry (LC–MS) analysis.

### LC–MS

Reversed-phase LC–MS was performed using a UltiMate 3000 RSLCnano system (Dionex LC Packings/Thermo Fisher Scientific) equipped with two C18 pre-columns (nanoEase M/Z Symmetry C18, 180 µm × 20 mm,100 Å, 5 µm, Waters) and a C18 main column (nanoEase MZ HSS C18 T3 Col, 75 µm × 250 mm, 100 Å, 2 µM, Waters). The UHPLC system was coupled online to a Q Exactive Plus instrument (Thermo Fisher Scientific) equipped with a nanoelectrospray ion source and a fused silica emitter (New Objectives). For MS analysis, dried peptides were resuspended in 0.1% trifluoroacetic acid and analysed using a 2 h LC gradient. Gradients were generated using binary solvent systems of 0.1% formic acid (v/v, solvent A) and 0.1% formic acid/86% acetonitrile (v/v, solvent B). A gradient of 4–20% B in 60 min was applied followed by an increase of B to 54% in 35 min and to 95% in 5 min. The column was re-equilibrated for 16 min at 4% solvent B. Full scans (*m*/*z* 375–1,700) were acquired with a mass resolution of 70,000 at *m*/*z* 200. Automatic gain control was set to 3 × 10^6^ with a maximum ion time of 60 ms. MS/MS analysis was performed on multiply charged ions using a normalized collision energy of 28% with higher-energy collisional dissociation and an exclusion time of 45 s. Automatic gain control for MS/MS scans was 1 × 10^5^, with a resolution of 35,000 and a maximum ion time of 120 ms.

### Data analysis

MS data were analysed using MaxQuant 1.5.3.30 and 1.6.0.1 to search peak list against the UniProt ProteomeSet mouse database (release 11.2018, 62,407 protein entries) and a list of common contaminants provided by MaxQuant using Andromeda^74,75^. The precursor mass tolerance was set to 20 ppm for the first search and to 4.5 ppm for the main search. Arg10/Lys8 were set as ‘heavy’ labels, and multiplicity was set to 2. Trypsin was set as fixed modification and methionine oxidation and acetylation of the N-terminus as variable modifications. Proteins were quantified on the basis of more than or equal to 1 unique peptide with a length of more than or equal to seven residues. The options ‘re-quantify’ and ‘match between runs’ were enabled. A false discovery rate of 1% for peptide spectrum match was applied using the decoy mode ‘Revert’.

### Statistics and reproducibility

For experiments involving primary MEFs, individual embryos were considered as a biological replicate. For experiments involving cell lines, independent experiments performed on different days and different passage numbers were considered as biological replicates. Statistical significance and parameters used are reported in respective figures and legends. All data are plotted as mean ± standard error of the mean (s.e.m.), unless mentioned otherwise. Data points were first tested for Gaussian distribution, and parametric or non-parametric tests were applied accordingly. All statistical tests were done using GraphPad PRISM 9, unless mentioned otherwise. For qualitative analyses of immunoblots, images are representative of more than three independent experiments.

### Reporting summary

Further information on research design is available in the Nature Portfolio Reporting Summary linked to this article.

### Supplementary information

Supplementary Information List of Supplementary Movies 1 and 2, Tables 1–8 and Fig. 1.

Reporting Summary

Supplementary Movie 1 Related to Fig. 1e, live-cell imaging of mitochondrial structure in MOF-iWT.

Supplementary Movie 2 Related to Fig. 1e, live-cell imaging of mitochondrial structure in MOF-iKO (S2) cells.

Supplementary Tables 1–8 Supplementary Tables 1–8.

### Source data

Source Data Fig. 1 Statistical source data.

Source Data Fig. 2 Statistical source data.

Source Data Fig. 3 Statistical source data.

Source Data Fig. 4 Statistical source data.

Source Data Fig. 5 Statistical source data.

Source Data Fig. 6 Statistical source data.

Source Data Fig. 7 Statistical source data.

Source Data Extended Data Fig. 1 Statistical source data.

Source Data Extended Data Fig. 2 Statistical source data.

Source Data Extended Data Fig. 3 Statistical source data.

Source Data Extended Data Fig. 5 Statistical source data.

Source Data Extended Data Fig. 6 Statistical source data.

Source Data Extended Data Fig. 7 Statistical source data.

Source Data Fig. 1 Unprocessed western blots.

Source Data Fig. 2 Unprocessed western blots.

Source Data Fig. 3 Unprocessed western blots.

Source Data Fig. 4 Unprocessed western blots.

Source Data Fig. 5 Unprocessed western blots.

Source Data Fig. 6 Unprocessed western blots.

Source Data Fig. 7 Unprocessed western blots.

Source Data Extended Data Fig. 1 Unprocessed western blots.

Source Data Extended Data Fig. 2 Unprocessed western blots.

Source Data Extended Data Fig. 4 Unprocessed western blots.

Source Data Extended Data Fig. 5 Unprocessed western blots.

Source Data Extended Data Fig. 6 Unprocessed western blots.

## Data Availability

All the raw RNA-seq datasets and differential expression analysis tables generated in this study have been deposited to the Gene Expression Omnibus (accession ID GSE199009). The KANSL2 acetylome and proteomics data in MEFs have been deposited to the ProteomeXchange consortium via the PRIDE partner repository (accession ID PXD038521). The MOF acetylome data were re-analysed from published work (accession ID PXD008539) (Karoutas et al., 2019). R scripts and codes used for the analysis of next-generation sequencing datasets are available upon request. Source data are provided with this paper.

## Code availability

R scripts and codes used for the analysis of NGS datasets are available upon request.
