# Supplementary material for: COX17 acetylation via MOF–KANSL complex promotes mitochondrial integrity and function
Source: Nat Metab. 2023 Oct 9;5(11):1931–52. doi: 10.1038/s42255-023-00904-w (PMC10663164; doi:10.1038/s42255-023-00904-w)

nature metabolism

Article

https://doi.org/10.1038/s42255-023-00904-w

COX17 acetylation via MOF–KANSL complex promotes mitochondrial integrity and function

In the format provided by the authors and unedited

Supplementary Information

Movies S1,S2: Related to **Figure 1e**, Live-cell imaging of mitochondrial structure in MOF-iWT (S1) and MOF-iKO (S2) cells

Table S1: Lipidomics analysis of COX17 knockdown, MOF-iWT and MOF-iKO MEFs from whole cell and mitochondrial extracts

Table S2: Summary of significantly downregulated mitochondrial genes from total RNA-Seq of MOF-iWT and MOF-iKO MEFs

Table S3: Summary of all significantly downregulated genes from total RNA-Seq of MOF- iWT and MOF-iKO MEFs

Table S4: Summary of the transcripts encoding for MitoCarta 3.0 proteins and mildly downregulated upon loss of MOF under both glucose and galactose conditions

Table S5: Summary of the transcripts significantly up- or downregulated upon MOF KO under galactose conditions

Table S6: Mitochondrial protein abundances upon MOF depletion in primary MEFs

Table S7: Mitochondrial acetylation targets of MOF

Table S8: Multiple comparison statistics of data in **Figure 4a**

Supplementary Figure S1: Example of the gating strategy used to identify live and single iWT and iKO MEFs

Supplementary Figure S1

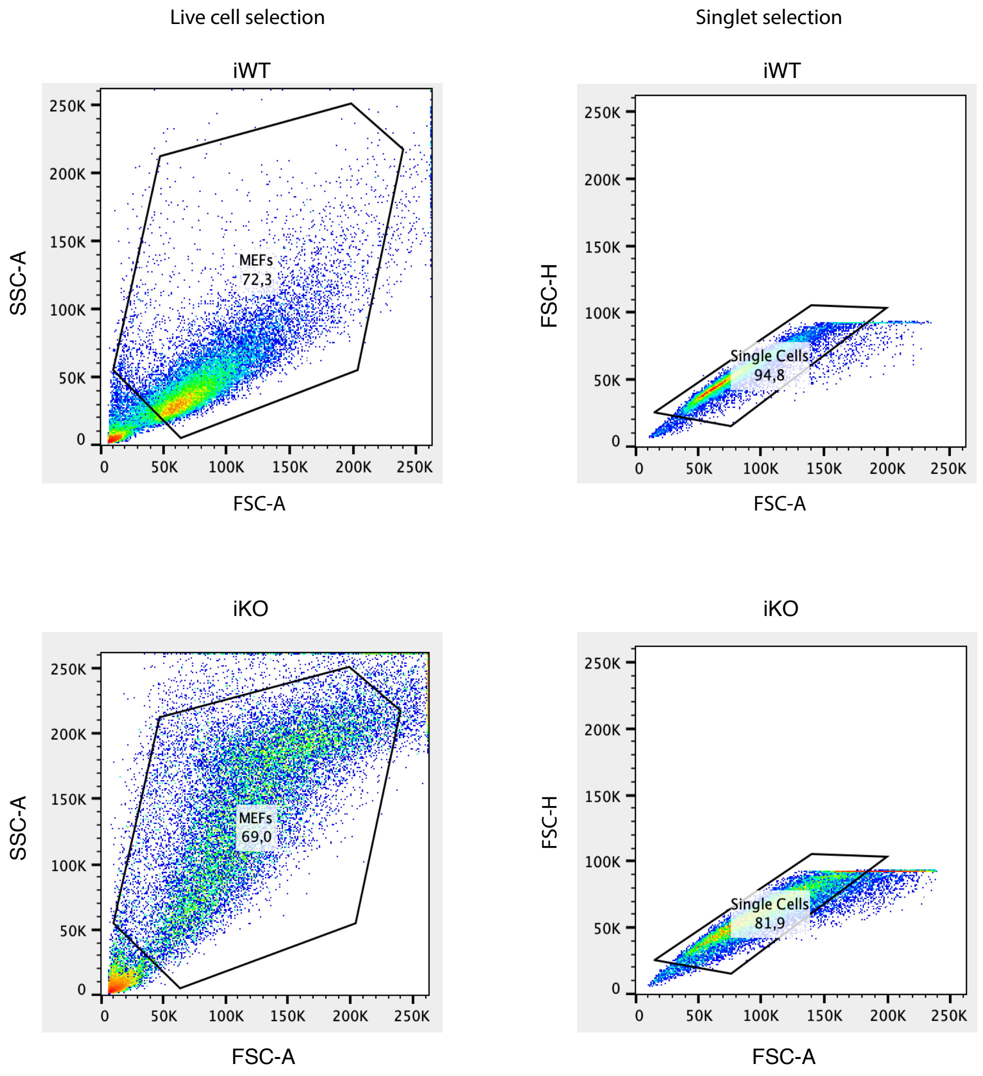

Supplement: Supplementary file 1 — List of Supplementary Movies 1 and 2, Tables 1–8 and Fig. 1. [file 42255_2023_904_MOESM1_ESM.pdf]
