# Supplementary material for: COX17 acetylation via MOF–KANSL complex promotes mitochondrial integrity and function
Source: Nat Metab. 2023 Oct 9;5(11):1931–52. doi: 10.1038/s42255-023-00904-w (PMC10663164; doi:10.1038/s42255-023-00904-w)
Supplement: Supplementary file 2 — Reporting Summary [file 42255_2023_904_MOESM2_ESM.pdf]

nature portfolio

Corresponding author(s): Asifa Akhtar

Last updated by author(s): Aug 28, 2023

Reporting Summary

Nature Portfolio wishes to improve the reproducibility of the work that we publish. This form provides structure for consistency and transparency in reporting. For further information on Nature Portfolio policies, see our Editorial Policies and the Editorial Policy Checklist.

Statistics

For all statistical analyses, confirm that the following items are present in the figure legend, table legend, main text, or Methods section.

n/a	Confirmed	
☐	☒	The exact sample size (*n*) for each experimental group/condition, given as a discrete number and unit of measurement
☐	☒	A statement on whether measurements were taken from distinct samples or whether the same sample was measured repeatedly
☐	☒	The statistical test(s) used AND whether they are one- or two-sided<br>*Only common tests should be described solely by name; describe more complex techniques in the Methods section.*
☐	☒	A description of all covariates tested
☐	☒	A description of any assumptions or corrections, such as tests of normality and adjustment for multiple comparisons
☐	☒	A full description of the statistical parameters including central tendency (e.g. means) or other basic estimates (e.g. regression coefficient) AND variation (e.g. standard deviation) or associated estimates of uncertainty (e.g. confidence intervals)
☐	☒	For null hypothesis testing, the test statistic (e.g. *F*, *t*, *r*) with confidence intervals, effect sizes, degrees of freedom and *P* value noted<br>*Give P values as exact values whenever suitable.*
☒	☐	For Bayesian analysis, information on the choice of priors and Markov chain Monte Carlo settings
☐	☒	For hierarchical and complex designs, identification of the appropriate level for tests and full reporting of outcomes
☒	☐	Estimates of effect sizes (e.g. Cohen's *d*, Pearson's *r*), indicating how they were calculated

Our web collection on statistics for biologists contains articles on many of the points above.

Software and code

Policy information about availability of computer code

Data collection	Zen2 Blue Software (version 3.1 and 3.2), Zen Black Software (version 2012, Service pack 5), Wave Controller Software (2.6.3)
Data analysis	Statistical analysis: GraphPad Prism (version 9),<br>RNA-Seq analysis: SnakePipes (v.2.5.1), subread package (v.2.0.0), RStudio (v.4.1.1), featureCounts (v.2.0.0), R packages DESeq2(v.1.34.0), ggplot2(v.3.3.5), GOstats (v.2.60.0),<br>Flow cytometry analysis: FlowJo (v.10.7.1)<br>Fluorescence and EM image analysis: Fiji is Just ImajeJ (version 1.0)<br>Mass-spectrometry data analysis: MaxQuant software (version 1.5.3.30; 1.6.0.1)

For manuscripts utilizing custom algorithms or software that are central to the research but not yet described in published literature, software must be made available to editors and reviewers. We strongly encourage code deposition in a community repository (e.g. GitHub). See the Nature Portfolio guidelines for submitting code & software for further information.

Data

Policy information about availability of data

All manuscripts must include a data availability statement. This statement should provide the following information, where applicable:

- Accession codes, unique identifiers, or web links for publicly available datasets
- A description of any restrictions on data availability
- For clinical datasets or third party data, please ensure that the statement adheres to our policy

The raw and processed files for RNA-Seq experiments have been deposited to Gene Expression Omnibus with accession code GSE199009. The KANSL2 acetylome and proteomics data in MEFs has been deposited to the ProteomeXchange consortium via the PRIDE partner repository (accession ID: PXD038521). The MOF

acetylome data was reanalyzed from published work (accession ID: PXD008539) (Karoutas et al., 2019). The MS proteomics data for mitochondrial proteome has been deposited to the ProteomeXchange consortium via the PRIDE partner repository and will be made available upon publication. All other data supporting the findings of this study are available along with the manuscript or would be possible to obtain from the corresponding author upon reasonable request.

Field-specific reporting

Please select the one below that is the best fit for your research. If you are not sure, read the appropriate sections before making your selection.

☒ Life sciences ☐ Behavioural & social sciences ☐ Ecological, evolutionary & environmental sciences

For a reference copy of the document with all sections, see nature.com/documents/nr-reporting-summary-flat.pdf

Life sciences study design

All studies must disclose on these points even when the disclosure is negative.

Sample size	No statistical methods were used to predetermine sample sizes, but are similar to those reported in previous publications: doi: 10.1016/j.cell.2016.09.052, doi: 10.1038/s41556-019-0397-z, doi: 10.1038/s41556-020-0526-8.
Data exclusions	No data were excluded from the analyses performed in this paper.
Replication	Reproducibility of all experiments in this manuscript were verified by using n>2 biologically independent experiments, either in the same experimental graph or across multiple experiments throughout the manuscript. For experiments involving primary MEFs, individual embryos were considered as a biological replicate. For experiments involving cell lines, independent experiments performed on different days and different passage numbers were considered as biological replicates. Statistical significance and parameters used are reported in respective figures and legends. All attempts at replication were successful.
Randomization	Samples were allocated into experimental groups randomly and independent of sex or cell passage
Blinding	The electron microscopists, mass spectrometrists and Deep-Seq facility technicians were blinded to experimental conditions. Sample randomization during data acquisition justified unblinded sample preparation by the investigators.

Behavioural & social sciences study design

All studies must disclose on these points even when the disclosure is negative.

Study description	*Briefly describe the study type including whether data are quantitative, qualitative, or mixed-methods (e.g. qualitative cross-sectional, quantitative experimental, mixed-methods case study).*
Research sample	*State the research sample (e.g. Harvard university undergraduates, villagers in rural India) and provide relevant demographic information (e.g. age, sex) and indicate whether the sample is representative. Provide a rationale for the study sample chosen. For studies involving existing datasets, please describe the dataset and source.*
Sampling strategy	*Describe the sampling procedure (e.g. random, snowball, stratified, convenience). Describe the statistical methods that were used to predetermine sample size OR if no sample-size calculation was performed, describe how sample sizes were chosen and provide a rationale for why these sample sizes are sufficient. For qualitative data, please indicate whether data saturation was considered, and what criteria were used to decide that no further sampling was needed.*
Data collection	*Provide details about the data collection procedure, including the instruments or devices used to record the data (e.g. pen and paper, computer, eye tracker, video or audio equipment) whether anyone was present besides the participant(s) and the researcher, and whether the researcher was blind to experimental condition and/or the study hypothesis during data collection.*
Timing	*Indicate the start and stop dates of data collection. If there is a gap between collection periods, state the dates for each sample cohort.*
Data exclusions	*If no data were excluded from the analyses, state so OR if data were excluded, provide the exact number of exclusions and the rationale behind them, indicating whether exclusion criteria were pre-established.*
Non-participation	*State how many participants dropped out/declined participation and the reason(s) given OR provide response rate OR state that no participants dropped out/declined participation.*
Randomization	*If participants were not allocated into experimental groups, state so OR describe how participants were allocated to groups, and if allocation was not random, describe how covariates were controlled.*

March 2021

Ecological, evolutionary & environmental sciences study design

All studies must disclose on these points even when the disclosure is negative.

Study description	*Briefly describe the study. For quantitative data include treatment factors and interactions, design structure (e.g. factorial, nested, hierarchical), nature and number of experimental units and replicates.*
Research sample	*Describe the research sample (e.g. a group of tagged Passer domesticus, all Stenocereus thurberi within Organ Pipe Cactus National Monument), and provide a rationale for the sample choice. When relevant, describe the organism taxa, source, sex, age range and any manipulations. State what population the sample is meant to represent when applicable. For studies involving existing datasets, describe the data and its source.*
Sampling strategy	*Note the sampling procedure. Describe the statistical methods that were used to predetermine sample size OR if no sample-size calculation was performed, describe how sample sizes were chosen and provide a rationale for why these sample sizes are sufficient.*
Data collection	*Describe the data collection procedure, including who recorded the data and how.*
Timing and spatial scale	*Indicate the start and stop dates of data collection, noting the frequency and periodicity of sampling and providing a rationale for these choices. If there is a gap between collection periods, state the dates for each sample cohort. Specify the spatial scale from which the data are taken*
Data exclusions	*If no data were excluded from the analyses, state so OR if data were excluded, describe the exclusions and the rationale behind them, indicating whether exclusion criteria were pre-established.*
Reproducibility	*Describe the measures taken to verify the reproducibility of experimental findings. For each experiment, note whether any attempts to repeat the experiment failed OR state that all attempts to repeat the experiment were successful.*
Randomization	*Describe how samples/organisms/participants were allocated into groups. If allocation was not random, describe how covariates were controlled. If this is not relevant to your study, explain why.*
Blinding	*Describe the extent of blinding used during data acquisition and analysis. If blinding was not possible, describe why OR explain why blinding was not relevant to your study.*

Did the study involve field work? ☐ Yes ☐ No

Field work, collection and transport

Field conditions	*Describe the study conditions for field work, providing relevant parameters (e.g. temperature, rainfall).*
Location	*State the location of the sampling or experiment, providing relevant parameters (e.g. latitude and longitude, elevation, water depth).*
Access & import/export	*Describe the efforts you have made to access habitats and to collect and import/export your samples in a responsible manner and in compliance with local, national and international laws, noting any permits that were obtained (give the name of the issuing authority, the date of issue, and any identifying information).*
Disturbance	*Describe any disturbance caused by the study and how it was minimized.*

Reporting for specific materials, systems and methods

We require information from authors about some types of materials, experimental systems and methods used in many studies. Here, indicate whether each material, system or method listed is relevant to your study. If you are not sure if a list item applies to your research, read the appropriate section before selecting a response.

Materials & experimental systems

n/a	Involved in the study	
☐	☒	Antibodies
☐	☒	Eukaryotic cell lines
☒	☐	Palaeontology and archaeology
☐	☒	Animals and other organisms
☒	☐	Human research participants
☒	☐	Clinical data
☒	☐	Dual use research of concern

Methods

n/a	Involved in the study	
☒	☐	ChIP-seq
☐	☒	Flow cytometry
☒	☐	MRI-based neuroimaging

Antibodies

Antibodies used

ATP Synthase (WB 1:1000 dilution), Abcam Cat#ab109798
COX17 (WB 1:500 dilution, IF 1:250 dilution), Santa Cruz (Clone F14) Cat#sc-100521
Cytochrome C (WB 1:1000 dilution), BD Pharmigen Cat#556433
DRP1 (WB 1:1000 dilution), Cell Signaling Technology Cat#8570
FLAG (IF 1:500 dilution), Sigma Aldrich Cat#1804
GRP75 (WB 1:1000 dilution), Santa Cruz (Clone D9) Cat#sc-133137
H4K16Ac (WB 1:1000 dilution), Millipore Cat#07329
HSP60 (WB 1:1000 dilution), Cell Signaling Technology (Clone D6F1) XP(R) Cat#12165S
KANSL1 (WB 1:500 dilution), Abnova Cat#PAB20355
KANSL2 (WB 1:500 dilution), Sigma Aldrich Cat#HPA038498
KANSL3 (WB 1:500 dilution), Sigma Aldrich Cat#HPA035018
MOF (WB 1:500 dilution), Bethyl Cat#A300-992A
MOF (WB 1:1000 dilution), Abcam Cat#EPR15803, ab200660
MOF (WB 1:1000 dilution), this paper
MFN1 (WB 1:1000 dilution), Abcam Cat#ab126575
MFN2 (WB 1:1000 dilution), Cell Signaling Technology Cat#9482S
OPA1 (WB 1:1000 dilution), BD Pharmigen Cat#612606
Pan-acetyl Lysine (WB 1:500 dilution), Cell Signaling Technology Cat#9441
TIM23 (WB 1:1000 dilution), BD Transduction Laboratories Cat#611223
TOM20 (WB 1:1000 dilution, IF 1:500 dilution), Santa Cruz Cat#sc-11415
Total OXPHOS Rodent WB Antibody Cocktail (WB 1:1000 dilution), Abcam Cat#ab110413

HRP-conjugated antibodies:
ß-Actin-HRP (WB 1:5000 dilution), Santa Cruz Cat#sc-47778
FLAG-HRP (WB 1:5000 dilution), Sigma Aldrich Cat#A8592
GAPDH-HRP (WB 1:1000 dilution), ThermoFischer#MA5-15738-HRP
H3-HRP (WB 1:1000 dilution), Cell Signaling Technology (Clone D1H2) XP Cat#12648S
H4-HRP (WB 1:1000 dilution), Abcam Cat#ab197517
V5-HRP (WB 1:1000 dilution), Invitrogen Cat#46-0708

NDUFA9 (WB 1:250 dilution), RISP (WB 1:250 dilution), COX1 (WB 1:250 dilution), SDHA (WB 1:500 dilution), ATP5A (1:200 dilution), COX4.1 (WB 1:200 dilution) , COX5A (WB 1:200 dilution) and HSP60 (WB 1:500 dilution), kind gift from N.Pfanner

Goat anti-Rabbit IgG H&L (IF 1:500 dilution), Alexa Fluor® 488 Cat#A11034
Goat anti-Rabbit IgG H&L (IF 1:500 dilution), Alexa Fluor® 594 Cat#A21429
Goat anti-Mouse IgG H&L (IF 1:500 dilution), Alexa Fluor® 488 Cat#A11029
Goat anti-Mouse IgG H&L (IF 1:500 dilution), Alexa Fluor® 555 Cat#aA21422

Validation

ß-Actin-HRP (Santa Cruz Cat#sc-47778), Cytochrome C (BD Pharmigen Cat#556433), GRP75 (Santa Cruz (Clone D9) Cat#sc-133137), NDUFA9, RISP, COX1, SDHA, ATP5A, COX4.1, COX5A and HSP60 (kind gift from N.Pfanner) are controlled by comparing total cell extracts and mitochondrial extracts validated in this study through WB of cellular fractions of MEFs. COX17 (Santa Cruz Cat#sc-100521), H4K16Ac (Millipore Cat#07329), KANSL2 (Sigma Aldrich Cat#HPA038498), KANSL3 (Sigma Aldrich Cat#HPA035018) and MOF (Bethyl Cat#A300-992A; Abcam Cat#EPR15803; ab200660, this paper) are validated in this study through WBs of the respective knockout and/or knockdown systems in MEFs and/or 3T3 fibroblasts. FLAG (Sigma Aldrich Cat#1804), FLAG-HRP (Sigma Aldrich Cat#A8592) and V5-HRP (Invitrogen Cat#46-0708) are validated in this study through WBs and/IFs of the respective ectopic or exogenous or overexpression in MEFs and/or 3T3 fibroblasts.

ATP Synthase (Abcam Cat#ab109798) is referenced by the manufacturer (hhttps://www.abcam.com/products/primary-antibodies/complex-i-immunocapture-antibody-18g12bc2-ab109798.html)
Cytochrome C (BD Pharmigen Cat#556433) is validated using knockout cellular system in Kalpage HA. et al., 2020
DRP1 (Cell Signaling Technology Cat#8570) is validated using knockdown system in Wang Y. et al., 2020
GAPDH-HRP (ThermoFischer#MA5-15738-HRP) has been extensively used in publications referred to by the manufacturer(https://www.bdbiosciences.com/en-us/products/reagents/microscopy-imaging-reagents/immunofluorescence-reagents/purified-mouse-anti-tim23.611223)
H4-HRP (Abcam Cat#ab197517) is validated by the manufacturere by using recombinant proteins (https://www.abcam.com/hrp-histone-h4-antibody-mabcam-31830-ab197517.html)
H4K16Ac (Millipore Cat#07329) is validated using human dermal fibroblasts in Basilicata MF. et al., 2018
HSP60 (Cell Signaling Technology Clone D6F1) XP(R) Cat#12165S) (https://www.cellsignal.com/products/primary-antibodies/hsp60-d6f1-xp-rabbit-mab/12165?_requestid=2900254)
KANSL1 (Abnova Cat#PAB20355) is validated using siKANSL1 HeLa cells in Chatterjee A. et al., 2016
OPA1 (BD Pharmigen Cat#612606) is validated in multiple publications (https://www.bdbiosciences.com/en-de/products/reagents/microscopy-imaging-reagents/immunofluorescence-reagents/purified-mouse-anti-opa1.612606)
MFN1 (Abcam Cat#ab126575) is validated by the manufacturer using a wildtype and MFN1 deficient MEFs (https://www.abcam.com/mitofusin-1-antibody-11e91h12-ab126575.html)
MFN2 (Cell Signaling Technology Cat#9482S) and OPA1 (BD Pharmigen Cat#612606) are validated using knockout mouse models in Buck MD. et al, 2016
Pan-acetyl Lysine (Cell Signaling Technology Cat#9441) is extensively characterized by the manufacturer (https://www.cellsignal.com/products/primary-antibodies/acetylated-lysine-antibody/9441)
TIM23 (BD Transduction Laboratories Cat#611223) is well referenced by the manufacturer (https://www.bdbiosciences.com/en-de/products/reagents/microscopy-imaging-reagents/immunofluorescence-reagents/purified-mouse-anti-tim23.611223)
TOM20 (Santa Cruz Cat#sc-11415) is extensively characterized by the manufacturer using imaging and WB (https://www.scbt.com/p/tom20-antibody-fl-145)

	Total OXPHOS Rodent WB Antibody Cocktail is guaranteed by the manufacturer (Abcam) for WB detection of OXPHOS protein complexes

Eukaryotic cell lines

Policy information about cell lines

Cell line source(s)	Primary Mouse Embryonic Fibroblasts (MEFs) were derived from embryonic day (E)13.5 Mof fl/fl or Mof fl/fl;Cre-ERT2T/+ embryos. Kansl2 and Kansl3 MEFs were generated in a similar fashion.<br>Flip-In 3T3 fibroblast cell line was purchased from Thermo Fisher.<br>HeLa Flp-In TRex cell line was a kind gift from Prof. Stephen Taylor, University of Manchester<br>HEK293T cells were obtained from BIOSS, University of Freiburg<br>Primary Human Dermal Fibroblasts (HDFs) derived from skin punches of individuals and were a gift from P. M. Campeau, H. Kingston, P. B. Agarwal and J. M. van Hagen.<br>BJ cells were obtained from ATCC #CRL-2522.
Authentication	Verification knockout, knockdown, ectopic expression and over-expression in the relevant cell line validated with qPCR and/or Western blot analysis experiments, that are included in this paper.
Mycoplasma contamination	All cell lines used in this study were tested negative for mycoplasma.
Commonly misidentified lines<br>(See ICLAC register)	No cell lines used in this study were found in the database of commonly misidentified cell lines that is maintained by ICLAC.

Palaeontology and Archaeology

Specimen provenance	*Provide provenance information for specimens and describe permits that were obtained for the work (including the name of the issuing authority, the date of issue, and any identifying information). Permits should encompass collection and, where applicable, export.*
Specimen deposition	*Indicate where the specimens have been deposited to permit free access by other researchers.*
Dating methods	*If new dates are provided, describe how they were obtained (e.g. collection, storage, sample pretreatment and measurement), where they were obtained (i.e. lab name), the calibration program and the protocol for quality assurance OR state that no new dates are provided.*

☐ Tick this box to confirm that the raw and calibrated dates are available in the paper or in Supplementary Information.

Ethics oversight	*Identify the organization(s) that approved or provided guidance on the study protocol, OR state that no ethical approval or guidance was required and explain why not.*

Note that full information on the approval of the study protocol must also be provided in the manuscript.

Animals and other organisms

Policy information about studies involving animals; ARRIVE guidelines recommended for reporting animal research

Laboratory animals	No experiments with animal subjects for treatments were used this manuscripts. Animals were used to derive primary fibroblasts in accordance with the German animal care and ethics legislation. All animals were maintained on a pure C57BL/6 background, under a 12-hour light and dark cycle. Water and standard chow were provided adlibitum. MEFs were derived from embryonic day (E)13.5 Mof fl/fl, Mof fl/fl;Cre-ERT2T/+, Kansl2 fl/fl, Kansl2 fl/fl;Cre-ERT2T/+, Kansl3 fl/fl and Kansl3 fl/fl;Cre-ERT2T/+ embryos from 7-12 week old pregnant females. Males and females embryos were used in a similar ratio for each experiment to account for possible sex-dependent phenotypic variation.
Wild animals	No wild animals were used in this study.
Field-collected samples	Field-collected samples were not used in this study.
Ethics oversight	All experiments involving animals were performed according to the German animal care and ethics legislation. Furthermore, the protocols applied have been evaluated and approved by the local government authorities, the Committee on Research Animal Care and the Regierungspräsidium Freiburg. This project was performed according to the anzeigepflichtiges Versuchsvorhaben (notifiable experimental project) with the relevant license “Akh-iTo-2” (Toetung ohne Vorbehandlung) and approved by the Max Planck Institute of Immunobiology and Epigenetics, welfare officer Dr. Stefanie Kunz.

Note that full information on the approval of the study protocol must also be provided in the manuscript.

Human research participants

Policy information about studies involving human research participants

Population characteristics	*Describe the covariate-relevant population characteristics of the human research participants (e.g. age, gender, genotypic information, past and current diagnosis and treatment categories). If you filled out the behavioural & social sciences study design questions and have nothing to add here, write "See above."*

Recruitment	*Describe how participants were recruited. Outline any potential self-selection bias or other biases that may be present and how these are likely to impact results.*
Ethics oversight	*Identify the organization(s) that approved the study protocol.*

Note that full information on the approval of the study protocol must also be provided in the manuscript.

Clinical data

Policy information about clinical studies

All manuscripts should comply with the ICMJE guidelines for publication of clinical research and a completed CONSORT checklist must be included with all submissions.

Clinical trial registration	*Provide the trial registration number from ClinicalTrials.gov or an equivalent agency.*
Study protocol	*Note where the full trial protocol can be accessed OR if not available, explain why.*
Data collection	*Describe the settings and locales of data collection, noting the time periods of recruitment and data collection.*
Outcomes	*Describe how you pre-defined primary and secondary outcome measures and how you assessed these measures.*

Dual use research of concern

Policy information about dual use research of concern

Hazards

Could the accidental, deliberate or reckless misuse of agents or technologies generated in the work, or the application of information presented in the manuscript, pose a threat to:

No	Yes	
☐	☐	Public health
☐	☐	National security
☐	☐	Crops and/or livestock
☐	☐	Ecosystems
☐	☐	Any other significant area

Experiments of concern

Does the work involve any of these experiments of concern:

No	Yes	
☐	☐	Demonstrate how to render a vaccine ineffective
☐	☐	Confer resistance to therapeutically useful antibiotics or antiviral agents
☐	☐	Enhance the virulence of a pathogen or render a nonpathogen virulent
☐	☐	Increase transmissibility of a pathogen
☐	☐	Alter the host range of a pathogen
☐	☐	Enable evasion of diagnostic/detection modalities
☐	☐	Enable the weaponization of a biological agent or toxin
☐	☐	Any other potentially harmful combination of experiments and agents

ChIP-seq

Data deposition

☐ Confirm that both raw and final processed data have been deposited in a public database such as GEO.

☐ Confirm that you have deposited or provided access to graph files (e.g. BED files) for the called peaks.

Data access links<br>*May remain private before publication.*	*For "Initial submission" or "Revised version" documents, provide reviewer access links. For your "Final submission" document, provide a link to the deposited data.*
Files in database submission	*Provide a list of all files available in the database submission.*
Genome browser session<br>(e.g. UCSC)	*Provide a link to an anonymized genome browser session for "Initial submission" and "Revised version" documents only, to enable peer review. Write "no longer applicable" for "Final submission" documents.*

Methodology

Replicates	*Describe the experimental replicates, specifying number, type and replicate agreement.*
Sequencing depth	*Describe the sequencing depth for each experiment, providing the total number of reads, uniquely mapped reads, length of reads and whether they were paired- or single-end.*
Antibodies	*Describe the antibodies used for the ChIP-seq experiments; as applicable, provide supplier name, catalog number, clone name, and lot number.*
Peak calling parameters	*Specify the command line program and parameters used for read mapping and peak calling, including the ChIP, control and index files used.*
Data quality	*Describe the methods used to ensure data quality in full detail, including how many peaks are at FDR 5% and above 5-fold enrichment.*
Software	*Describe the software used to collect and analyze the ChIP-seq data. For custom code that has been deposited into a community repository, provide accession details.*

Flow Cytometry

Plots

Confirm that:

☒ The axis labels state the marker and fluorochrome used (e.g. CD4-FITC).

☒ The axis scales are clearly visible. Include numbers along axes only for bottom left plot of group (a 'group' is an analysis of identical markers).

☒ All plots are contour plots with outliers or pseudocolor plots.

☒ A numerical value for number of cells or percentage (with statistics) is provided.

Methodology

Sample preparation	0.2 million cells were seeded per well of 6-well plates the evening before the experiment. Next day, cells were washed once with PBS and incubated with media containing the indicated dye (TMRM, MitoSOX, mitotacker, dissolved in DMSO) for 30-60 minutes at 37°C. Cells were then harvested, resuspended in PBS containing 2% FCS and filtered through 0.45 uM nylon mesh.
Instrument	Data were collected with BD Fortessa cell sorter,
Software	Data were collected using BD FACSDiva Software.<br>Data were analyzed using FlowJo V10 Software.
Cell population abundance	No sorting was performed.
Gating strategy	Live cells were identified and debris was excluded using FSC/SSC. Doublets were excluded using FSC-H/FSC-W. Mitochondrial ROS production and membrane potential were detected using MitoSOX Red and TMRM, respectively (both from ThermoFischer). Cell cycle and apoptosis analysis was performed using propidium iodide and pSIVA (Novus Biologicals), respectively.

☒ Tick this box to confirm that a figure exemplifying the gating strategy is provided in the Supplementary Information.

Magnetic resonance imaging

Experimental design

Design type	*Indicate task or resting state; event-related or block design.*
Design specifications	*Specify the number of blocks, trials or experimental units per session and/or subject, and specify the length of each trial or block (if trials are blocked) and interval between trials.*
Behavioral performance measures	*State number and/or type of variables recorded (e.g. correct button press, response time) and what statistics were used to establish that the subjects were performing the task as expected (e.g. mean, range, and/or standard deviation across subjects).*

Acquisition

Imaging type(s) | *Specify: functional, structural, diffusion, perfusion.*

Field strength | *Specify in Tesla*

Sequence & imaging parameters | *Specify the pulse sequence type (gradient echo, spin echo, etc.), imaging type (EPI, spiral, etc.), field of view, matrix size, slice thickness, orientation and TE/TR/flip angle.*

Area of acquisition | *State whether a whole brain scan was used OR define the area of acquisition, describing how the region was determined.*

Diffusion MRI ☐ Used ☒ Not used

Preprocessing

Preprocessing software | *Provide detail on software version and revision number and on specific parameters (model/functions, brain extraction, segmentation, smoothing kernel size, etc.).*

Normalization | *If data were normalized/standardized, describe the approach(es): specify linear or non-linear and define image types used for transformation OR indicate that data were not normalized and explain rationale for lack of normalization.*

Normalization template | *Describe the template used for normalization/transformation, specifying subject space or group standardized space (e.g. original Talairach, MNI305, ICBM152) OR indicate that the data were not normalized.*

Noise and artifact removal | *Describe your procedure(s) for artifact and structured noise removal, specifying motion parameters, tissue signals and physiological signals (heart rate, respiration).*

Volume censoring | *Define your software and/or method and criteria for volume censoring, and state the extent of such censoring.*

Statistical modeling & inference

Model type and settings | *Specify type (mass univariate, multivariate, RSA, predictive, etc.) and describe essential details of the model at the first and second levels (e.g. fixed, random or mixed effects; drift or auto-correlation).*

Effect(s) tested | *Define precise effect in terms of the task or stimulus conditions instead of psychological concepts and indicate whether ANOVA or factorial designs were used.*

Specify type of analysis: ☐ Whole brain ☐ ROI-based ☐ Both

Statistic type for inference
(See Eklund et al. 2016) | *Specify voxel-wise or cluster-wise and report all relevant parameters for cluster-wise methods.*

Correction | *Describe the type of correction and how it is obtained for multiple comparisons (e.g. FWE, FDR, permutation or Monte Carlo).*

Models & analysis

n/a	Involved in the study
☒	☐ Functional and/or effective connectivity
☒	☐ Graph analysis
☒	☐ Multivariate modeling or predictive analysis
