# Supplementary figures and images for: COX17 acetylation via MOF–KANSL complex promotes mitochondrial integrity and function
Source: Nat Metab. 2023 Oct 9;5(11):1931–52. doi: 10.1038/s42255-023-00904-w (PMC10663164; doi:10.1038/s42255-023-00904-w)

**Figure 1h**

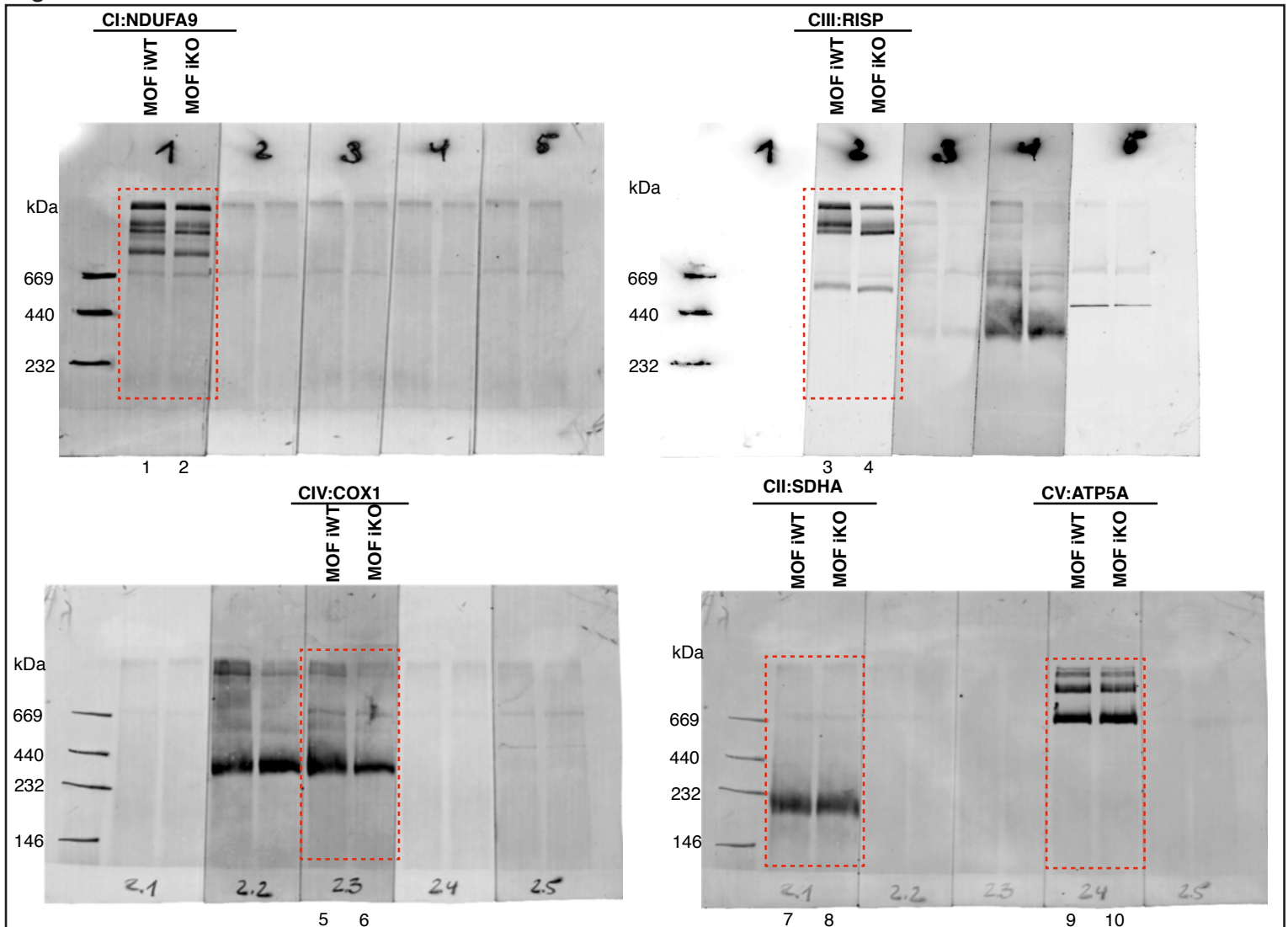

**Figure 1i**

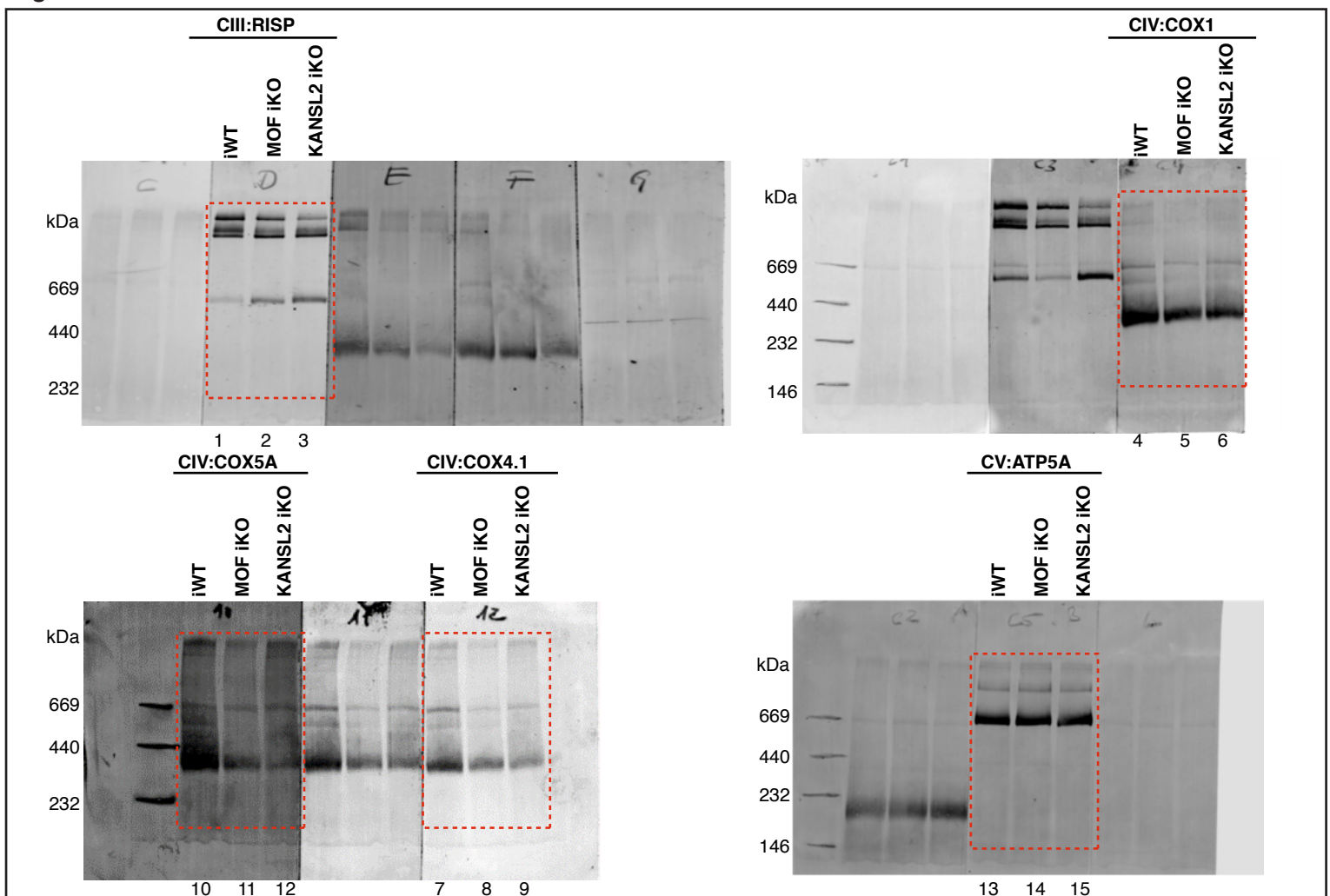

Supplement: Supplementary file 19 — Unprocessed western blots. [file 42255_2023_904_MOESM19_ESM.pdf]

Figure 2e

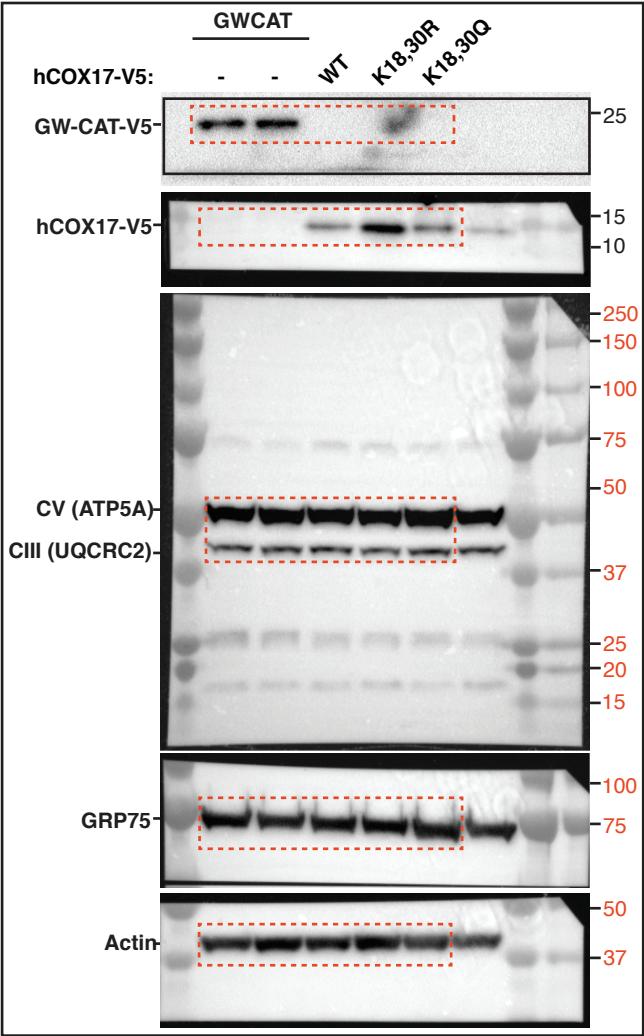

Supplement: Supplementary file 20 — Unprocessed western blots. [file 42255_2023_904_MOESM20_ESM.pdf]

**Figure 3c**

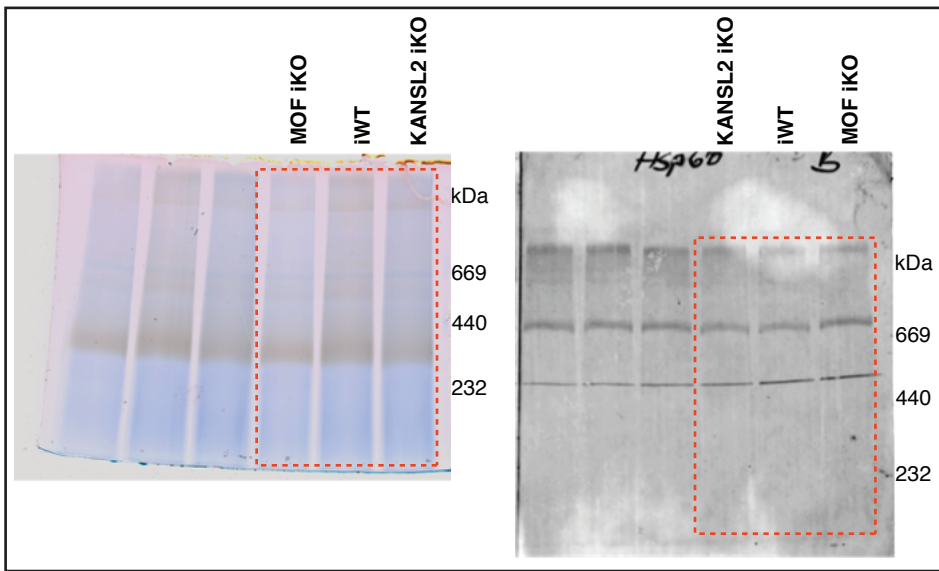

**Figure 3f**

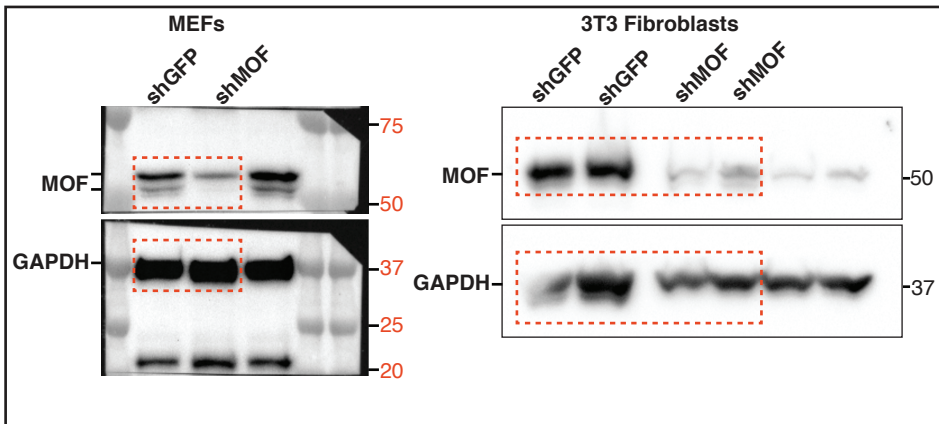

Supplement: Supplementary file 21 — Unprocessed western blots. [file 42255_2023_904_MOESM21_ESM.pdf]

Figure 4k

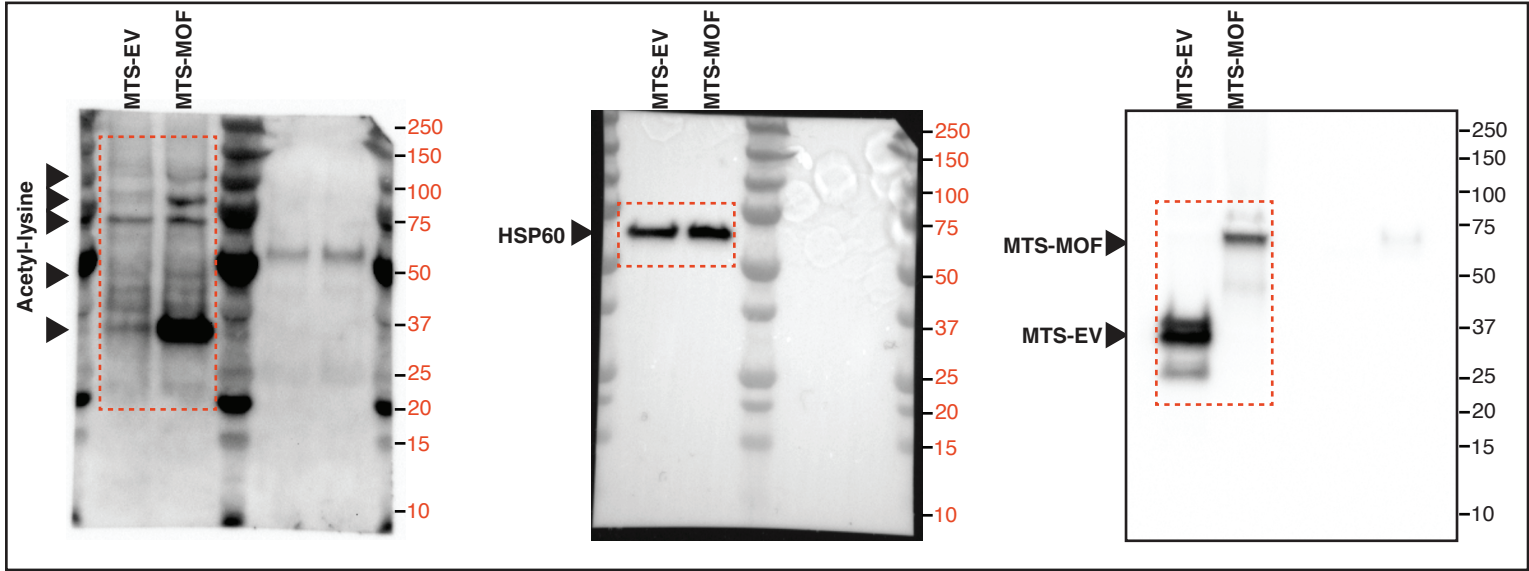

Supplement: Supplementary file 22 — Unprocessed western blots. [file 42255_2023_904_MOESM22_ESM.pdf]

Figure 5a

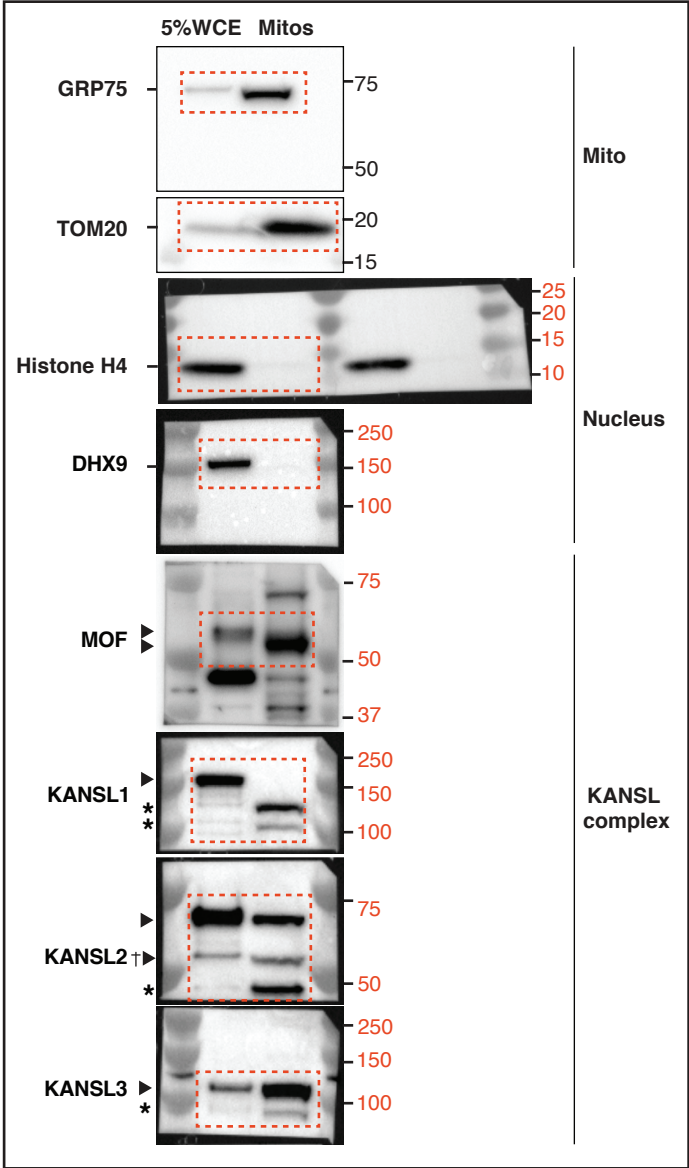

Figure 5c

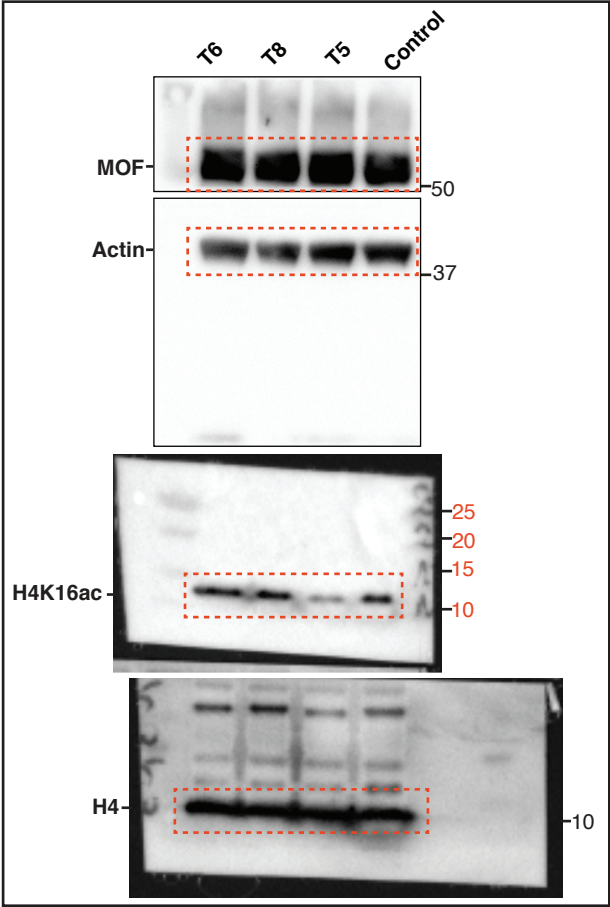

Figure 5i

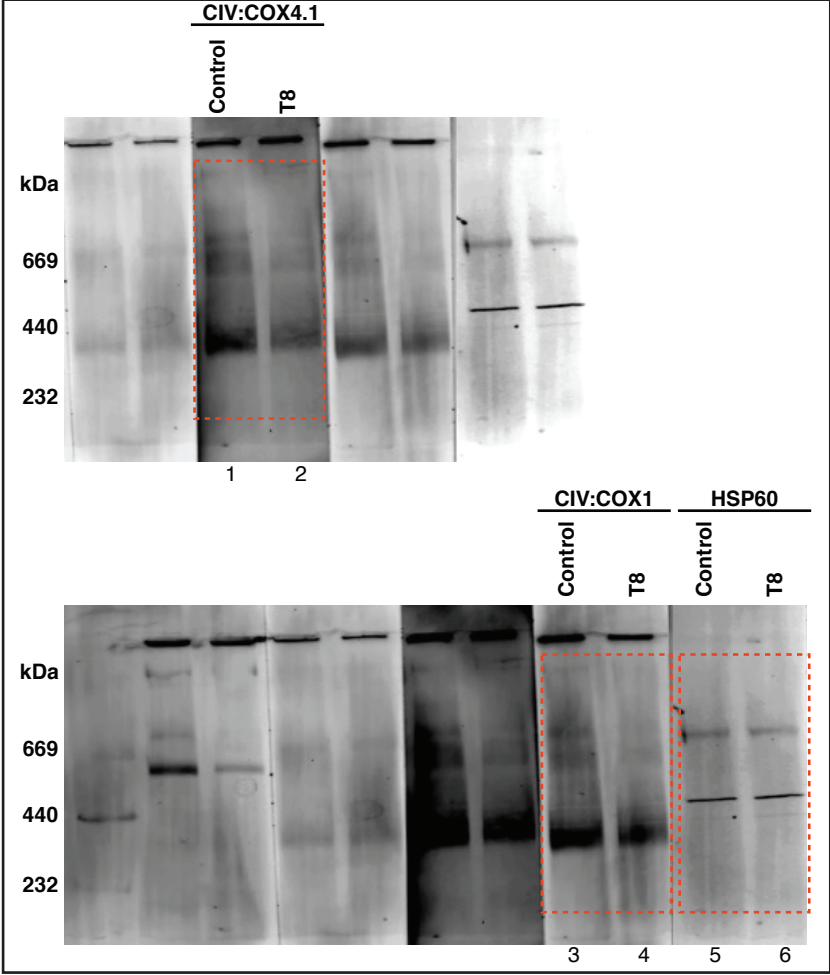

Figure 5f

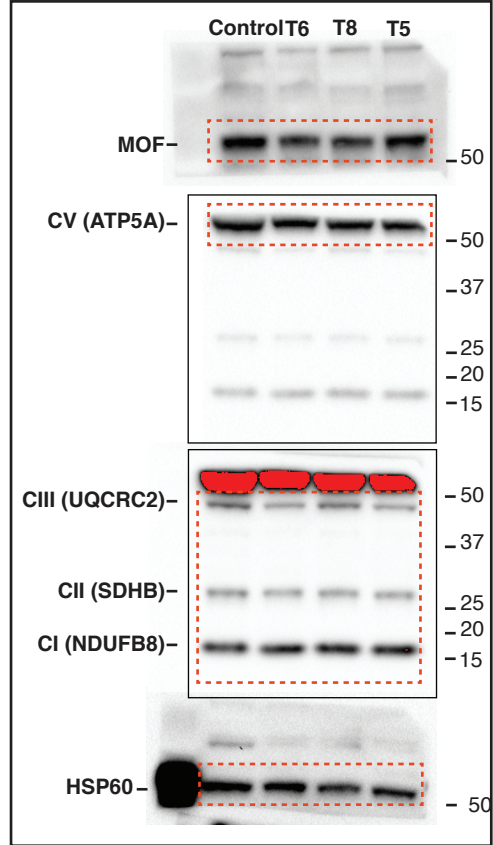

Supplement: Supplementary file 23 — Unprocessed western blots. [file 42255_2023_904_MOESM23_ESM.pdf]

Figure 6c

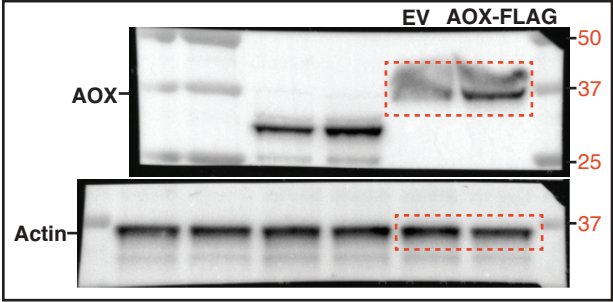

Figure 6d

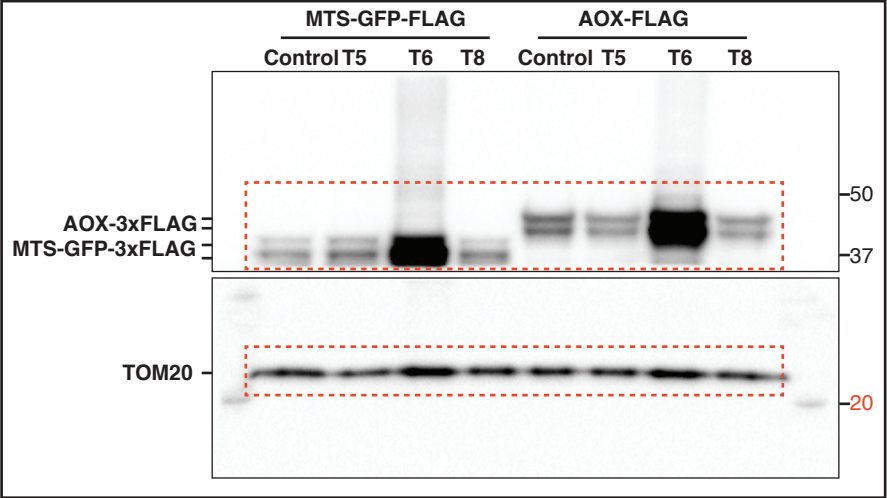

Supplement: Supplementary file 24 — Unprocessed western blots. [file 42255_2023_904_MOESM24_ESM.pdf]

Figure 7b

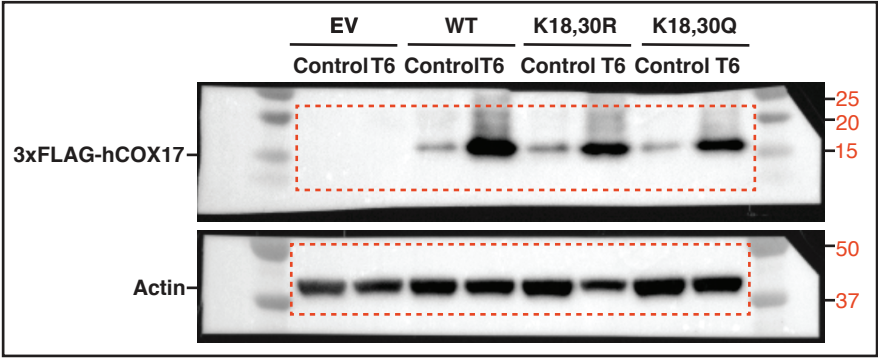

Supplement: Supplementary file 25 — Unprocessed western blots. [file 42255_2023_904_MOESM25_ESM.pdf]

Figure ED1a (upper panel)

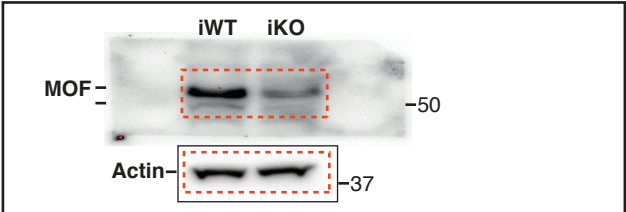

Figure ED1a (center panel)

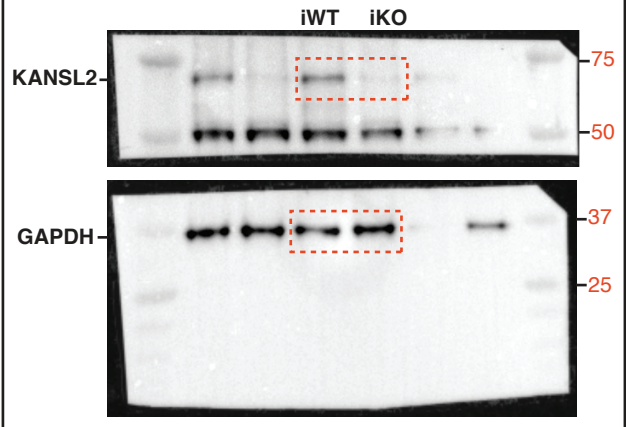

Figure ED1a (lower panel)

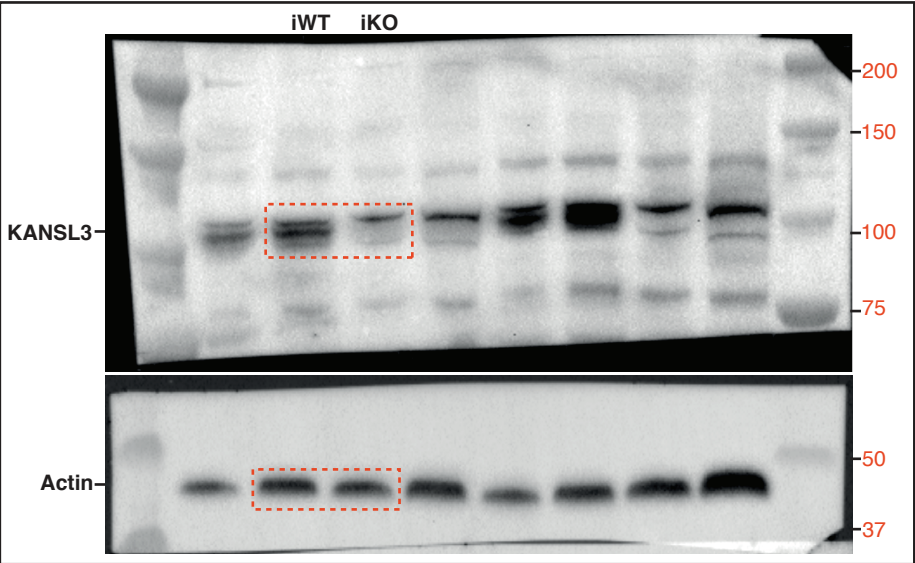

Figure ED1k

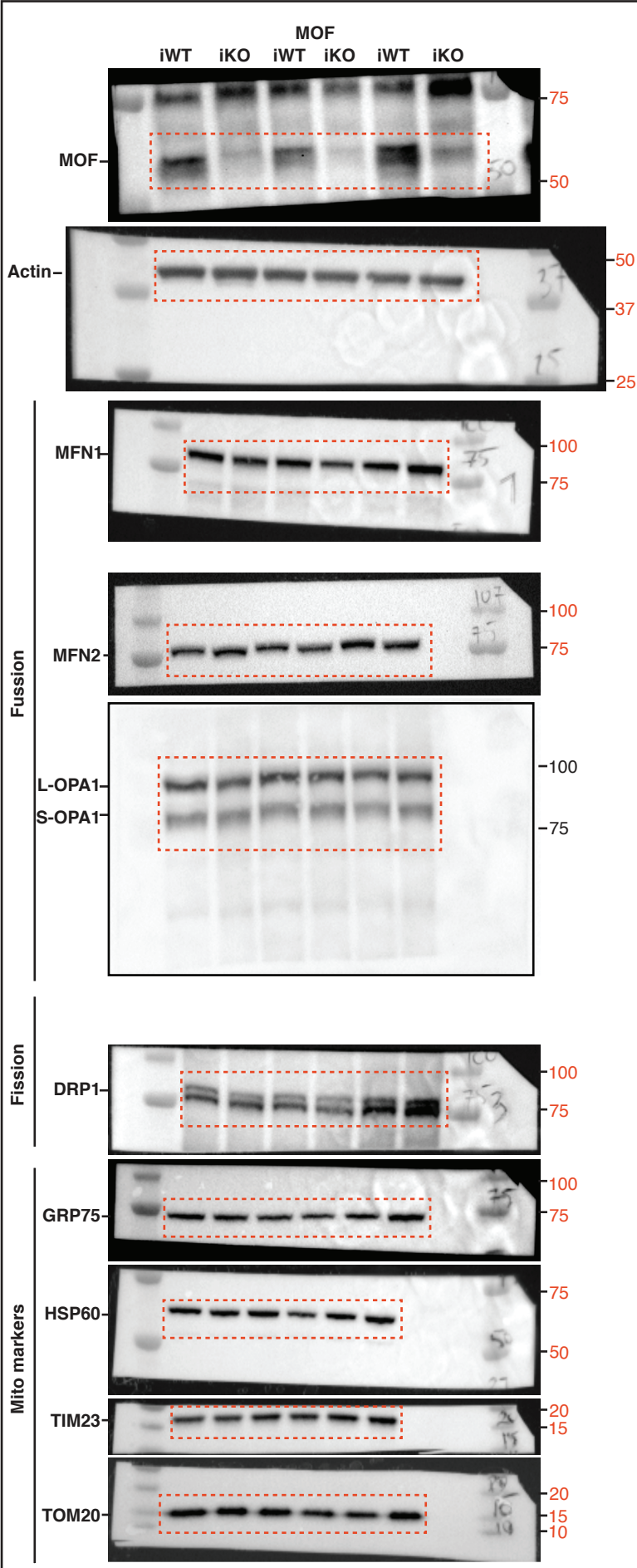

Figure ED1l

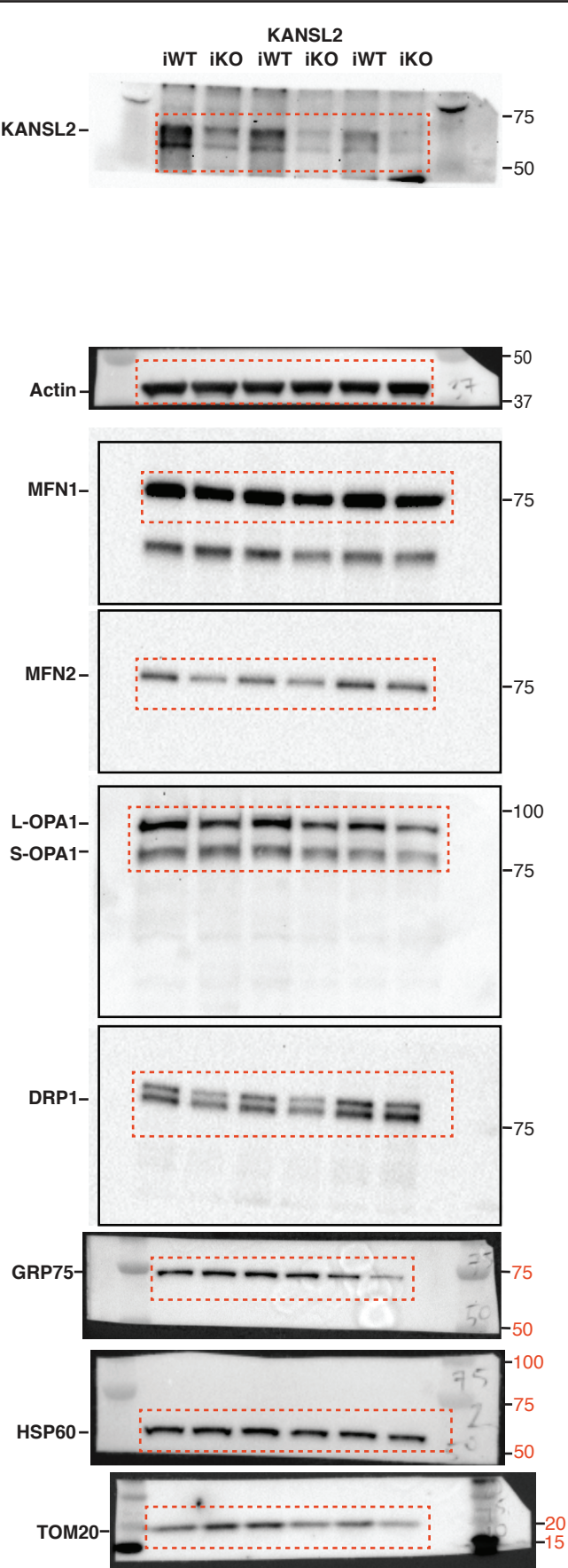

Supplement: Supplementary file 26 — Unprocessed western blots. [file 42255_2023_904_MOESM26_ESM.pdf]

Figure ED2g

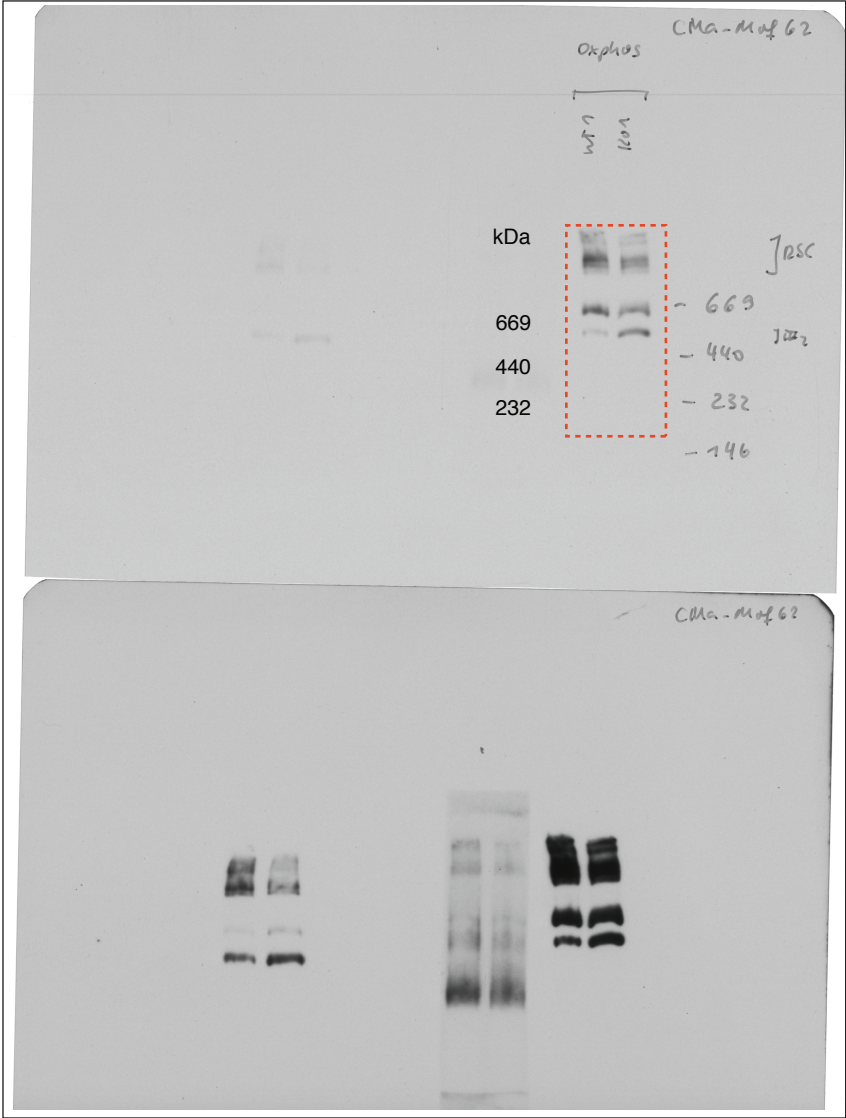

Figure ED2h

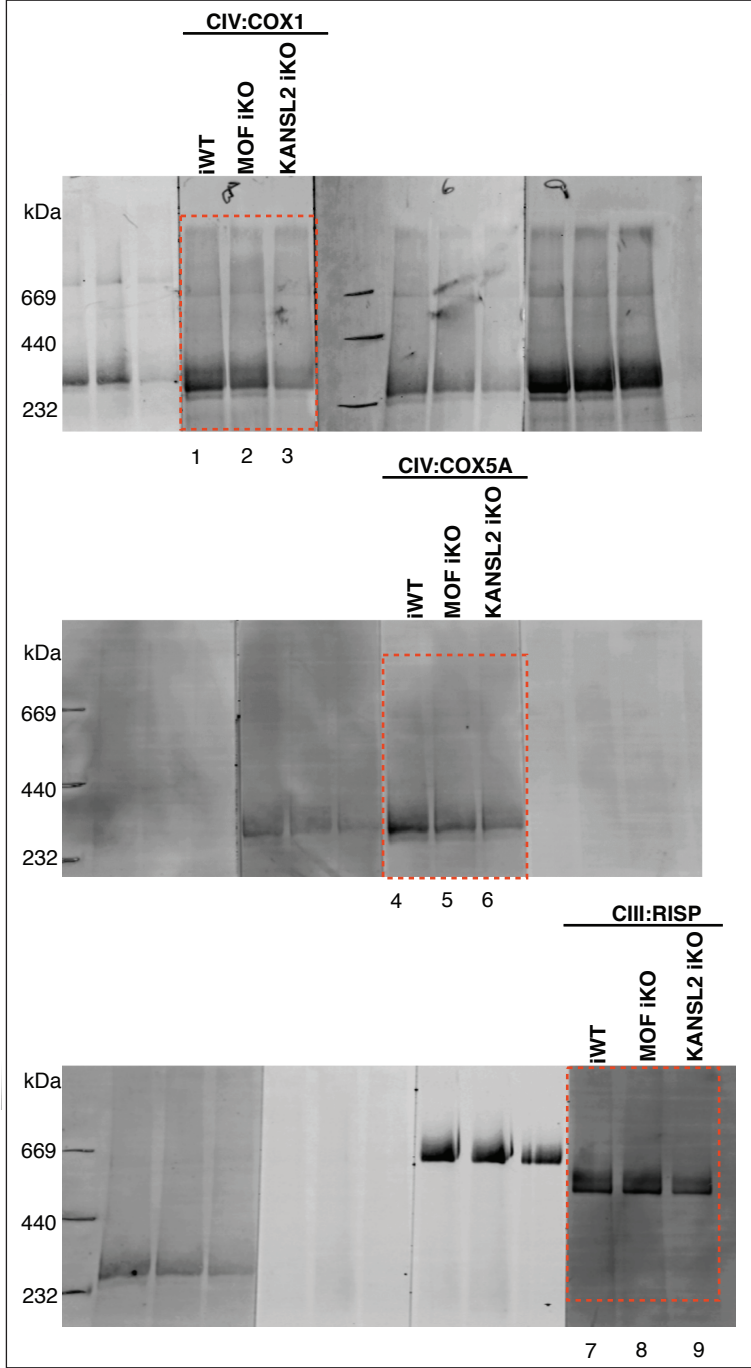

Figure ED2j

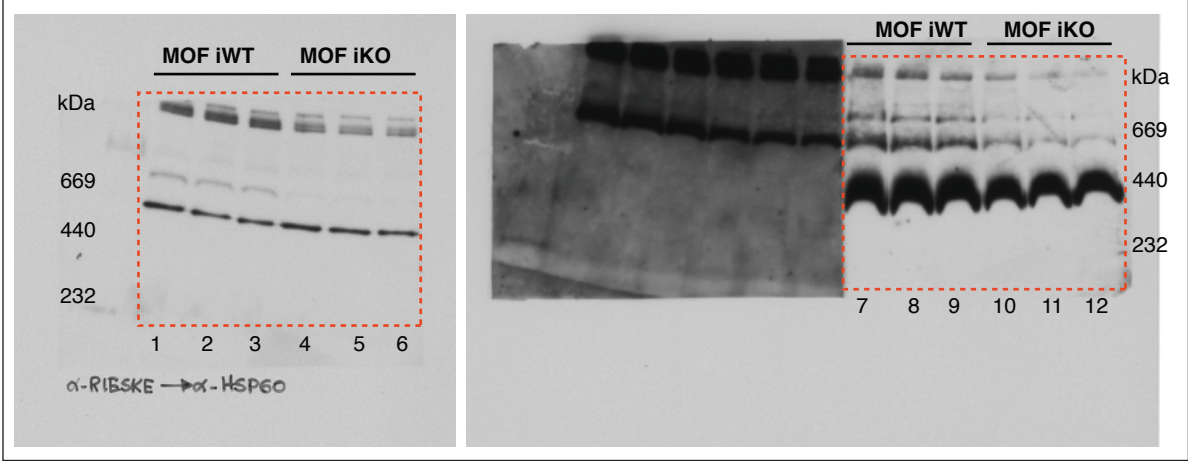

Supplement: Supplementary file 27 — Unprocessed western blots. [file 42255_2023_904_MOESM27_ESM.pdf]

Figure ED4a (left panel)

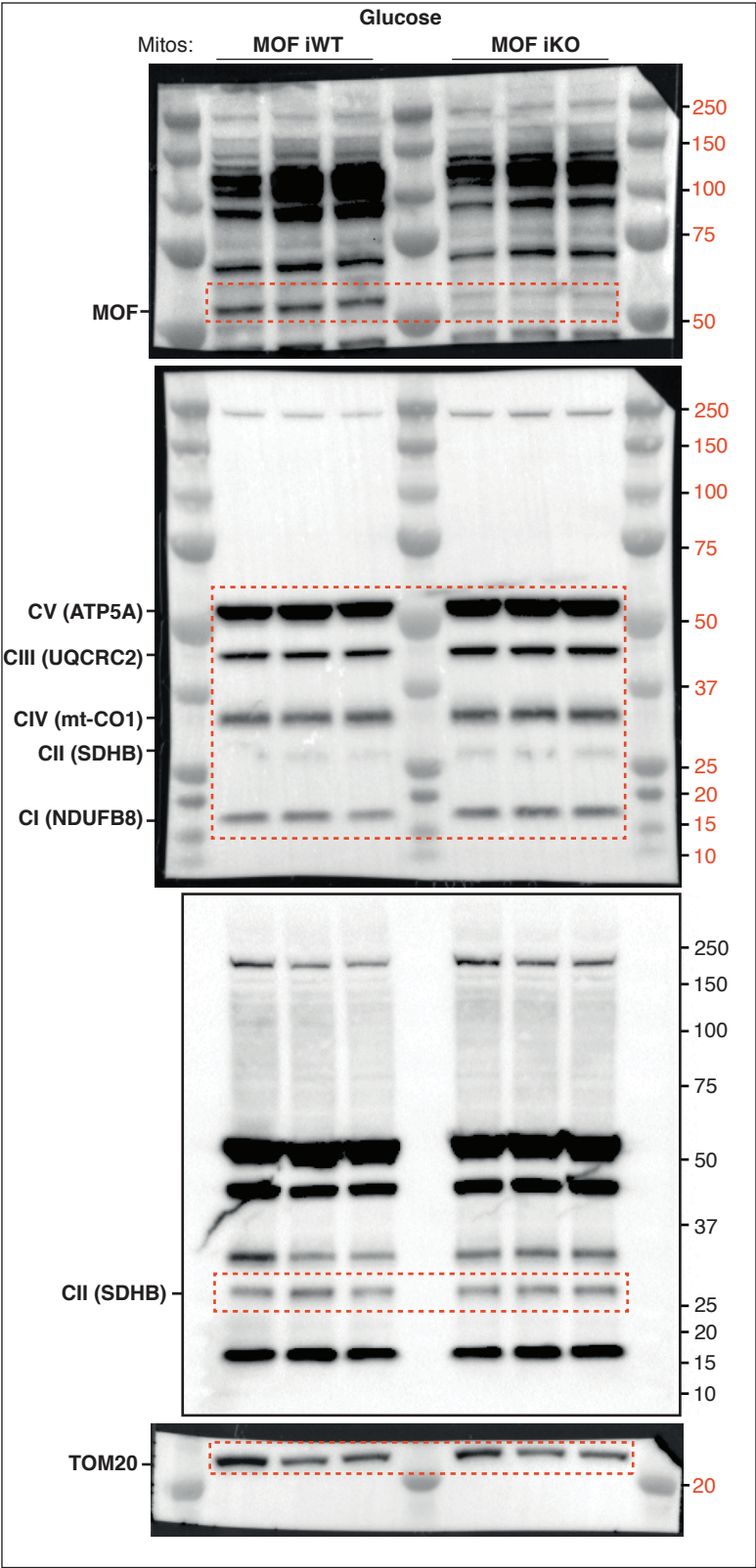

Figure ED4a (right panel)

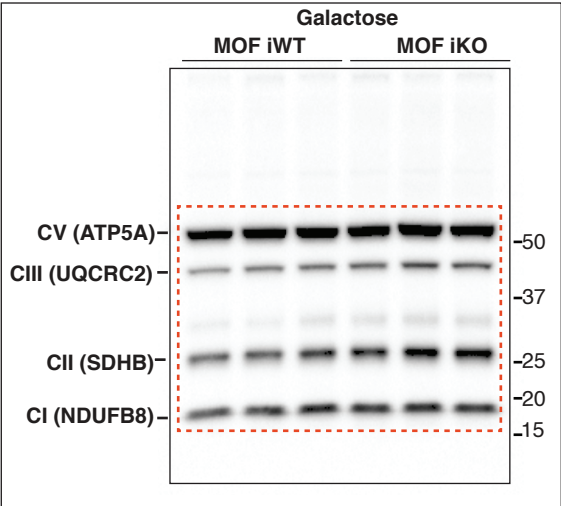

Figure ED4b

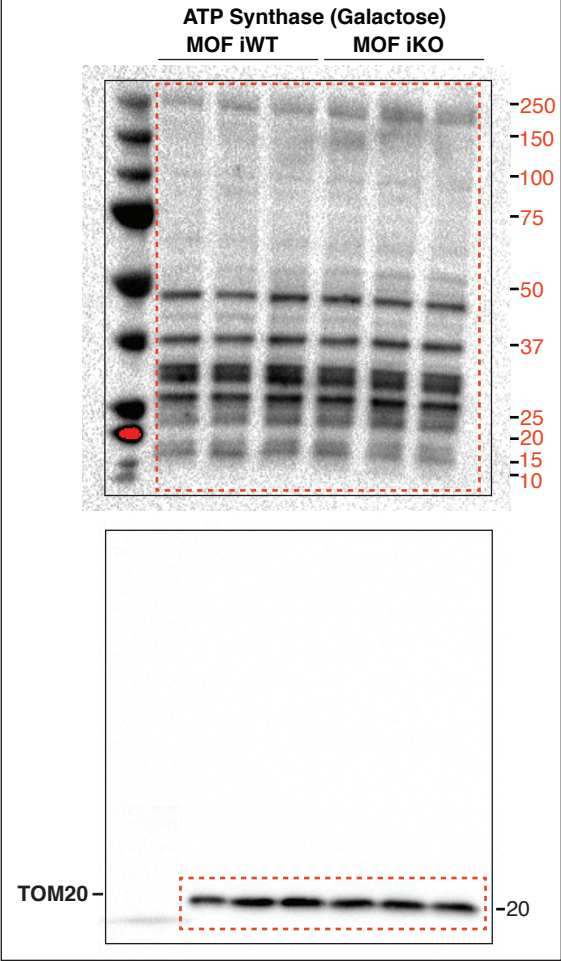

Supplement: Supplementary file 28 — Unprocessed western blots. [file 42255_2023_904_MOESM28_ESM.pdf]

Figure ED5a

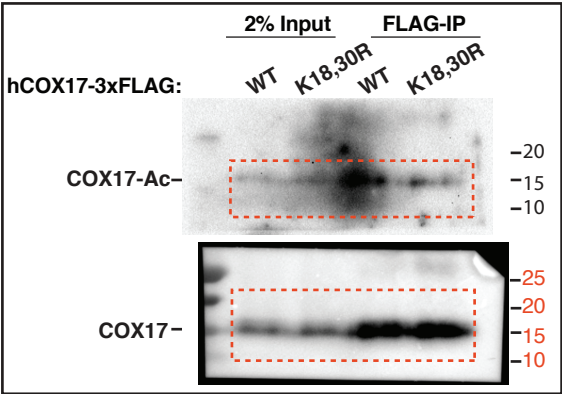

Figure ED5c

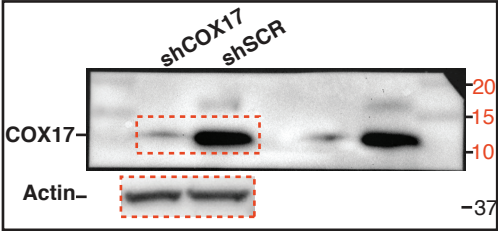

Figure ED5g

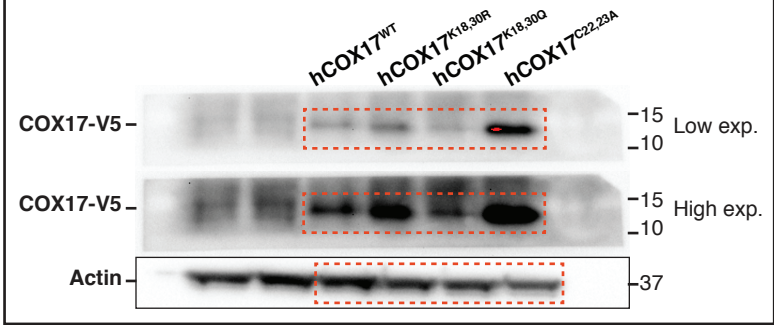

Figure ED5k

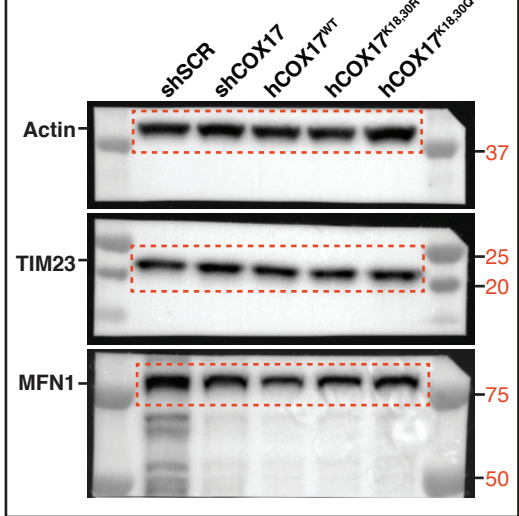

Figure ED5h

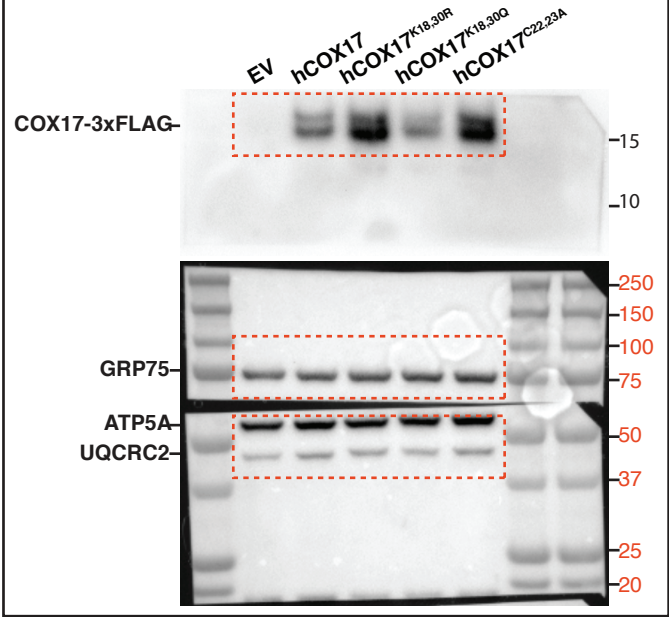

Figure ED5l

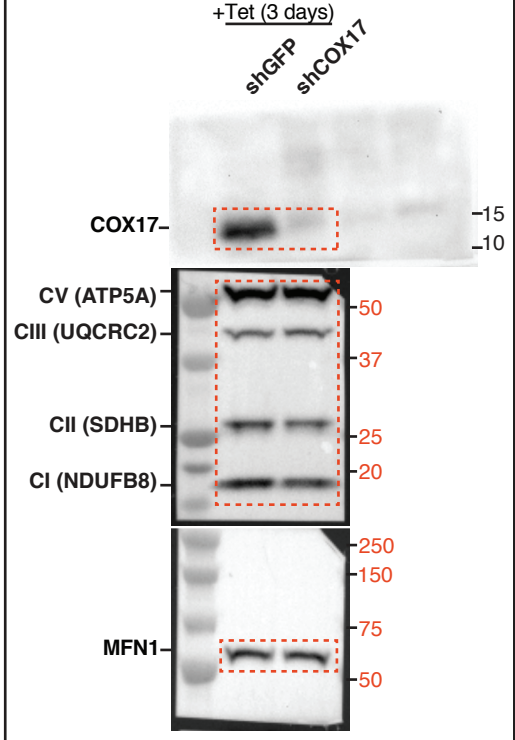

Supplement: Supplementary file 29 — Unprocessed western blots. [file 42255_2023_904_MOESM29_ESM.pdf]

Figure ED6a

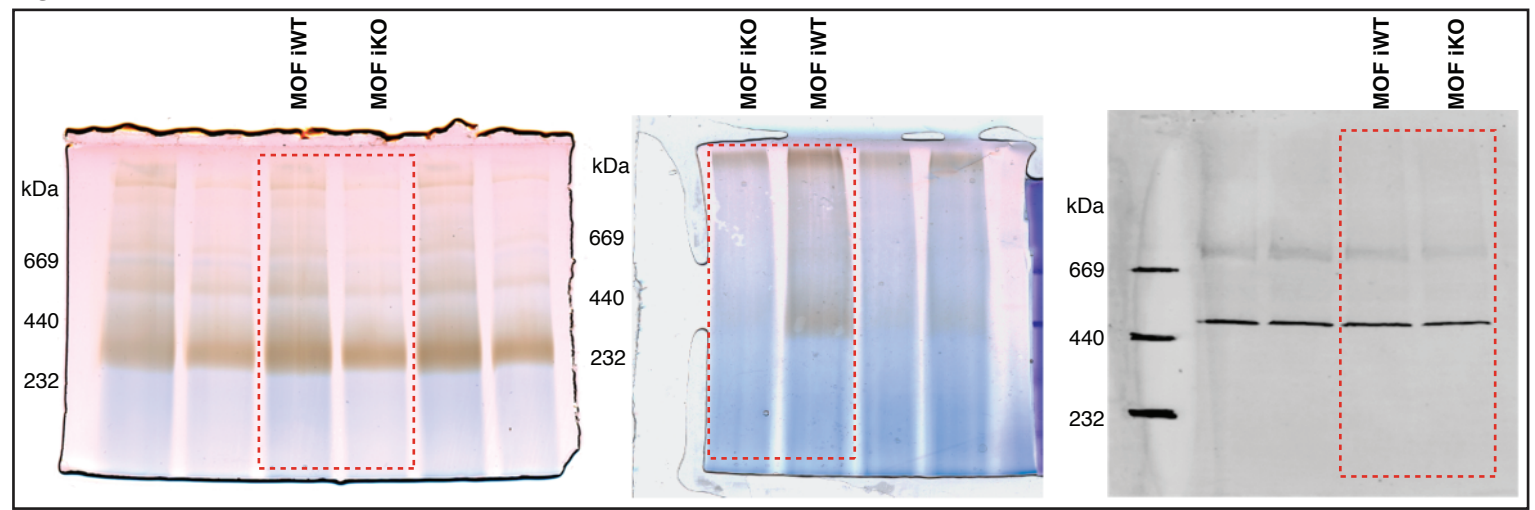

Figure ED6d

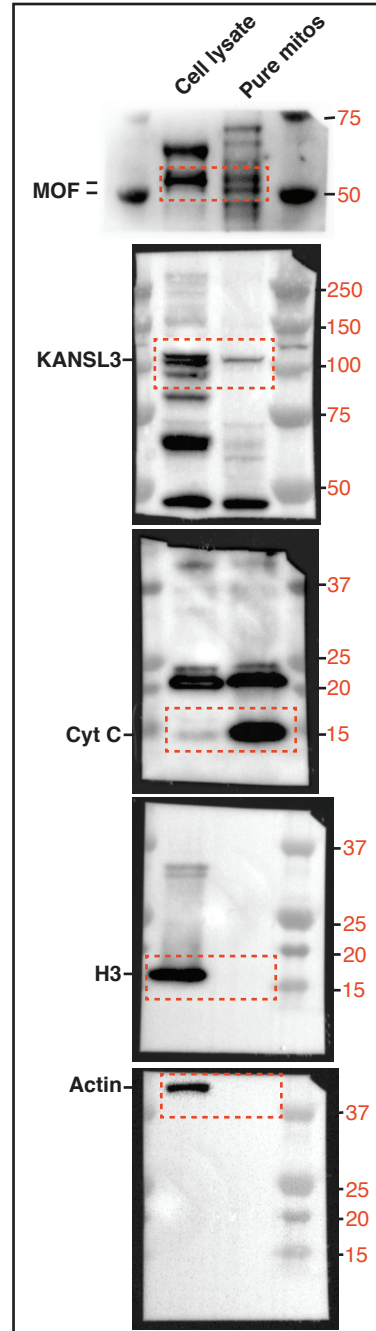

Figure ED6b

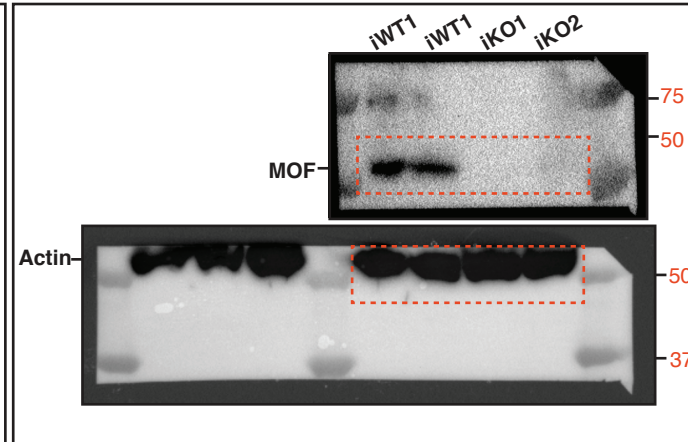

Figure ED6e

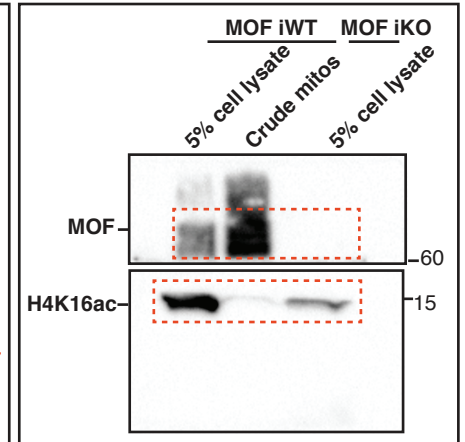

Figure ED6g

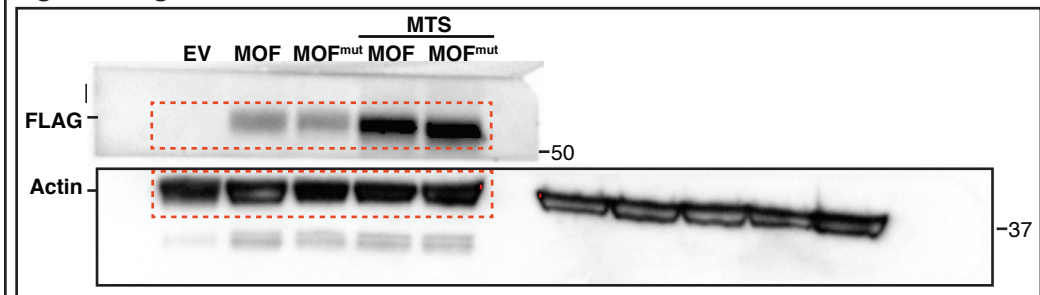

Figure ED6i

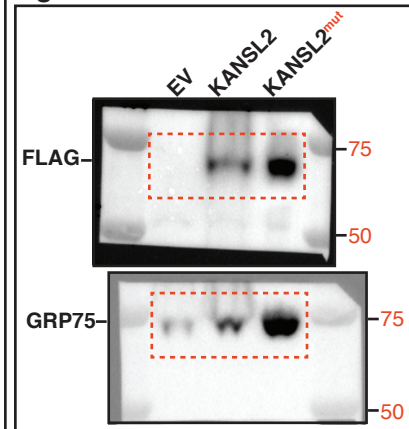

Supplement: Supplementary file 30 — Unprocessed western blots. [file 42255_2023_904_MOESM30_ESM.pdf]
